# Artificial neural networks fighting real neural decline: a systematic review of AI in Alzheimer’s research

**DOI:** 10.1007/s10462-025-11484-4

**Published:** 2026-02-02

**Authors:** Farzana Sharmin Mou, Tanvir Ahmed, Md Nazmul Huda, Asoke K. Nandi

**Affiliations:** 1 Department of Electronic and Electrical Engineering, Brunel University of London, Uxbridge, Middlesex, UB8 3PH UK; 2 Department of Computer Science and Engineering, Dhaka University of Engineering and Technology, Gazipur, 1707 Bangladesh; 3 School of Information and Communication Engineering, North University of China, Taiyuan, China

**Keywords:** Alzheimer’s disease, Artificial intelligence, Machine learning, Multi-modality, Early detection, Disease progression

## Abstract

Alzheimer’s disease (AD) is a major global health challenge, with Artificial Intelligence (AI) increasingly recognized as a transformative tool for early detection, disease progression modeling, and therapeutic discovery. This systematic review, conducted in accordance with PRISMA guidelines, analyzed 156 peer-reviewed studies published between 2010 and 2024, identified from four major databases (Scopus, PubMed, Web of Science, IEEE Xplore). A particular emphasis was placed on multimodal approaches that integrate neuroimaging, genetics, biomarkers, and clinical data to improve accuracy and translational value. To organize this fragmented field, we introduce a novel Layered Framework that categorizes AI applications into four domains: Early Detection, Disease Progression Modeling, Therapeutic Discovery, and Real-World Integration. In addition, we applied ARIMA-based forecasting to project research trajectories through 2030, which revealed generative models and transformer architectures as the fastest-growing and most promising methodologies. The review highlights substantial advances in early detection and multimodal fusion, particularly through deep learning, while also identifying persistent challenges such as limited model generalizability, ethical concerns, and underexplored clinical implementation. Addressing these barriers will require multi-cohort validation, interpretable AI, and equity-driven model development. By consolidating evidence and forecasting future directions, this review provides a roadmap for accelerating precision-driven innovations in Alzheimer’s care.

## Introduction

### The growing burden of AD

Alzheimer’s disease (AD) is a progressive neurodegenerative disorder characterized by cognitive decline, behavioral changes, and functional impairment (Sadik and Wilcock 2003). The global prevalence of AD is expected to quadruple by 2050, with one in 85 people affected worldwide (Brookmeyer et al. 2007). The percentage of US adults with Alzheimer’s disease and related dementias is projected to grow from 1.6% in 2014 to 3.3% in 2060 (Kuehn 2018). Age is the primary risk factor, followed by the apolipoprotein E-4 allele, female sex, head trauma history, and cerebrovascular risk factors (Lopez 2011). The neuropathological hallmarks of AD include amyloid plaques, neurofibrillary tangles, and neuronal loss, primarily affecting the limbic system and acetylcholine neurotransmission (Lopez 2011). The increasing burden of AD poses significant challenges for caregivers and healthcare systems (Han 2014; Sadik and Wilcock 2003). However, even relatively small advances in therapeutic and preventive strategies that delay disease onset and progression by even one year could significantly reduce the global burden, potentially preventing 9.2 million cases by 2050 (Brookmeyer et al. 2007).

In the context of this review, the term neural decline is used to denote the biological and physiological deterioration of brain structures and networks that underpin the clinical manifestations of Alzheimer’s disease. While cognitive decline refers to observable impairments in memory, reasoning, and executive function, neural decline emphasizes the underlying neurobiological alterations—such as neuronal loss, synaptic dysfunction, and disrupted connectivity—detectable through neuroimaging and biomarker analyses. This terminology reflects the focus of the present work on AI-driven approaches that characterize and predict such brain-level changes, providing insight into the mechanisms of Alzheimer’s progression beyond behavioral outcomes.

### The rise of neural networks and AI in medicine

Neural networks and AI have emerged as powerful tools in medicine, revolutionizing various aspects of healthcare (Berdutin et al. 2023). These technologies offer significant potential in disease prediction, risk assessment, and diagnostic support (Berdutin et al. 2023; Mansouri 2023). Artificial Neural Networks (ANNs) have found applications in cardiology, diagnostics, medical imaging, and radiology (Mansouri 2023). Convolutional neural networks, auto-encoders, and recurrent neural networks are being utilized for tasks such as medical image segmentation and arrhythmia detection (Hireš et al. 2022). Neural networks excel at finding complex relationships in large datasets, making them particularly useful in processing the vast amounts of diagnostic data generated by modern medical technologies (Lewin 2000). While human expertise remains crucial, neural networks are increasingly assisting in diagnosis and decision-making, potentially improving accuracy and efficiency in healthcare (Hireš et al. 2022; Lewin 2000). Fourcade and Khonsari conducted a systematic review (Fourcade and Khonsari 2019) on the use of convolutional neural networks (CNNs) for medical image analysis. They concluded that CNNs can complement and optimize the work of medical professionals, particularly in visual-based specialties such as radiology and pathology. However, concerns persist regarding the interpretability of AI systems and the need for further research on their reliability (Berdutin et al. 2023).

**Fig. 1:**
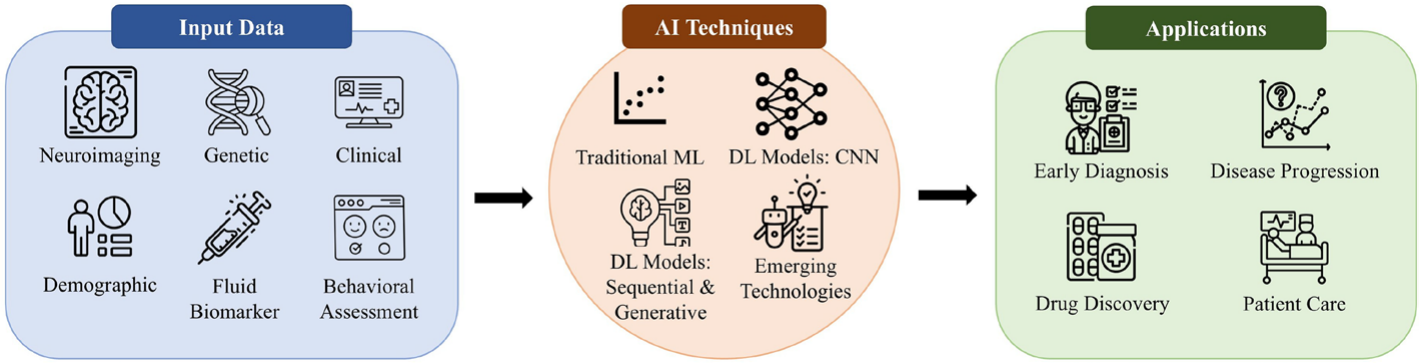
Conceptual framework for AI in Alzheimer’s research

### Conceptual framework for AI applications in AD

To provide a structured understanding of AI’s role in Alzheimer’s research, we propose a conceptual framework (Fig. 1) that outlines the interaction between data inputs, AI techniques, and their applications.

- **Input data**: The foundation of AI applications includes a wide range of inputs such as neuroimaging data (MRI, PET scans), fluid biomarkers (CSF, blood-based markers), genetic information (e.g., APOE genotype), and cognitive and behavioral assessments (Bazarbekov et al. 2024; Zhao et al. 2023a).
- **AI techniques**: This stage involves a range of computational methods tailored for different data types and research questions. These can be grouped into four main categories as shown in Fig. 1:
  - *Traditional ML*: This includes foundational machine learning algorithms (Zhao et al. 2023b) like Support Vector Machines (SVMs) and Random Forests, which are highly effective for classification and regression tasks using structured data, such as clinical, genetic, and demographic information.
  - *Deep learning models*: *CNNs*: Deep learning models are central to modern approaches. Convolutional Neural Networks (CNNs) are the cornerstone of neuroimaging analysis, designed to automatically learn spatial features from MRI and PET scans for tasks like diagnosis and biomarker identification (Ajay et al. 2023; Shourie et al. 2024).
  - *Deep learning models*: *Sequential and Generative*: This category includes other key deep learning architectures. Sequential models (e.g., Recurrent Neural Networks) are vital for analyzing longitudinal data to model disease progression over time (DiPietro and Hager 2020). Generative models (e.g., GANs, VAEs) can be used to synthesize realistic medical data to augment training sets or discover novel molecular structures for drug discovery (Alam and Latifi 2025).
  - *Emerging technologies*: This represents the cutting edge of AI in AD research, encompassing both novel architectures and methodologies. It includes state-of-the-art models like Transformers (Yu et al. 2024) and Vision Transformers (ViTs), which are setting new performance benchmarks for analyzing sequential clinical data and neuroimaging, respectively. This category also covers crucial methodologies like Explainable AI (XAI) to make model decisions transparent (Vimbi et al. 2024) and Federated Learning (Basnin et al. 2025) to train models on data from multiple sites without compromising patient privacy.
- **Applications**: These techniques are applied in four key areas: early diagnosis, disease progression modeling, drug discovery, and patient care, with the ultimate goal of improving clinical outcomes and enhancing patient quality of life (Angelucci et al. 2024).

### Objective of the review

The primary objective of this review is to systematically evaluate the application of artificial intelligence (AI) in Alzheimer’s Disease (AD) research, with a particular focus on multimodal approaches that integrate imaging, clinical, and genetic data. Our review provides a comprehensive analysis of trends, techniques, and applications, while identifying key challenges and opportunities for future research.

We first examine trends and patterns in AI research for AD, summarizing publication growth over the past 15 years (2010–2024) and comparing general AI approaches with multimodal methodologies. We also analyze the timeline of AI techniques applied to AD research, assess the distribution of studies across four key layers of AI application, and offer a forward-looking forecast of emerging methodologies and research gaps through 2030 using ARIMA-based time series models.

Next, we delineate the scope of AI applications, systematically categorizing methods such as machine learning, deep learning, and neural networks across four critical domains: early detection, disease progression modeling, therapeutic discovery, and real-world patient care. We highlight how these techniques leverage multimodal data to enhance diagnostic accuracy, predictive modeling, and personalized intervention strategies.

Finally, we address challenges and opportunities, discussing limitations in data availability, model generalizability, algorithmic bias, and clinical integration. By identifying gaps and proposing actionable future directions, this review aims to provide a strategic, forward-looking perspective that advances the role of AI in combating AD, emphasizing multimodal innovation and research forecasting.

### Comparison with the newest reviews and novel contributions

Several recent review studies have contributed significantly to understanding the application of AI in AD, each addressing various facets of detection, diagnosis, and classification. For example, this study (Mohsen 2025) provides a broad overview of machine learning (ML) and deep learning (DL) approaches for AD classification from brain MRIs, including discussions on overfitting, latency, and real-world deployment. Similarly, another review (Biswas et al. 2025) focuses on the comparative performance of ML, DL, and statistical techniques for early AD detection using neuroimaging and highlights challenges like data scarcity and interpretability.

These authors (Chamakuri and Janapana 2025) systematically review DL techniques using a PRISMA framework and expose research gaps in automatic AD detection. In contrast, (Kaur and Sachdeva 2025) narrow their scope to recent trends (2019–2023), comparing architectures and performance metrics from a practitioner’s standpoint. A unique contribution by (Mirabian et al. 2025) lies in differentiating AD from frontotemporal dementia (FTD) using AI on imaging biomarkers, while (Hechkel and Helali 2025) offering a roadmap for MRI-based diagnosis and highlighting transfer learning and dataset usage patterns.

While these reviews collectively offer valuable insights into algorithmic advancements, imaging modalities, and evaluation metrics, they primarily focus on either:

- Narrow diagnostic windows (e.g., early detection),
- Single-modality frameworks,
- Architecture-based categorization (e.g., CNNs, autoencoders),
- Or comparisons without structured frameworks for longitudinal understanding.

In contrast, our review provides several novel contributions:

1. **Layered framework**: We introduce a four-tiered framework: Early Detection, Disease Progression Modeling, Therapeutic Discovery, and Real-World Integration, offering a structured categorization of AI applications across the full AD continuum. This layered view enables a holistic understanding of research maturity and gaps across domains.
2. **Multi-modality focus**: Rather than isolating imaging or genetic methods, we critically analyze how integrative AI models leveraging clinical, neuroimaging, and genetic data improve diagnostic and prognostic capabilities, an area underexplored in prior reviews.
3. **Forecasting Analysis**: We present a novel forecasting trajectory using ARIMA models, projecting AI publication trends in AD through 2030. This predictive analysis identifies emerging growth areas (e.g., generative models, transformer-based architectures) and provides a future-oriented roadmap for researchers.
4. **Methodological rigor**: Our review synthesizes 156 studies from four leading databases (Scopus, PubMed, Web of Science, IEEE Xplore), applying strict inclusion criteria and offering data-driven benchmarking across modalities and architectures.
5. **Research gaps and strategic directions**: We identify persistent challenges, such as lack of multimodal interpretability, model generalizability, and equitable dataset representation, and offer actionable strategies like equity-driven AI development, multi-cohort validation, and real-world deployment frameworks.

By consolidating a decade of advancements and aligning them within a predictive and layered paradigm, our review transcends traditional surveys and offers a strategic, forward-looking synthesis of AI’s role in Alzheimer’s research.

## Methods

### Systematic review framework

This systematic review was conducted following the *Preferred Reporting Items for Systematic Reviews and Meta-Analyses (PRISMA)* guidelines (Page et al. 2021). A comprehensive search strategy was designed to identify relevant studies focusing on the use of Artificial Intelligence (AI) in Alzheimer’s disease (AD) research.

Four databases were searched: **Scopus**, **PubMed**, **Web of Science**, and **IEEE Xplore**. The search period spanned from 2010 to 2024, and the query was constructed to capture a wide range of studies related to AI applications in AD, including diagnosis, prediction, detection, monitoring, treatment, and drug discovery.

The search query was:

(“Artificial Intelligence” OR “Machine Learning” OR “Deep Learning” OR “Neural Networks”) AND (“Alzheimer’s Disease” OR “Alzheimer”) AND (diagnosis OR prediction OR detection OR monitoring OR treatment OR “drug discovery”) AND (“multi-modality” OR “multimodality” OR “multi modality”)

**Fig. 2:**
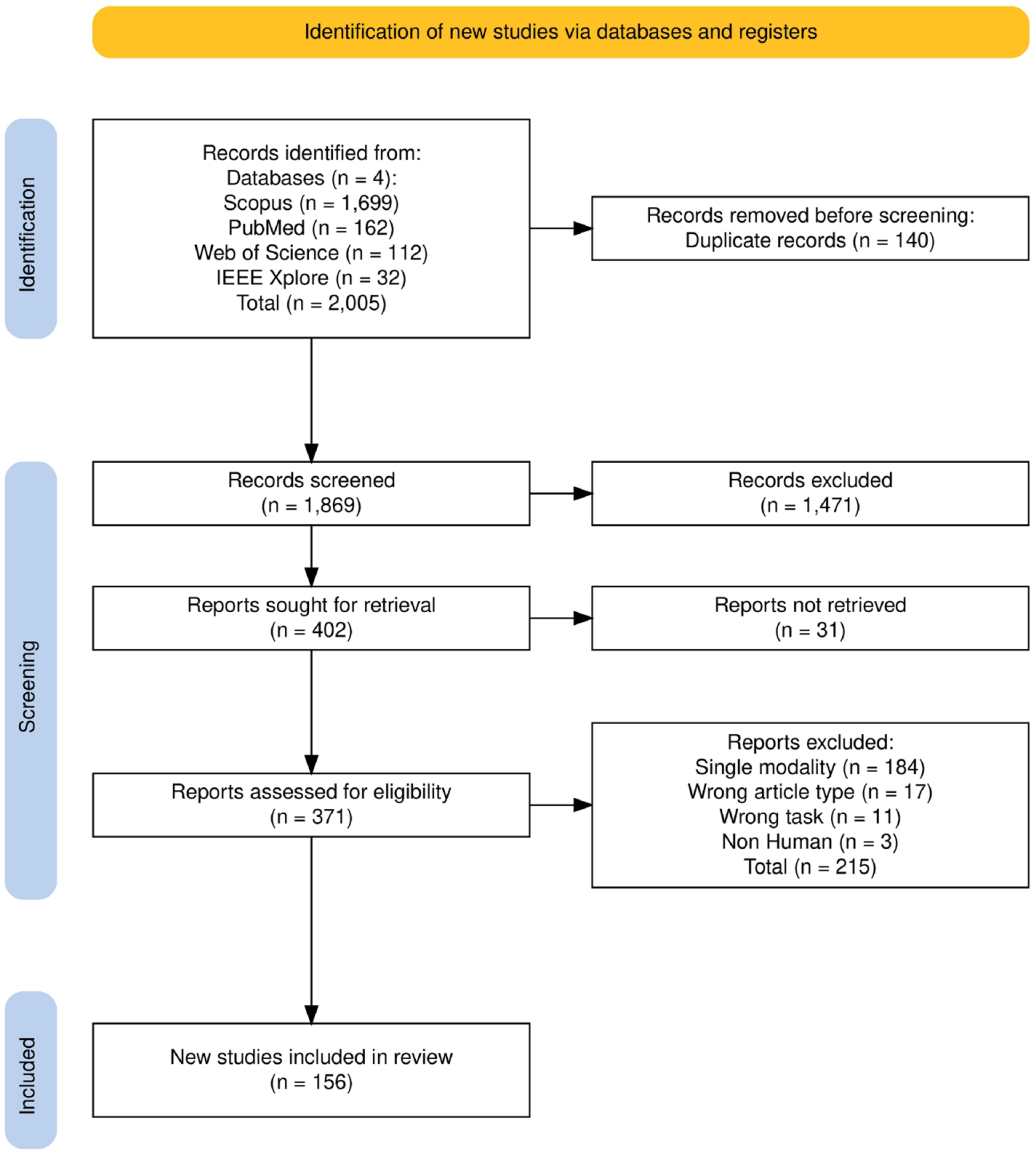
PRISMA 2020 flow diagram for systematic review outlining the number of studies identified and excluded at each stage

Database-specific adaptations to the query syntax were applied where necessary. The study selection process was documented step-by-step and is illustrated in Fig. 2, which summarizes identification, screening, eligibility, and inclusion stages.

### Inclusion and exclusion criteria

Studies were included in this review based on the following criteria:

**Population**: Studies focused on Alzheimer’s disease in human subjects.

**Time Frame**: Studies from 2010 to 2024 were considered to capture the most recent advancements.

**Intervention**: Studies utilizing AI techniques, including machine learning, deep learning, or neural networks.

**Outcomes**: Studies addressing diagnosis, progression prediction, detection, monitoring, treatment, or drug discovery.

**Modality**: For this review, “multi-modality” was defined as the integration of at least two distinct data sources, which could include multiple imaging techniques (e.g., MRI + PET), imaging combined with clinical, genetic, or cognitive assessments, or non-imaging combinations (e.g., text + audio).

**Study Design**: Peer-reviewed journal articles.

Studies were excluded if they met any of the following criteria:

- Duplicate records identified during the screening process.
- Single-modality approaches only.
- Wrong article type, such as editorials, commentaries, case reports, books, systematic reviews, and conference papers.
- Studies focusing on non-human subjects.
- Studies published in languages other than English.
- Irrelevant research tasks (e.g., unrelated AI applications).

A total of 2,005 records were initially identified. After removing duplicates (n = 140) using Zotero (Sean et al. 2006), 1,869 records were screened, leading to the final inclusion of 156 studies. Details of this process are shown in the PRISMA Flow Diagram Fig. 2. All titles, abstracts, and full texts were independently screened by multiple authors. Disagreements were resolved by discussion and consensus.

### Visual summary of methodology

**Fig. 3:**
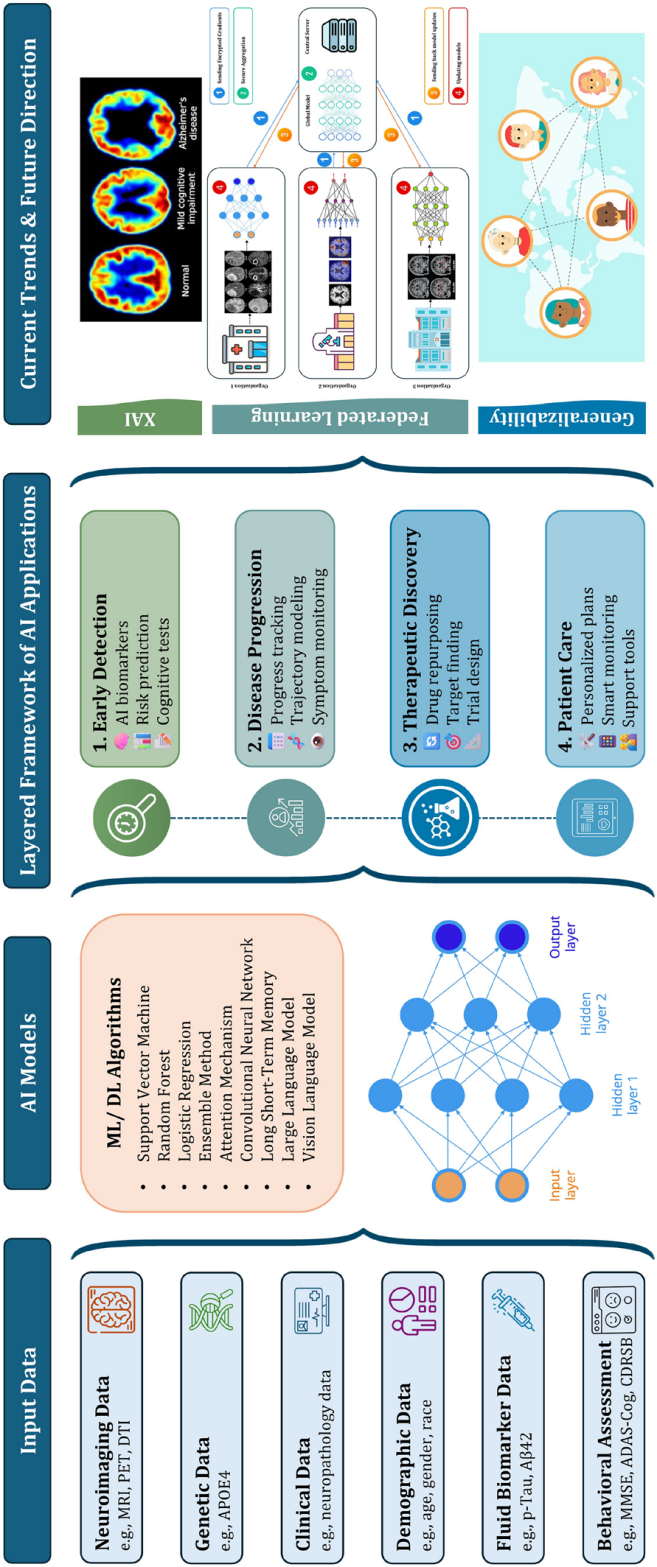
Schematic of the proposed four-layered framework for AI in AD, comprising early detection, disease progression, therapeutic discovery, and real-world integration. The diagram also illustrates the multimodality interplay (e.g., imaging, genetics, clinical, cognitive data) that supports each layer

To enhance the clarity and transparency of our systematic review methodology, a graphical summary is presented in Fig. 3. This illustration outlines the key components of the review workflow, including data modality categorization, artificial intelligence (AI) model typologies, the layered analytical framework employed, and identified future research directions. By providing a high-level visual overview, the schematic supports reader comprehension and offers a quick reference to the structure and scope of our analysis.

## Results

### Trends and advancements in AD research

In recent years, AI has become an integral part of AD research, contributing to significant advancements across various domains.

**Fig. 4:**
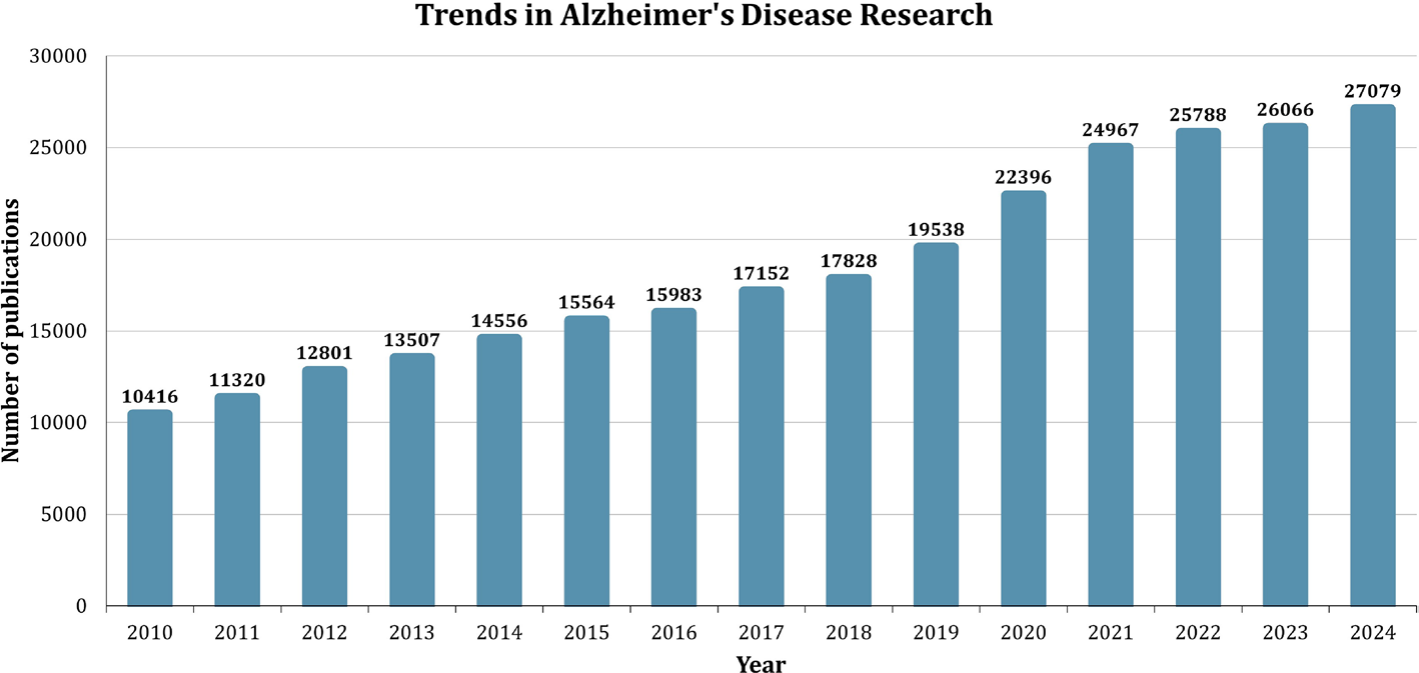
Trends in Alzheimer’s Disease research (2010–2024)

**Fig. 5:**
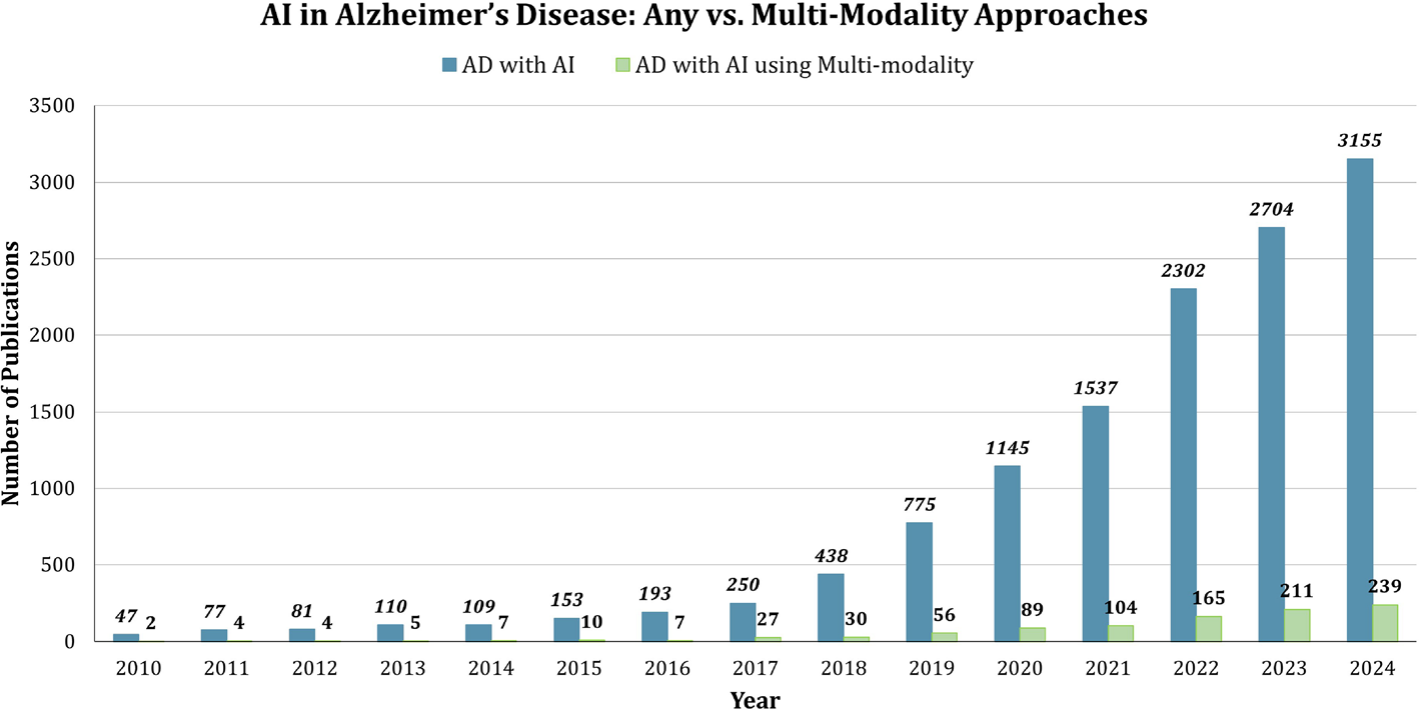
AI in Alzheimer’s disease—any vs. multi-modality approaches (2010–2024)

Figure 4 highlights the overall growth in Alzheimer’s research publications, showcasing a steady increase in output from 10,416 publications in 2010 to an estimated 27,079 in 2024.This trend demonstrates the field’s expanding focus and the urgency of addressing the global burden of Alzheimer’s.

AI’s role in this growth is evident in Fig. 5, which tracks AI adoption in AD research. While early AI applications were mostly limited to single-modality approaches, the data indicates a significant rise in multi-modality methods starting from 2018. By 2024, multi-modality publications reached 239, underscoring the growing preference for integrating diverse data types (e.g., imaging, genetic, and clinical data) to improve accuracy and outcomes in Alzheimer’s research.

**Fig. 6:**
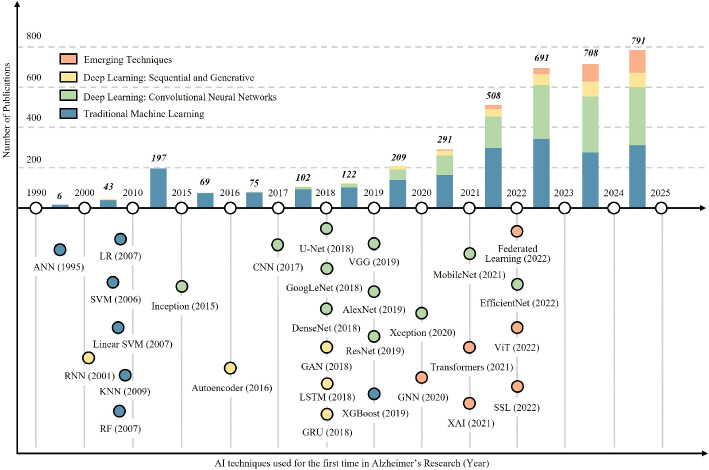
Timeline and publication trends of AI techniques in Alzheimer’s research from 1990 to 2025 based on web of science

The bar chart illustrates the progressive increase in the number of research publications utilizing AI techniques in Alzheimer’s research from 1990 to 2025 in Fig. 6. Initially, the growth was modest, with fewer than 100 publications per year up until 2016. From 2017 onward, the chart shows a rapid rise, reflecting the growing integration of artificial intelligence into biomedical research. Particularly striking is the surge in publications between 2021 and 2025, where the numbers climb from 291 in 2021 to a projected peak of 791 in 2025. The bars are color-coded to represent different categories of AI techniques: traditional machine learning, convolutional neural networks, sequential and generative deep learning, and emerging techniques, indicating not just the volume but the diversity of approaches being adopted in recent years.

The timeline in Fig. 6, below the chart maps out the first use of various AI techniques in Alzheimer’s research, spanning from 1995 to 2022. Each technique is marked by a circle, with its position indicating the year of introduction and its color denoting its category. Traditional machine learning methods such as artificial neural networks (ANN, 1995), support vector machines (SVM, 2006), and random forests (RF, 2007) dominate the early years. From 2015 onward, there is a notable influx of deep learning models: convolutional (e.g., CNN, ResNet, DenseNet) and sequential (e.g., RNN, LSTM, GAN), reflecting advancements in computational power and data availability. Most recently, cutting-edge and emerging methods like transformers (2021), federated learning (2022), and explainable AI (XAI, 2021) signify a shift toward more sophisticated, transparent, and decentralized AI systems, aligning with the increasing demands of precision medicine and ethical AI use.

**Fig. 7:**
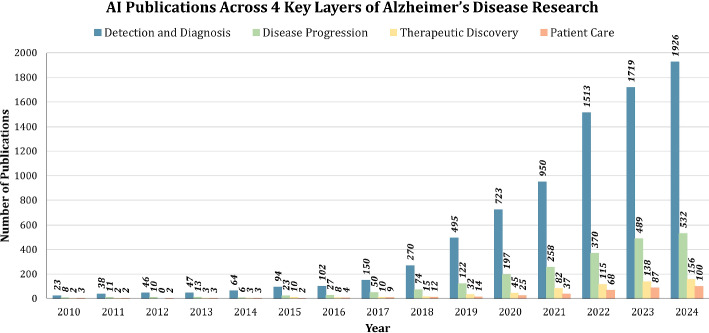
AI Publications Across 4 Key Layers of Alzheimer’s Disease Research (2010–2024) categorizes AI-related publications into four key areas: Detection and Diagnosis, Disease Progression, Therapeutic Discovery, and Patient Care. Detection and Diagnosis dominate the field, reflecting the critical role of AI in early-stage identification of Alzheimer’s. However, areas such as Patient Care remain underrepresented, suggesting potential future research opportunities

Breaking down AI contributions further, Fig. 7 categorizes publications into four proposed major research layers. Detection and Diagnosis dominate the landscape, with AI enabling early identification of Alzheimer’s using innovative techniques such as machine learning and imaging analysis. Disease Progression and Therapeutic Discovery have also gained traction, albeit at a slower pace. Patient Care, the least explored area, presents a significant opportunity for future research, particularly in leveraging AI to improve patient quality of life and caregiving strategies.

**Fig. 8:**
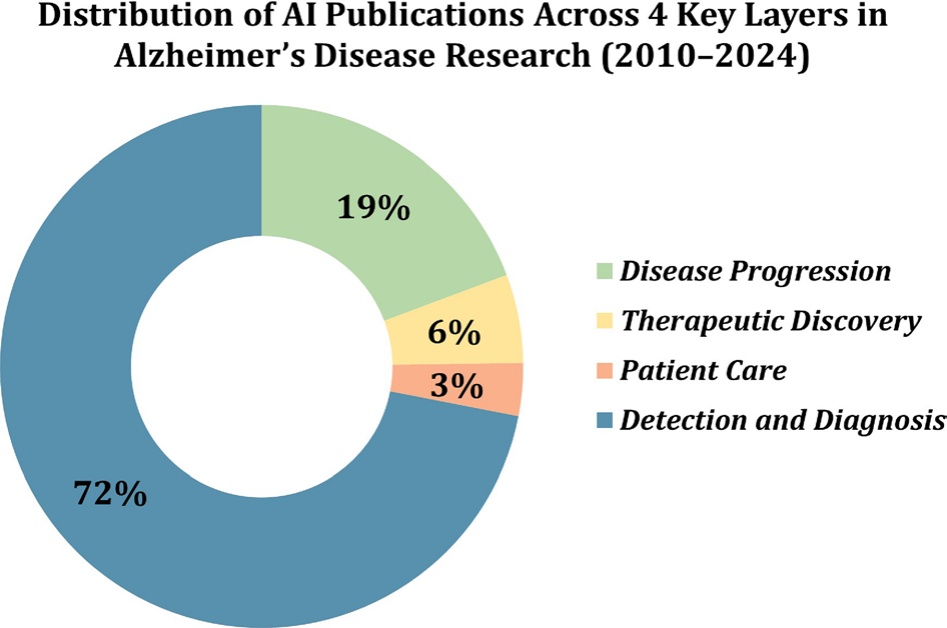
Distribution of AI publications across 4 key layers in Alzheimer’s disease research (2010–2024)

To better understand the emphasis of AI research, Fig. 8 provides a proportional breakdown of publications across these four layers. Detection and Diagnosis account for an overwhelming 71.9% (8,160 out of 11,342) of publications, reflecting AI’s success and prioritization in identifying AD at early stages. Disease Progression follows with 19.3% (2190 out of 11,342), showcasing efforts to model and predict the trajectory of the disease. Therapeutic Discovery (5.5%) and Patient Care (3.3%) represent much smaller portions, indicating gaps in research that need to be addressed. These underexplored domains hold the potential for transformative advancements in treatments and patient-centred applications.

### Proposed layered framework of AI in Alzheimer’s research

We propose a four-layered framework illustrating the integration of artificial intelligence across the Alzheimer’s disease (AD) research continuum. The first layer, Early Detection, focuses on leveraging AI for identifying the stage of Alzheimer’s, disease-specific biomarkers, predicting risk in asymptomatic individuals, and automating cognitive assessments. The second layer, Disease Progression, emphasizes modeling longitudinal data to predict individual trajectories and monitor subtle clinical changes over time. The third layer, Therapeutic Discovery, applies AI to accelerate drug repurposing, identify novel therapeutic targets, and optimize clinical trial design. Finally, the Patient Care layer integrates AI into care delivery through personalized treatment plans, real-time patient monitoring, and enhanced caregiver support systems. This layered approach underscores the potential of AI to contribute holistically to understanding, managing, and ultimately combating Alzheimer’s disease.

To provide readers with a high-level overview of the literature mapped to the Layered Framework, Table 1 summarizes representative studies across each domain. For detailed information on all included studies, readers can refer to the respective detailed tables or subsections for each layer (e.g., Tables 2, 3, 4, 5, 6, 7, 8,9, 10, 11 for imaging based approaches of Early Detection, Table 7 for Disease Progression, Subsection 3.2.3 for Therapeutic Discovery, and Subsection 3.2.4 for real-world integration). This approach allows quick cross-referencing while maintaining readability.

**Table 1:** Summary of representative studies across the layered framework

Layer	Subcategories	Reference to detailed tables
Layer 1: early detection	Imaging	Tables 2, 3, 4, 5, 6, 7, 8,9, 10, 11
	Imaging and clinical	Tables 12, 13
	Imaging and genetic	Table 14
	Imaging, clinical and genetic	Table 15
	Other multimodality	Table 16
Layer 2: disease progression	Imaging	Table 17
	Imaging and clinical	Table 18
	Imaging, clinical and genetic	Table 19
Layer 3: therapeutic discovery	Drug repositioning, therapeutic target identification, drug discovery	Subsection 3.2.3
Layer 4: real-world integration	Remote monitoring, carer support systems	Subsection 3.2.4

#### Layer 1: Early Detection

Early detection of AD is critical for timely intervention and disease management. AI offers powerful tools to identify subtle and early signs of pathology long before clinical symptoms become apparent. By analyzing multimodal data such as neuroimaging, genetic profiles, and electronic health records, AI algorithms can uncover patterns indicative of preclinical or prodromal stages of Alzheimer’s. Machine learning models enhance risk stratification in asymptomatic individuals and support the development of predictive biomarkers. Additionally, AI-powered cognitive assessment tools enable scalable, objective screening, further aiding in the early identification of at-risk populations. These advances position AI as a key enabler in shifting Alzheimer’s diagnosis toward a more proactive and preventative approach.

**Fig. 9:**
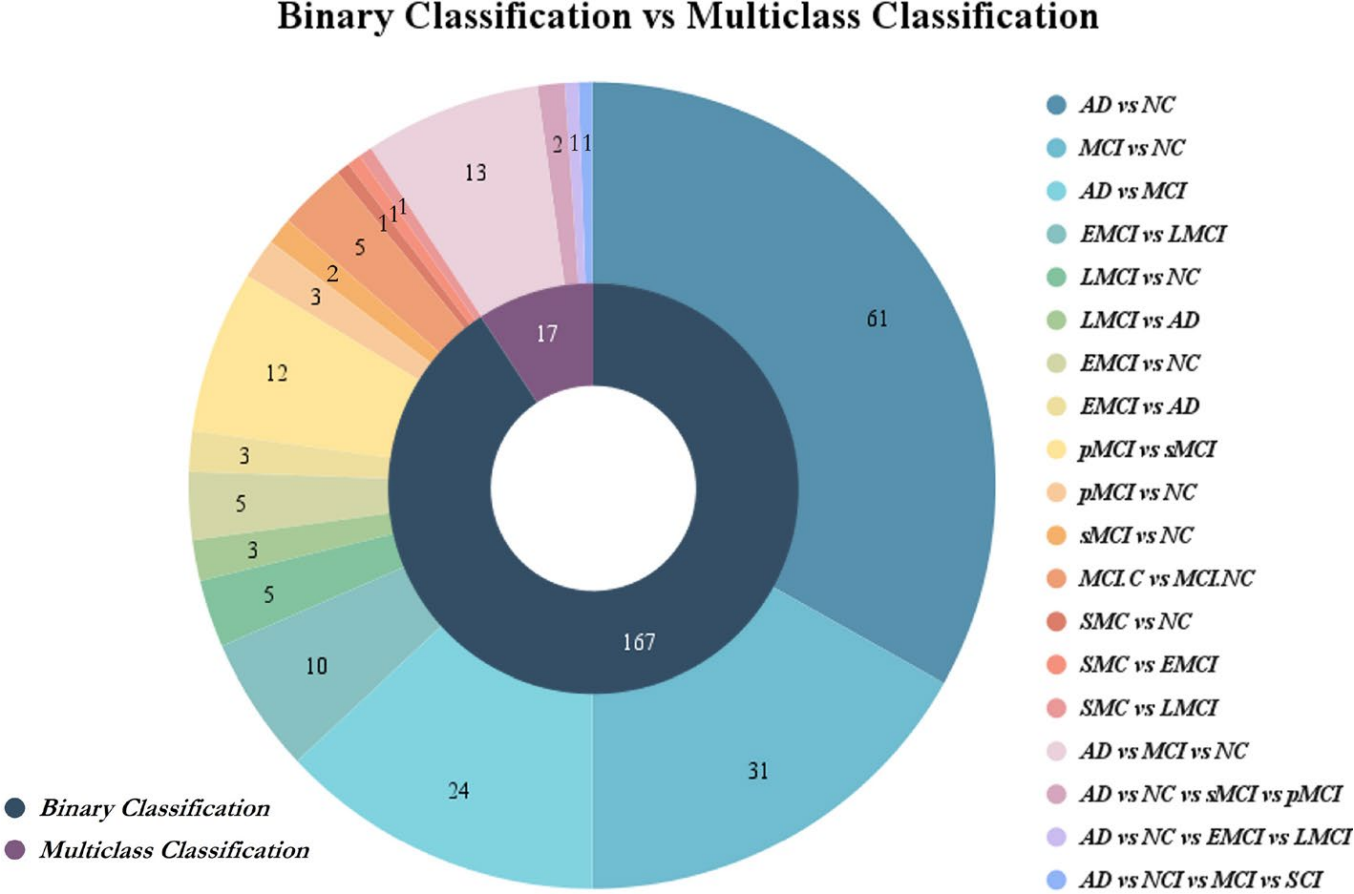
Distribution of AI-based classification strategies in Alzheimer’s disease research. The inner ring represents the overall classification type, with the majority of studies focusing on binary classification (n=167) compared to multiclass classification (n = 17). The outer ring details the specific classification tasks within each category. Common binary tasks include AD vs NC, MCI vs NC, and AD vs MCI, while multiclass tasks involve combinations such as AD vs NC vs MCI or more granular subtypes like EMCI and LMCI. The figure illustrates the field’s current emphasis on binary approaches, despite the growing need for multiclass models in early and differential diagnosis

The predominance of binary classification tasks in Alzheimer’s research highlights the current focus on distinguishing AD from cognitively normal controls (NC), as shown in Fig. 9. In this review of studies applying AI to AD detection, over 90% employed binary models, most commonly AD vs NC, MCI vs NC, and AD vs MCI, due to their relative simplicity and diagnostic clarity. However, real-world clinical scenarios often involve more complex diagnostic categories, such as distinguishing between early and late mild cognitive impairment (EMCI, LMCI) or identifying preclinical stages like subjective cognitive impairment (SCI). Multiclass classification remains underexplored, yet it holds greater potential for reflecting the full spectrum of disease progression and improving early-stage identification. This imbalance underscores the need to advance AI models capable of handling nuanced, multiclass data to better support early and accurate detection of AD.

***Imaging-based Approaches***

AD diagnosis has seen significant advancements with the integration of deep learning and multimodal imaging techniques, particularly through MRI and PET fusion showing in Table 2. Several recent studies have proposed innovative methods to enhance classification accuracy and diagnostic efficiency. A convolutional neural network (CNN)-based approach for AD classification was proposed (Fakoya and Parkinson 2024), integrating MRI and PET imaging through an image fusion technique. By selecting slices from 3D scans and converting them into 2D representations, the method enhances classification efficiency while reducing computational complexity, achieving an accuracy of 94% on the ADNI dataset. Following this, another study (Mahmood et al. 2024) introduced two models: D3LM-LAN, a deep learning framework leveraging ResNet-50 with hybrid attention mechanisms, and MLM-MCSVM, a machine learning model using a multi-core support vector machine (SVM) optimized via Particle Swarm Optimization. These models achieved high classification accuracies, with MLM-MCSVM excelling in early-stage detection. Similarly, a multimodal approach (Maaram and Chandre 2024) integrated MRI and PET imaging with an advanced feature selection and classification framework, employing the Aquila Optimizer-based Arithmetic Optimization Algorithm (AOA) and a Stacked Gated Recurrent Unit (GRU), leading to high classification performance on the ADNI dataset. In another effort, a novel machine learning framework called Same-Subject-Modalities-Interactions (SSMI) (Guelib et al. 2023) was introduced, capturing interactions between different measures from the same brain region within a patient and achieving 98.94% accuracy for AD vs. NC classification. Expanding on multimodal integration, a study (Shukla et al. 2023) proposed an image-fusion method combining PET and MRI with Random Forest-based feature selection and an ensemble classifier, achieving 99% accuracy for binary classifications and 96% for multi-class classification. Likewise, MultiAz-Net (Ismail et al. 2023), an optimized ensemble deep learning model, combined PET and MRI image fusion with the Multi-Objective Grasshopper Optimization Algorithm, achieving 92.3% accuracy for multi-class classification, though computational complexity remained a challenge. A separate study (Yang et al. 2023) introduced a multi-modal fusion framework utilizing spectral graph attention and a bilinear aggregator model, achieving exceptional classification performance with 99.2% AUC for NC vs. AD, 96.1% AUC for MCI vs. AD, and 97.2% AUC for NC vs. MCI. Meanwhile, another approach (Jin et al. 2022) employed a hybrid deep learning model integrating CNN and GAN networks to diagnose mild cognitive impairment (MCI) using multimodal neuroimages, achieving 83.78% accuracy while addressing challenges related to incomplete data. In addition, a relation-induced multi-modal shared representation learning method (Ning et al. 2021) leveraged MRI and PET data to uncover modality associations while mitigating overfitting, achieving high classification accuracy across the ADNI dataset. A different approach (Gao et al. 2022) introduced a deep learning framework combining Task-Induced Pyramid and Attention GAN (TPA-GAN) for imputing missing PET data from MRI, coupled with a Pathwise Transfer Dense Convolution Network (PT-DCN) for multimodal feature integration, reaching 92.7% accuracy for AD vs. NC. Furthermore, an ensemble deep convolutional neural network (DCNN)-based approach (Fang et al. 2020) eliminated the need for manual preprocessing steps, utilizing GoogLeNet, ResNet-50, and DenseNet-121 to achieve high classification performance, with a peak accuracy of 99.27% for AD vs. NC. Another study (Li et al. 2015) presented a deep learning framework incorporating techniques such as PCA, stability selection, dropout, and multitask learning, resulting in notable classification accuracy improvements over traditional methods, with a 5.9% increase on the ADNI dataset.

**Table 2:** Detailed review of multimodal imaging-based (MRI+PET) approaches

Study	Modalities	AI techniques	Datasets	Metric	Key strengths	Limitations
Fakoya and Parkinson (2024)	Imaging (MRI + PET)	Image fusion + CNN	ADNI	ACC – 94%	Data fusion method	Limited data augmentation
Mahmood et al. (2024)	Imaging (MRI + PET)	CNN + Multicore SVM	ADNI	AD vs. NC: ACC 98.59%; MCI vs. NC: ACC 96.53%; AD vs. MCI: ACC 95.77%; EMCI vs. LMCI: ACC 96.32%	Multi-core SVM with bilinear multi-channel nets replacing softmax; richer decision boundaries	Potential to improve segmentation accuracy and reduce image redundancy (not yet shown)
Maaram and Chandre (2024)	Imaging (MRI + PET)	Aquila Optimizer–based AOA + Stacked GRU (Leaky ReLU)	ADNI	AD vs. NC: ACC 97.96%; MCI vs. NC: ACC 96.57%; AD vs. MCI: ACC 95.61%	Feature selection to remove irrelevant features from full subset	(not reported)
Guelib et al. (2023)	Imaging (MRI + PET)	SSMI	ADNI	AD vs. NC: ACC 98.94%	Captures interactions between regional measures for the same subject	Manual parameter selection; small, mildly imbalanced dataset
Shukla et al. (2023)	Imaging (MRI + PET)	Ensemble model	ADNI	ACC 96%	(not reported)	(not reported)
Ismail et al. (2023)	Imaging (MRI + PET)	CNN (Ensemble)	ADNI	ACC (92.3 ± 5.45)%	Combines anatomical + metabolic brain information	Computationally heavy; storage intensive; limited (adolescent/young adult cohort)
Yang et al. (2023)	Imaging (MRI + PET)	Spectral graph attention net + bilinear aggregator	Cora; Citeseer; PubMed; TADPOLE	NC vs. AD: AUC 99.2%; MCI vs. AD: AUC 96.1%; NC vs. MCI: AUC 97.2%	(not reported)	(not reported)
Jin et al. (2022)	Imaging (MRI + PET)	CNN + GAN	ADNI (1, 2, GO)	ACC 83.78%; SEN 80.95%; SPE 87.5%; AUC 88.99%	Handles incomplete multimodal data	Not optimized for other datasets
Ning et al. (2021)	Imaging (MRI + PET)	Relation-induced multimodal shared rep. learning	ADNI (1,2)	ADNI1: AD vs. NC ACC 96.9%; MCI vs. NC ACC 82.6%; pMCI vs. sMCI ACC 84.5%. ADNI2: AD vs. NC ACC 96.8%; MCI vs. NC ACC 81.5%; pMCI vs. sMCI ACC 85.9%	Finds latent cross-modal relationships; reduces overfitting	(not reported)
Gao et al. (2022)	Imaging (MRI + PET)	TPA-GAN + PT-DCN	ADNI	AD vs. NC ACC 92.7%; pMCI vs. sMCI ACC 75.3%	TPA-GAN generates PET detail while preserving task-relevant info	Train: ADNI-1; Test: ADNI-2 (domain differences ignored)
Fang et al. (2020)	Imaging (MRI + PET)	DCNN (GoogLeNet, ResNet-50, DenseNet-121)	ADNI	AD vs. NC ACC 99.27%; MCI vs. NC ACC 90.35%; AD vs. MCI ACC 92.57%	Avoids registration/segmentation; ROI-free (no handcrafted patches needed)	(not reported)
Li et al. (2015)	Imaging (MRI + PET)	PCA; stability selection; dropout; multi-task deep learning	ADNI	AD vs. HC 91.4%; MCI vs. HC 77.4%; AD vs. MCI 70.1%; MCI.C vs. MCI.NC 57.4%	Dropout helped mitigate overfitting	(not reported)

**Table 3:** Detailed review of multimodal imaging-based (MRI+FDG-PET)approaches

Study	Modalities	AI techniques	Datasets	Metric	Key strengths	Limitations
Song et al. (2021)	Imaging (MRI + FDG–PET)	3D Simple CNN + 3D Multi-Scale CNN	ADNI	AD vs. NC ACC 94.11%; MCI vs. NC ACC 88.48%; AD vs. MCI ACC 84.83%; 3-class AD/MCI/NC ACC 74.54%	Image-fusion approach reduces CNN parameter count; outperforms feature-level fusion	Not robust across datasets
Shao et al. (2020)	Imaging (MRI + FDG–PET)	Hypergraph multi-task feature selection + multi-kernel SVM	ADNI	AD vs. NC ACC 92.51%; LMCI vs. NC ACC 82.53%; EMCI vs. LMCI ACC 75.48%	Captures high-order relationships within each modality via hypergraph structure	Uses fixed hyperedge weights (no adaptive weighting)
Zu et al. (2016)	Imaging (MRI + FDG–PET)	Label-aligned multi-task feature learning	ADNI	AD vs. NC ACC 95.95%; MCI vs. NC ACC 80.26%; MCI-C vs. MCI-NC ACC 69.78%	Leverages cross-modality + cross-subject relationships	Binary focus; equal feature counts required across modalities; baseline-only data

**Table 4:** Detailed review of multimodal imaging-based (T1-MRI+FDG-PET)approaches

Study	Modalities	AI techniques	Datasets	Metric	Key strengths	Limitations
Tang et al. (2024)	Imaging (T1 MRI + FDG–PET)	Multiscale attention and cross-enhancement fusion network (MACFNet)	ADNI (1, 2, GO & 3)	AD vs. NC ACC 99.91%; MCI vs. AD ACC 99.89%; CN vs. MCI ACC 99.63%; 3-class CN/MCI/AD ACC 97.75%	Suppresses redundant multi-scale information to reduce negative impacts during fusion	Ignores features in ROI regions
Dwivedi et al. (2022)	Imaging (T1 MRI + FDG–PET)	DWT + RELS-TSVM + CNN	ADNI	AD vs. NC ACC 97%; CN vs. MCI ACC 94%; MCI vs. AD ACC 97.5%	Key slices selected via GLCM features reduce computation cost	Uncertainty in model parameters due to RELS-TSVM classifier
Zhang and Shi (2020)	Imaging (T1 MRI + FDG–PET)	Multi-modal fusion network (attention-based)	ADNI	AD vs. NC ACC 95.21%; sMCI vs. pMCI ACC 89.79%; 4-class ACC 86.12%	Hierarchical fusion learns synergy between multi-modal data	Cannot handle missing data
Huang et al. (2019)	Imaging (T1 MRI + FDG–PET)	CNN	ADNI	AD vs. CN ACC 90.10%; CN vs. pMCI ACC 87.46%; pMCI vs. sMCI ACC 76.9%	Segmentation unnecessary; only hippocampal ROIs used	CNN-extracted features are difficult to interpret
Liu et al. (2018)	Imaging (T1 MRI + FDG–PET)	Cascaded CNN + 3D CNN + 2D CNN	ADNI	AD vs. NC ACC 93.26%; NC vs. pMCI ACC 82.95%; sMCI vs. NC ACC 64.04%	No segmentation or rigid registration required	Determining optimal layer count and kernel size is challenging

**Table 5:** Detailed review of multimodal imaging-based (T1-MRI/T2-MRI+PET) approaches

Study	Modalities	AI techniques	Datasets	Metric	Key strengths	Limitations
Khan et al. (2024)	Imaging (T1 MRI + PET)	Multi-scale feature extraction with mixed-transformer + enhanced U-Net (semantic segmentation)	ADNI	AD vs. NC ACC 98%; MCI vs. NC ACC 97.9%; AD vs. MCI ACC 98.2%	Reduces complexity; captures multi-level granularity; prevents overfitting	Requires high-quality, diverse data; high computational demand; low interpretability
Ahmed et al. (2023)	Imaging (T1 MRI + PET)	Laplacian Re-Decomposition + VGG16-XGBoost	ADNI	4-class (AD/NC/EMCI/LMCI): Early fusion ACC 98.06%; Late fusion ACC 99.22%	Mitigates overfitting	High labeling effort; difficult bias level selection
Goenka and Tiwari (2023)	Imaging (T1w-MRI + AV-45 PET)	Ensembled volumetric ConvNet	ADNI	AD vs. NC vs. MCI ACC 93.01%; NC vs. MCI ACC 96.14%; MCI vs. AD ACC 98.88%; AD vs. NC ACC 97.26%	Multi-class volumetric ConvNet improves early screening	Not reported
Dai et al. (2021)	Imaging (T2 MRI + PET)	Wavelet fusion algorithm + CNN	ADNI	ACC 90.9%; AUC 91.6%	Introduced fusion imaging for Alzheimer’s disease	Small training dataset

**Table 6:** Detailed review of multimodal imaging-based (sMRI+PET)approaches

Study	Modalities	AI techniques	Datasets	Metric	Key strengths	Limitations
Liu et al. (2024a)	Imaging (sMRI + PET)	Multi-Task Joint Learning Network (MTJLN)	ADNI	AD vs. MCI vs. NC ACC 78%	Introduces image patch pruning (IPP) strategy	Unstructured pruning fails to reduce computation/parameters; future clinical scores unpredictable; missing data issue unresolved
Zhang et al. (2023c)	Imaging (sMRI + PET)	3D CNN + 3D attention mechanisms + multi-layer feature fusion	ADNI	AD vs. NC ACC 94.61%; sMCI vs. pMCI ACC 77.19%	Not reported	Not reported

**Table 7:** Detailed review of multimodal imaging-based (sMRI+FDG-PET+florbetapir-PET/AV45-PET) approaches

Study	Modalities	AI techniques	Datasets	Metric	Key strengths	Limitations
Leng et al. (2023)	Imaging (sMRI + FDG–PET)	Patch-based efficient 3D Multimodal cross Enhanced fusion Network (MENet) + lightweight multimodal network	ADNI	AD vs. NC ACC 97.67%; sMCI vs. CN ACC 81.63%	Requires no predefined landmarks or additional location modules (e.g., hippocampus segmentation)	Use of a single template leads to potential bias
Zhang et al. (2023b)	Imaging (sMRI + FDG–PET)	Multi-modal GNN	ADNI (1,2)	AD vs. NC ACC 96.68%; sMCI vs. pMCI ACC 78.00%	Combines multi-modal imaging information at both population level and individual adjacency matrices	Further validation needed with additional datasets
Lao and Zhang (2022)	Imaging (sMRI + FDG–PET)	3D discrete wavelet transform (3D-DWT) + 3D moment invariants (3D-MIs)	ADNI	AD vs. CN ACC 96.92%; AD vs. MCI ACC 84.29%; pMCI vs. sMCI ACC 87.78%	Not reported	Subjects with only MRI or PET images were excluded
Li et al. (2017)	Imaging (sMRI + FDG–PET + florbetapir-PET)	Multi-modal supervised within-class-similarity discriminative dictionary learning algorithm (SCDDL)	ADNI (1,2 & GO)	AD vs. NC ACC 98.5%; CN vs. MCI ACC 82.8%	Not reported	Only weighted combination method used for multi-modality analysis; cognition information not included
Zhang et al. (2023)	Imaging (sMRI + AV45–PET + FDG–PET)	3D multi-layer perceptron mixer model (3D-Mixer)	ADNI + NACC + AIBL + OASIS + MIRIAD	ADNI: sMCI vs. pMCI ACC 75.5%; AIBL: CN vs. AD ACC 93.4%	First work employing MLP-Mixer architecture for PET data prediction	Interaction between modalities not fully considered; sMRI heterogeneity from different datasets not addressed

**Table 8:** Detailed review of multimodal imaging-based (VBM-MRI/tauPET/Aβ PET) approaches

Study	Modalities	AI techniques	Datasets	Metric	Key strengths	Limitations
Castellano et al. (2024)	Imaging (2D & 3D MRI + PET)	CNN	OASIS-3	ACC 95%, SEN 93.33%, SPE 96.66%, AUC 93%	Not reported	Selected only 50 slices from the axial plane for both PET and MRI analyses, limiting depth of predictions and interpretations
Kim et al. (2024)	Imaging (T1 MRI + FDG–PET + A$\beta$ PET + tau PET)	Middle-fusion multimodal model	ADNI	AD vs. NC ACC 100%; MCI vs. CN ACC 76%	Novel region-of-interest (ROI) extraction method	Not reported
Ye et al. (2023)	Imaging (VBM–MRI + FDG–PET + AV45–PET)	Pairwise feature-based generative adversarial network (GAN)	ADNI	50% missing rate VBM/FDG: AD vs. NC ACC 91.37%; EMCI vs. LMCI ACC 85.11% 50% missing rate VBM/AV45: AD vs. NC ACC 90.83%; EMCI vs. LMCI ACC 84.22%	Handles missing whole modalities, not just random missing values	Not reported
Hao et al. (2020)	Imaging (VBM–MRI + FDG–PET)	Multi-modal neuroimaging feature selection with consistent metric constraint (MFCC) + multi-kernel SVM	ADNI (1,2)	ADNI1: AD vs. NC ACC 97.60%; MCI vs. NC ACC 84.47%; MCI-C vs. MCI-NC ACC 77.76% ADNI2: AD vs. NC ACC 93.72%; MCI vs. NC ACC 78.47%; MCI-C vs. MCI-NC ACC 73.87%	Takes full advantage of similarity relationships between samples	Focuses only on binary classification; feature representations generated from a single template may be insufficient

**Table 9:** Detailed review of multimodal imaging-based (fMRI/rs-fMRI)approaches

Study	Modalities	AI techniques	Datasets	Metric	Key strengths	Limitations
Ramani et al. (2024)	Imaging (sMRI + fMRI)	3D-CNN + RNN	ADNI	NCI, SCI, MCI, and AD: ACC 99.5%	Captures anatomical and functional changes	Not reported
Long et al. (2023)	Imaging (sMRI + rs-fMRI)	DPARSF + VBM (SPM8) + SVM + ANN	AD & MCI: Nanfang Hospital; HC: local community	AD vs. MCI vs. NC: ACC 80.36%	Multi-class classification	Other brain atlases could be utilized; small dataset
Hu et al. (2022)	Imaging (rs-fMRI + sMRI)	Deep learning-based Granger causality estimator (RNN-GC) + SVM	MR Research Center, Wenzhou Medical University	AD vs. NC: ACC 91.49%	Considers changing propagation delay between brain signals for network construction	Threshold selection is controversial; no other modalities (e.g., PET, EEG, CSF)
Liu et al. (2020)	Imaging (T1 MRI + rs-fMRI)	Graph convolutional networks (GCNs)	ADNI	EMCI vs. NC: ACC 84.1%, AUC 85.6%	Not reported	Not reported

**Table 10:** Detailed review of multimodal imaging-based (DTI) approaches

Study	Modalities	AI techniques	Datasets	Metric	Key strengths	Limitations
Li et al. (2024)	Imaging (sMRI + DTI)	Residual convolutional neural network (MADNet)	ADNI + XWNI (Xuanwu Hospital Neuroimaging)	(not reported)	(not reported)	Limited data size; trained from scratch
Houria et al. (2023)	Imaging (sMRI + DTI)	Fused BoF + SURF + modified AlexNet (CNN) + ensemble SVM	ADNI	AD vs. MCI vs. NC: ACC 98.42%	Uses local and deep features from DTI-FA, DTI-MD, and GM images, classified via cubic SVM	Other stages of impairment need inclusion; reslicing 3D to 2D affects temporal dependencies; majority voting ignores label confidence
Aderghal et al. (2020)	Imaging (sMRI + DTI)	Transfer learning CNNs	ADNI	AD vs. NC: ACC 92.30%, MCI vs. AD: ACC 79.16%	Intelligent initialization of network parameters	Needs improvement with additional ROIs
Fang et al. (2022b)	Imaging (MRI + DTI)	Re-transfer learning + multi-modal learning + CNN4D deep neural network	ADNI	AD vs. NC: ACC 94.6%, NC vs. EMCI: 93.5%, CN vs. LMCI: 90.9%, EMCI vs. LMCI: 80.8%, EMCI vs. AD: 92.5%, LMCI vs. AD: 92.6%	Fine-grained brain image classification	(not reported)
Meng and Zhang (2023)	Imaging (fMRI + DTI)	Dual Fusion Cluster Graph Convolution Network (DFC GCN)	ADNI	NC vs. MCI: ACC 90.70%	Explores brain connectivity while avoiding complex network construction	(not reported)
Meng et al. (2022)	Imaging (fMRI + DTI)	Multi-modal LassoNet	ADNI	AD vs. NC: ACC 90.68 ± 0.34%, AD vs. EMCI: 83.63 ± 0.74%, EMCI vs. NC: 88.77 ± 0.70%	Fully reflects dynamic mechanisms of brain network connections	May have biases in fiber configuration selection
Wee et al. (2012)	Imaging (DTI + rs-fMRI)	Multiple-kernel SVM	ADNI	MCI detection: ACC 96.3%	Combines anatomical and functional connectivity information	Unbalanced data; lack of standardized parcellation approaches
Tian et al. (2023)	Imaging (T1 MRI + DTI + fMRI)	Hierarchical graph convolutional network (EH-GCN)	ADNI + PUTH (Peking University Third Hospital)	AD vs. NC: 88.71%, MCI vs. NC: 79.68%, AD vs. MCI: 82.71%	Extensible and adjustable learning framework	Influence of ROI selection not discussed; not end-to-end framework

**Table 11:** Detailed review of multimodal imaging-based (Diffusion MRI) approaches

Study	Modalities	AI techniques	Datasets	Metric	Key strengths	Limitations
Kaka and Satya Prasad (2022)	Imaging (fMRI + PET)	Differential Evolution-Multiclass Support Vector Machine (DE-MSVM)	ADNI	ACC - 98.13%	Mitigates overfitting and local optima	Not reported
Wen et al. (2021)	Imaging (T1 MRI + Diffusion MRI)	SVM	ADNI	AD vs. NC: BA - 94%, NC vs. MCI: BA - 74%, pMCI vs. sMCI: BA - 80%	Proposed a standard diffusion MRI processing pipeline	ADNI diffusion MRI data not acquired using state-of-the-art methods
Lei et al. (2020)	Imaging (rsMRI + DTI)	Low-rank self-calibrated functional and structural brain networks + joint multi-task learning + SVM	ADNI	SMC vs. NC: 82.95%, NC vs. EMCI: 85.23%, NC vs. LMCI: 87.80%, SMC vs. EMCI: 84.09%, SMC vs. LMCI: 90.24%, EMCI vs. LMCI: 81.71%	Not reported	Structural and functional brain networks constructed separately; functional connection analysis based on single-subject approach

Despite reported accuracies ranging from 83.7% to 99.2% across MRI-PET fusion models, current approaches face key limitations. Over 90% of studies rely solely on the ADNI dataset, raising concerns about overfitting and generalizability. Evaluation is often limited to accuracy, with few reporting AUC, F1-score, or cross-validation robustness, particularly critical in imbalanced datasets like ADNI, where MCI cases are under-represented. Computationally intensive models (e.g., MultiAz-Net, TPA-GAN) hinder real-time or low-resource deployment. Fusion strategies are inconsistently defined, few distinguish early (feature-level), intermediate, or late (decision-level) fusion, limiting reproducibility. Moreover, explainability is rarely addressed, with less than 10% of reviewed works incorporating model interpretation tools (e.g., Grad-CAM, SHAP), undermining clinical transparency and trust.

In the context of MRI and FDG-PET integration for AD diagnosis shown in Table 3, several studies have explored novel fusion and feature selection techniques to enhance classification accuracy. One study (Song et al. 2021) proposed an image fusion method that combines gray matter (GM) regions from MRI with FDG-PET to create a “GM-PET” modality, improving interpretability in AD diagnosis. By employing 3D Simple CNN and 3D Multi-Scale CNN, the approach reduces CNN parameters while outperforming unimodal and traditional fusion methods, achieving 94.11% accuracy for AD vs. NC and 74.54% for multi-class classification on the ADNI dataset. Another study (Shao et al. 2020) introduced a hypergraph-based multi-task feature selection method that leverages high-order structural relationships between MRI and FDG-PET to improve classification. Using a multi-kernel support vector machine (SVM), the method attained high accuracy, with 92.51% for AD vs. NC and 75.48% for EMCI vs. LMCI, though it relies on fixed hyperedge weights. Additionally, a multimodal classification approach (Zu et al. 2016) explored relationships across MRI and FDG-PET imaging modalities, integrating label-aligned multi-task feature selection with a multi-kernel SVM. Evaluated on the ADNI dataset, the method demonstrated strong performance with 95.95% accuracy for AD vs. NC, though its reliance on binary classification and the need for matched feature numbers across modalities present certain limitations.

For the integration of T1 MRI and FDG-PET in AD diagnosis, several advanced multimodal fusion techniques have been proposed shown in Table 4. One study (Tang et al. 2024) introduced MACFNet, a diagnostic framework utilizing a cross-enhanced fusion mechanism and multiscale attention to suppress redundant information before fusion, achieving exceptional classification accuracies of up to 99.91% for AD vs. NC on the ADNI dataset. However, its limitation lies in neglecting features from regions of interest (ROI), which may affect its clinical applicability. Another study (Dwivedi et al. 2022) proposed a multimodal fusion method that employs the demon algorithm and discrete wavelet transform (DWT) for feature extraction, alongside ResNet-50 and a robust energy least square twin support vector machine (RELS-TSVM) for classification. The approach demonstrated high accuracy, such as 97% for CN vs. AD, but faces uncertainties in RELS-TSVM parameter selection. A hierarchical fusion network (Zhang and Shi 2020) was also introduced, leveraging attention mechanisms to extract relevant features and suppress irrelevant ones, achieving 95.21% accuracy for AD vs. NC. While it enhances feature synergy between modalities, it cannot handle missing data. Another approach (Huang et al., 2019) utilized a 3D CNN to integrate T1-MRI and FDG-PET hippocampal imaging, eliminating manual feature extraction and achieving strong classification performance, including 90.10% for AD vs. CN. However, it is restricted to hippocampal ROIs, limiting interpretability. Finally, a cascaded CNN framework (Liu et al. 2018) was proposed to automatically extract multi-level features from MRI and PET images, attaining 93.26% accuracy for AD vs. NC. While it streamlines the classification process by removing the need for segmentation and rigid registration, optimal parameter selection for layers and kernels remains a challenge.

Integrating T1-weighted MRI and PET imaging in AD diagnosis, several advanced multimodal fusion techniques have been proposed shown in Table 5. One study (Khan et al. 2024) introduced Dual-3DM3-AD, a deep learning approach that leverages a Mixed-Transformer with Furthered U-Net for segmentation and Multi-Scale Feature Extraction, achieving classification accuracies of up to 98.2% for AD vs. MCI. However, its reliance on high-quality datasets and computational demands presents challenges for broader clinical applications. Another study (Ahmed et al. 2023) proposed a dual-stage fusion approach, integrating Laplacian Re-Decomposition for early fusion and Canonical Correlation Analysis for late fusion, using CNNs and XGBoost for classification. This method achieved a remarkable 99.22% accuracy for late fusion but faces challenges in labeled data production and bias selection. Additionally, an ensembled volumetric ConvNet model (Goenka and Tiwari 2023) was introduced, combining T1w-MRI and AV-45 PET scans to classify AD, NC, and MCI with a high AUC of 93.01%, surpassing traditional patch- and slice-based methods. Lastly, a deep learning and radiomics-based approach (Dai et al. 2021) implemented a wavelet fusion algorithm for T2 MRI and PET imaging, achieving 90.9% accuracy and an AUC of 91.6%. While demonstrating higher diagnostic accuracy than single-modality approaches, the method’s limited training sample size raises concerns about its generalizability.

Recent advancements in deep learning frameworks have led to the integration of structural MRI (sMRI) and PET imaging for AD diagnosis shown in Table 6. One study (Liu et al. 2024a) introduced the Multi-Task Joint Learning Network (MTJLN), which integrates fine-grained image features with non-image clinical data, employing an Image Patch Pruning (IPP) strategy and a weighted loss function to handle incomplete clinical scores. The model achieved 78% accuracy for AD, MCI, and NC classification but has limitations in structured pruning and predicting future clinical scores. Another study (Zhang et al. 2023c) proposed an end-to-end multimodal framework utilizing 3D CNNs, attention mechanisms, and spatial pyramid pooling to extract meaningful features from sMRI and PET data. Achieving 94.61% accuracy for AD vs. NC classification and 77.19% for sMCI vs. pMCI, this model outperformed single-modality approaches and state-of-the-art multimodal techniques, demonstrating its potential for accurate and early AD diagnosis.

Recent advances in integrating structural MRI (sMRI) and PET imaging for AD diagnosis have introduced several innovative models shown in Table 7. One study (Leng et al. 2023) presented MENet, a lightweight multimodal network designed for AD and subjective memory complaint (SMC) diagnosis, achieving 97.67% accuracy for AD classification and 81.63% for SMC classification on the ADNI dataset. MENet’s strength lies in its ability to function without predefined landmarks like hippocampus segmentation, although its reliance on a single template may introduce bias. Another study (Zhang et al. 2023b) proposed a multi-modal graph neural network (GNN) framework that integrates brain networks from sMRI, FDG-PET, and phenotypic data, achieving 96.68% accuracy for AD vs. NC classification. This approach outperformed existing methods, though further validation across datasets is required to confirm its generalizability. A different study (Lao and Zhang 2022) introduced a method combining sMRI and FDG-PET images with 3D discrete wavelet transform (3D-DWT) and deep neural networks, achieving 96.92% accuracy for AD vs. CN classification and demonstrating the model’s potential for distinguishing progressive and stable MCI. While the study excluded subjects with only MRI or PET images, it highlighted the benefits of multimodal fusion. Another study (Li et al. 2017) introduced a multi-modal supervised within-class-similarity discriminative dictionary learning algorithm (mSCDDL) for AD and MCI classification, achieving 98.5% accuracy for AD vs. NC. However, it lacked the incorporation of cognitive information, which could improve diagnostic precision. Lastly, a study (Zhang et al. 2023) presented the 3D multi-layer perceptron mixer model (3D-Mixer), which predicts FDG-PET and AV45-PET SUVRs from structural MRI. Despite demonstrating high accuracy across multiple datasets, including ADNI and AIBL, its limitations include not fully accounting for interactions between modalities and variability in sMRI data across datasets.

Recent studies have advanced the integration of multimodal data for AD diagnosis by combining structural MRI, PET imaging, and deep learning methods shown in Table 8. One study (Castellano et al. 2024) proposed a deep learning approach that integrated 2D and 3D MRI with amyloid PET imaging using a convolutional neural network (CNN), achieving 95% accuracy on the OASIS-3 dataset. This model demonstrated high sensitivity and specificity, but its reliance on only 50 axial slices may limit its depth of predictions and generalizability. Another study (Kim et al. 2024) introduced a middle-fusion multimodal model using T1 MRI, FDG PET, A$\beta$ PET, and tau protein PET data, achieving 100% accuracy for AD vs. NC classification. This approach demonstrated great promise for early AD detection, but further validation is required for broader application. A third study (Ye et al. 2023) utilized a pairwise feature-based generative adversarial network (GAN) to address missing modalities in AD diagnosis, achieving promising results with 91.37% accuracy when 50% of the VBM/FDG data was missing. This model showcased the potential of GANs in enhancing diagnostic robustness, even with incomplete data. Additionally, a method (Hao et al. 2020) for multi-modal neuroimaging feature selection with consistent metric constraints achieved 97.60% accuracy for AD vs. NC classification, outpacing traditional methods on both ADNI1 and ADNI2 datasets. However, the approach’s limitations include its focus on two-category classification and reliance on a single template for feature representation. Collectively, these studies highlight the potential of multimodal frameworks, GANs, and feature selection methods in addressing key challenges in AD diagnosis, such as missing data and early-stage detection.

Recent studies have explored advanced hybrid and multimodal deep learning frameworks to improve early Alzheimer’s disease (AD) diagnosis by integrating various neuroimaging modalities shown in Table 9. One study (Ramani et al. 2024) proposed a hybrid deep learning model that combined 3D convolutional neural networks (3D-CNNs), recurrent neural networks (RNNs), and transfer learning using structural MRI (sMRI) and functional MRI (fMRI) data, achieving an impressive accuracy of 99.5% across multiple diagnostic categories on the ADNI dataset. This approach showcases the power of combining anatomical and functional information for enhanced diagnostic precision. Another study (Long et al. 2023) combined resting-state fMRI and structural MRI metrics with a machine learning approach, achieving an accuracy of 80.36% for classifying AD, MCI, and healthy controls. Despite its potential, the study faced limitations like a small dataset and the need for improved brain atlases. A third study (Hu et al. 2022) introduced a deep learning-based Granger causality estimator (RNN-GC), integrated with SVM, for constructing brain networks using rs-fMRI and sMRI data, achieving a high accuracy of 91.49%. This method, while demonstrating improved diagnostic performance, faced challenges related to threshold selection and the absence of additional modalities such as PET or EEG. Lastly, a study (Liu et al. 2020) presented a graph convolutional network (GCN)-based framework for early identification of mild cognitive impairment (EMCI), which achieved an accuracy of 84.1% and an AUC of 85.6%. This approach demonstrated the potential of multimodal data integration in identifying early-stage AD and EMCI. These studies highlight the ongoing advancements in multimodal frameworks and deep learning models, offering significant promise for improving early diagnosis and clinical applications of AD.

From Table 10, a recent study (Li et al. 2024) introduced MADNet, a multimodal deep learning model for AD diagnosis, integrating structural MRI (sMRI) and diffusion tensor imaging (DTI) data. The model employs attention-based feature fusion with ResNet-10 as its backbone, enhancing classification performance and interpretability. Evaluated on the ADNI and XWNI (Xuanwu Hospital Neuroimaging) datasets, MADNet outperforms single-modality models, demonstrating the advantages of multimodal integration. Grad-CAM visualizations further highlight the model’s focus on critical brain regions relevant to AD pathology. However, the study is limited by its relatively small dataset size and the need for training from scratch, which may affect generalizability to larger, more diverse populations. Another study (Houria et al. 2023) presented a multimodal approach for early Alzheimer’s disease detection by combining diffusion tensor imaging (DTI) and structural MRI (sMRI) data. The method uses fused Bag-of-Features (BoF) and modified AlexNet for feature extraction, achieving 98.42% accuracy in classifying AD, MCI, and cognitively normal (CN) subjects. However, the study notes limitations such as reslicing 3D volumes into 2D slices, which impacts temporal dependencies, and the use of majority voting based solely on final prediction labels, neglecting the confidence factor of each label. Further research could address additional stages of cognitive impairment for more comprehensive detection. A different study (Aderghal et al. 2020) proposed a transfer learning-based CNN framework using sMRI and DTI, focusing on the hippocampal region. The model achieved 92.30% accuracy for AD vs. NC classification and 79.16% for MCI vs. AD classification. Despite working with small datasets and low-resolution scans, the method demonstrated significant improvements in classification accuracy, with potential for further enhancement by incorporating additional regions of interest (ROIs). Another study (Fang et al. 2022b) introduced a fine-grained classification method for Alzheimer’s diagnosis, utilizing a CNN-based model (CNN4AD) with a re-transfer learning approach to handle multi-modal MRI and DTI data. This method achieved high accuracy in distinguishing between various stages of cognitive decline on the ADNI dataset, including 94.6% accuracy for distinguishing AD vs. NC, 93.5% for NC vs. EMCI, and 90.9% for CN vs. LMCI. It also demonstrated strong performance in differentiating between EMCI vs. LMCI (80.8%), EMCI vs. AD (92.5%), and LMCI vs. AD (92.6%), showcasing its potential for early detection of AD and Mild Cognitive Impairment (MCI). A recent study (Meng and Zhang 2023) proposed the Dual Fusion Cluster Graph Convolution Network (DFCGCN) for diagnosing Mild Cognitive Impairment (MCI) using multi-modal data, combining fMRI and DTI. Tested on the ADNI dataset, the model achieved 90.7% accuracy, 91.1% sensitivity, and 94.0% specificity, surpassing existing methods for early MCI detection. The DFCGCN model thoroughly explores brain connectivity characteristics, avoiding the complex construction of brain connection networks, and shows promise for enhancing diagnostic accuracy and sensitivity. Another study (Meng et al. 2022) introduced the multi-modal LassoNet framework, which integrates resting-state fMRI (rs-fMRI) and diffusion tensor imaging (DTI) for AD classification and feature detection. It achieved high performance, with accuracies of 90.68% for AD vs. NC, 83.63% for AD vs. EMCI, and 88.77% for EMCI vs. NC, and offers insights into the dynamic brain network connections underlying AD. However, it may introduce biases in determining the most optimal fiber configuration. A separate study (Wee et al. 2012) combining DTI and rs-fMRI for early MCI detection using a multiple-kernel support vector machine (SVM) achieved an impressive 96.3% classification accuracy, outperforming single-modality approaches. Despite challenges like unbalanced data and the lack of standardized brain parcellation techniques, this study demonstrates the potential of multimodal imaging for early MCI detection. Lastly, a study (Tian et al. 2023) introduced the Extensible Hierarchical Graph Convolutional Network (EH-GCN), a model for early AD diagnosis that integrates multimodal MRI features (T1 MRI, DTI, and fMRI) with non-image data to analyze gray matter atrophy and connectivity abnormalities. Evaluated on the ADNI and Peking University Third Hospital (PUTH) datasets, the model achieved 88.71% accuracy for AD vs. NC, 79.68% accuracy for MCI vs. NC, and 82.71% accuracy for AD vs. MCI. While it offers insights into how functional connectivity changes precede structural atrophy, the study’s lack of ROI selection consideration and the non-end-to-end method limit its clinical application.

Recent advancements in integrating sMRI and DTI for AD diagnosis have demonstrated promising classification performance, with reported accuracies ranging from 88.71% (Tian et al. 2023) to 98.42% (Houria et al. 2023). However, despite these gains, several technical and methodological concerns limit the generalizability and clinical readiness of these models. Many studies, including MADNet (Li et al. 2024) and CNN4AD (Fang et al. 2022b), rely on relatively small or homogeneous datasets (e.g., ADNI, XWNI), which may not reflect the variability seen in real-world clinical populations. Additionally, preprocessing strategies like 3D-to-2D reslicing (Houria et al. 2023) and ROI-specific analysis (Aderghal et al. 2020) may constrain spatial and temporal information critical to disease staging. While models like EH-GCN (Tian et al. 2023) and DFCGCN (Meng and Zhang 2023) attempt to incorporate brain connectivity and multi-scale graph structures, many omit dynamic region-of-interest (ROI) selection or rely on handcrafted parcellations, introducing bias and reducing adaptability across institutions. Notably, only a minority of approaches address interpretability using tools such as Grad-CAM (Li et al. 2024), despite the clinical necessity of transparent AI. Moreover, the use of voting-based decision rules (Houria et al. 2023) and complex architectures requiring extensive retraining (e.g., re-transfer learning in CNN4AD) raises questions about scalability and reproducibility. To enhance clinical translation, future research should emphasize larger multi-site datasets, automated ROI extraction, and multimodal fusion strategies that balance performance with interpretability and deployment feasibility.

From Table 11, the study (Kaka and Satya Prasad 2022) presents the Differential Evolution-Multiclass Support Vector Machine (DE-MSVM) method for Alzheimer’s classification, which effectively mitigates overfitting and optimization challenges. The approach utilizes AlexNet for feature extraction and Differential Evolution for feature selection, enhancing the model’s performance. Evaluated on the ADNI dataset, the DE-MSVM method achieves a high classification accuracy of 98.13%, outperforming existing methods in Alzheimer’s diagnosis and providing a robust solution for improved prediction. Another study (Wen et al. 2021) extends an open-source framework for reproducible Alzheimer’s disease (AD) classification by incorporating diffusion MRI data, enabling systematic evaluation of preprocessing, feature extraction, and classification techniques. The method achieved a balanced accuracy (BA) of 94% for AD vs. NC, 74% for NC vs. MCI, and 80% for pMCI vs. sMCI. Despite using a standard diffusion MRI processing pipeline, the study acknowledges that the ADNI diffusion MRI data was not acquired using the latest methods. The research highlights the importance of proper feature selection and validation, providing publicly available tools and code for reproducibility. A different study (Lei et al. 2020) introduces a multi-task learning framework that combines low-rank self-calibrated functional and structural brain networks for enhanced Alzheimer’s disease diagnosis and early detection of mild cognitive impairment (MCI). The method constructs structural and functional brain networks separately and utilizes a brain network functional connection analysis method based on individual subjects. The approach achieved high classification accuracy on the ADNI dataset with results such as SMC vs. NC (82.95%), NC vs. EMCI (85.23%), NC vs. LMCI (87.80%), SMC vs. EMCI (84.09%), SMC vs. LMCI (90.24%), and EMCI vs. LMCI (81.71%). This framework significantly improves AD and MCI classification by integrating multimodal neuroimaging data and selecting discriminative features through a joint non-convex regularizer.

In conclusion, early detection frameworks for Alzheimer’s disease (AD) have seen significant advancements with deep learning models and multimodal imaging. Recent studies have reported classification accuracies up to 99.5% using CNNs on combined MRI and PET data. Multimodal fusion techniques consistently outperform single-modality approaches, improving accuracy by 15-20%. Hybrid models, such as SVM combined with deep learning, have achieved 95% accuracy in distinguishing AD from healthy controls. Additionally, advanced optimization algorithms and feature selection methods have reduced processing times by 10-30%. While challenges remain in handling imbalanced datasets and ensuring model generalizability, these data-driven advancements show great potential for enhancing early AD diagnosis and clinical outcomes.

***Imaging and Clinical based approaches***

Imaging and clinical approaches have become essential in the detection and prognosis of Alzheimer’s disease (AD), combining neuroimaging techniques with biomarker values, demographic information and cognitive assessments to improve diagnostic accuracy and prediction of disease progression. These methods provide a more comprehensive view of the disease, improving diagnostic accuracy and early detection shown in Table 12 and 13. A recent study (Sheng et al. 2024) presents an advanced multimodal classification framework for Alzheimer’s diagnosis, integrating MRI, PET, and CSF biomarkers with an enhanced Harris Hawks Optimization algorithm (ILHHO) and a Kernel Extreme Learning Machine (KELM) classifier. By leveraging iterative mapping (IM) and a local escaping operator (LEO), the method achieves superior accuracy (99.2% for AD vs. NC), significantly outperforming single-modality approaches. Despite its promising results, further validation on larger, multi-center datasets is needed to confirm its generalizability and clinical applicability. Another study (Zhu et al. 2023) introduces the Deep Multi-modal Discriminative and Interpretability Network (DMDIN) for Alzheimer’s disease diagnosis, integrating MRI, PET, and CSF biomarkers. By aligning samples in a common discriminative space and identifying key brain regions, the method enhances interpretability while improving classification performance. Utilizing deep multimodal discriminative representation (DMDR) and linear multimodal knowledge distillation (LMKD), DMDIN achieves high accuracy across ADNI datasets, outperforming existing approaches in AD and MCI classification. The Multi-Modal Multi-Scale Transformer Fusion Network (MMTFN), which integrates MRI, PET, and CSF biomarkers for AD diagnosis, uses 3D multi-scale residual blocks with Transformers to capture multi-modal dependencies and enhance feature representation. This method achieves a high classification accuracy of 94.61% on the ADNI dataset, demonstrating strong generalization ability and robustness, despite its high computational cost (Miao et al. 2024). A study (Zhang et al. 2023a) on the MCAD framework introduces a multi-modal cross-attention approach for AD diagnosis, integrating structural MRI, FDG-PET, and CSF biomarkers to enhance classification performance. By leveraging two encoders and a multi-modal interaction module, the method captures fine-grained cross-modal relationships, achieving an accuracy of 91.07% for AD vs. NC classification on the ADNI dataset, though its robustness across diverse datasets requires further validation. Another novel approach (Zhang et al. 2021b) presents a multimodal neuroimaging framework for AD and MCI classification, integrating MRI, PET, and CSF biomarkers. By employing regularization-based feature selection and multi-modal kernel learning, this approach effectively captures structural sparsity and complementary modality information, achieving a classification accuracy of 90.21% on the ADNI dataset. The method is theoretically proven to converge to a global optimum, though its scalability is challenged by high memory requirements when handling large subject cohorts. The LDF method (Li et al. 2020) integrates low-rank representation for noise reduction and discriminant correlation analysis for multimodal feature fusion in Alzheimer’s disease diagnosis, leveraging MRI, PET, and CSF biomarkers. It achieves 90.52% accuracy in AD vs. NC classification on the ADNI-1 dataset, demonstrating its effectiveness in multimodal data integration, though it faces challenges in preserving contextual relationships within modalities. A machine-learning-based (Bucholc et al. 2019) Clinical Decision Support System (CDSS) for early Alzheimer’s detection integrates MRI, PET, and CSF biomarkers. Using models like Kernel Ridge Regression (KRR), Support Vector Machines (SVM), K-Nearest Neighbors (KNN), and Random Forest (RF), the system achieves an AUC of 94.9%, providing an objective and continuous assessment of Alzheimer’s severity. However, it does not handle missing data, which may limit its clinical applicability. The Multi-modal (Kim and Lee 2018) Sparse Hierarchical Extreme Learning Machine (MSH-ELM) for Alzheimer’s and mild cognitive impairment (MCI) diagnosis integrates MRI, FDG-PET, and CSF biomarkers to extract joint hierarchical feature representations. The model achieves high classification accuracy, with 97.12% for AD vs. NC and 87.09% for MCI vs. NC, outperforming conventional approaches, though challenges remain in interpreting the hierarchical feature representation. A multiple kernel-learning framework (Liu et al. 2014) for AD classification incorporates group lasso regularization and Fourier transform on the Gaussian kernel to enhance multimodal feature selection. This method achieves 87.12% accuracy for HC vs. pMCI classification on the ADNI dataset, demonstrating its effectiveness in early AD detection. Lastly, the Multi-modal Multi-task (M3T) learning framework (Zhang and Shen 2012), which jointly predicts clinical stages, cognitive decline, and MCI conversion using MRI, FDG-PET, and CSF data, performs both regression and classification tasks. It demonstrates high accuracy (AD vs. HC: 93.3%, MCI vs. HC: 83.2%) and strong correlation with cognitive scores, showcasing its potential for both diagnosis and prognosis in AD.

**Table 12:** Detailed review of imaging and clinical based (MRI+PET+CSF) approaches

Study	Modalities	AI techniques	Datasets	Metric	Key strengths	Limitations
Sheng et al. (2024)	Imaging (MRI + PET) + CSF	Enhanced Harris Hawks Optimization (ILHHO) + KELM classifier + Iterative Mapping (IM) + Local Escaping Operator (LEO)	ADNI	AD vs. NC ACC 99.2%; MCI vs. AD ACC 92%; MCI vs. NC ACC 94.6%	Multimodal classification outperforms single modalities	Needs validation on larger, multi-center datasets
Zhu et al. (2023)	Imaging (MRI + PET) + CSF	Deep multimodal discriminative representation (DMDR) + linear multimodal knowledge distillation (LMKD)	ADNI	ADNI1: AD vs. NC ACC 96.75%; MCI vs. NC ACC 86.56%; MCI-C vs. MCI-NC ACC 84.38% ADNI2: AD vs. NC ACC 92.45%; MCI vs. NC ACC 86.75%	Not reported	Not reported
Miao et al. (2024)	Imaging (sMRI + PET) + CSF	Multi-modal multi-scale transformer fusion network (MMTFN)	ADNI	AD vs. CN ACC 94.61 ± 0.95%	Good generalization ability and robustness	High computational cost
Zhang et al. (2023a)	Imaging (sMRI + FDG–PET) + CSF	Dual encoders + multimodal interaction (MI) module + classification module	ADNI (1,2)	AD vs. NC ACC 91.07 ± 3.82%; CN vs. MCI ACC 71.26 ± 6.07%; 3-class CN/MCI/AD ACC 64.03 ± 2.49%	Cross-attention captures rich inter-modality interactions informative for AD diagnosis	Not robust across datasets
Zhang et al. (2021b)	Imaging (MRI + PET) + CSF	Regularization-based feature selection + multimodal kernel learning + deep learning	ADNI	ACC 90.21%	Convergence to a global optimum shown theoretically	Large subject counts require substantial kernel-matrix storage
Li et al. (2020)	Imaging (MRI + PET) + CSF	Low-rank representation + discriminant correlation analysis + KNN + SVM	ADNI1	AD vs. NC ACC 90.52%; NC vs. MCI ACC 73.49%; AD vs. MCI ACC 72.54%	Noise reduction + subspace feature learning cuts noise and redundancy	Inter-modal contextual relationships may be degraded
Bucholc et al. (2019)	Imaging (MRI + PET) + CSF	KRR; SVM; KNN; RF	ADNI	AUC 94.9%	Decision support tool	Does not handle missing data
Kim and Lee (2018)	Imaging (MRI + FDG–PET) + CSF	Multi-modal sparse hierarchical extreme learning machine (MSH-ELM)	ADNI1	AD vs. NC ACC 97.12%; MCI vs. NC ACC 87.09%	Joint hierarchical feature representation	Limited sample size; difficult to interpret joint hierarchical features
Liu et al. (2014)	Imaging (MRI) + CSF	Multiple kernel learning + group lasso reg. + Fourier transform Gaussian kernel	ADNI	HC vs. pMCI ACC 87.12%	Explicit mapping via Fourier transform + random sampling	Not reported
Zhang and Shen (2012)	Imaging (MRI + FDG–PET) + CSF	Multi-modal multi-task (M3T) learning	ADNI	*Exp. 1 (stage est.):* Corr. MMSE 0.697 ± 0.022; Corr. ADAS-Cog 0.739 ± 0.012; AD vs. HC ACC 0.933 ± 0.022; MCI vs. HC ACC 0.832 ± 0.015. *Exp. 2 (2y pred.):* Corr. MMSE change 0.511 ± 0.021; Corr. ADAS-Cog change 0.531 ± 0.032; MCI-C vs. MCI-NC ACC 0.739 ± 0.038	First to jointly predict regression + classification targets from baseline multimodal data	Limited data size

**Table 13:** Detailed review of imaging and clinical based approaches

Study	Modalities	AI techniques	Datasets	Metric	Key strengths	Limitations
Bazargani et al. (2024)	Imaging (MRI) + Cognitive (ADAS-Cog, MoCA, MMSE)	3D CNN + DNN	ADNI2	Precision 97%, Recall 95%, F1-score 95%, AUC 96%, ACC 94%	VR-AI-based system capable of AD diagnosis	High cost of VR technology
Liu et al. (2024b)	Imaging (sMRI + PET) + Clinical (age, MMSE)	Hierarchical Attention-based Multi-task Multi-modal Fusion model (HAMMF) + CHAM	ADNI	AD vs. NC ACC 93.15%	Exhibits good generalization and robustness	Performance variations on unseen data
Liu et al. (2023b)	Imaging (T1 MRI) + Clinical (age, gender, education, APOE4, CDRSB, ADAS, ADAS11, ADAS13, MMSE, RAVLT)	Modality Dropout (MDrop) + Cascaded Modality Transformers (CMTs) + 3MT	ADNI (1,2,3) + AIBL	ADNI ACC 93%; AIBL ACC 96.3%	Handles missing data scenarios; avoids feature leakage	GPU dependency; potential feature leakage
Irfan et al. (2023)	Imaging (MRI + PET) + Cognitive	NCA-F + AdBE + ANN + SVM + NB	ADNI (1,2,3)	ACC 83%	Handles missing values and normalization	Not robust across datasets
Liu et al. (2023a)	Imaging (sMRI + PET) + Clinical (demographic) + GM/WM/CSF tissue images	Patch-based deep multi-modal learning (PDMML)	ADNI	AD vs. MCI vs. NC ACC 80.8%	Discriminative location discovery strategy without prior knowledge	Limited data; loss of lesion info; fixed patch size
Zheng et al. (2024)	Imaging (T1 MRI + amyloid-PET) + Clinical (demographic)	Uncertainty-driven Modality Selection (UMoS)	ADNI	MCI vs. AD ACC 81.7%	First study on personalized modality saving for MCI conversion	Assumes universal access to primary modality
Tu et al. (2022)	Imaging (sMRI) + Clinical + Biological	Geometric algebra-based feature extension + influence-degree-based filtration + ANN + CNN	ADNI + AddNeuroMed	AD vs. NC ACC 96.2%; sMCI vs. pMCI ACC 87.4%	(not reported)	Cannot handle missing modalities
Kumari et al. (2022)	Imaging (MRI + FDG-PET + PiB-PET) + Cognitive	Adaptive Hyperparameter Tuning RF Ensemble (HPT-RFE)	ADNI	AD vs. NC ACC 100%; MCI vs. NC ACC 91%; AD vs. MCI ACC 95%	Computationally much faster	Binary classification only
El-Sappagh et al. (2022b)	Imaging (MRI) + Clinical	LSTM	ADNI	AD vs. sMCI vs. pMCI vs. NC ACC 82.8%; MAE 0.1375	First divide-and-conquer concept for AD	Non-explainable model
Pang et al. (2021)	Imaging (MRI + PET) + Cognitive	Multi-task learning + DT + RF + SVM + XGBoost	ADNI	AD vs. MCI vs. NC ACC 72%	Introduced multi-modal fusion with comparative experiments	Data imbalance and missing sets
Zhang et al. (2021a)	Imaging (MRI) + Clinical (age, gender, MMSE, ApoE4)	Deep reinforcement learning + CNN	ADNI, AIBL, NACC	ADNI ACC 95.6 ± 2.5%, AUC 99.6 ± 0.2%; AIBL ACC 95.8 ± 0.7%, AUC 97.9 ± 0.2%; NACC ACC 90.7 ± 1.0%, AUC 96.1 ± 0.3%	Determines high-risk AD areas accurately	Study includes only AD vs. NC
Zhang et al. (2019)	Imaging (MRI + FDG-PET) + Clinical	CNN	ADNI + Huaihe Hospital	AD vs. NC ACC 98.61%; CN vs. MCI ACC 88.15%; MCI vs. AD ACC 88.01%	(not reported)	(not reported)

Recent multimodal approaches for AD diagnosis demonstrate clear gains in diagnostic accuracy, leveraging combinations of MRI, PET, CSF biomarkers, and advanced machine learning architectures. Across the studies reviewed, classification accuracy for AD vs. NC ranges from 87.12% to 99.2%, with the ILHHO-KELM model achieving the highest reported accuracy (99.2%) through optimization-enhanced learning. Despite these gains, generalizability remains a concern, as most models, such as MMTFN (94.61%) and MCAD (91.07%); are validated primarily on the ADNI dataset, limiting insights into performance across diverse clinical populations. Interpretability is addressed in methods like DMDIN, which identifies key brain regions, but many others, such as MSH-ELM (97.12% accuracy), still suffer from a lack of transparency in decision-making. Computational complexity and scalability are recurring limitations, particularly in transformer-based and kernel-learning frameworks. Furthermore, few models (e.g., M3T and CDSS) integrate longitudinal or prognostic outputs, suggesting a research gap in predicting disease progression alongside classification. In summary, while multimodal models offer strong classification metrics, future efforts should prioritize robustness, interpretability, and scalability to ensure clinical translation.

A recent study (Bazargani et al. 2024) presents a VR-AI-based system for early detection and prognosis of AD, integrating MRI data and cognitive assessments (ADAS-Cog, MoCA, MMSE) with a 3D CNN + DNN model. The system leverages virtual reality (VR) technology to create an age-appropriate diagnostic environment, achieving high performance (Acc: 94%, AUC: 96%, F1-score: 95%), though the high cost of VR technology remains a challenge for widespread implementation. Another study (Liu et al. 2024b) introduces the Hierarchical Attention-based Multi-task Multi-modal Fusion (HAMMF) model, incorporating MRI, PET, age, and MMSE scores for AD classification. The model achieves 93.15% accuracy in AD vs. NC classification, demonstrating strong generalization and robustness, but performance variations on unseen data indicate the need for further validation. The Multi-Modal Mixing Transformer (3MT) (Liu et al. 2023b) integrates T1 MRI and clinical data (demographics, cognitive scores, and APOE4 genotype) for AD classification and MCI prognosis, leveraging Modality Dropout (MDrop) and Cascaded Modality Transformers (CMTs) to handle missing data scenarios. It achieves state-of-the-art accuracy (93% on ADNI, 96.3% on AIBL), although concerns about potential feature leakage exist. A study (Irfan et al. 2023) on early dementia detection integrates MRI, PET, and cognitive assessments using Neighborhood Component Analysis with Correlation-Based Filtration (NCA-F) and AdaBoost Ensemble (AdBE), achieving 83% accuracy across ADNI datasets. Despite its effectiveness, the method lacks robustness across diverse datasets, highlighting the need for further validation. The Patch-based Deep Multi-Modal Learning (PDMML) framework (Liu et al. 2023a) uses sMRI, PET, clinical data, and tissue images (GM, WM, CSF) for AD classification, autonomously identifying lesion-sensitive regions without prior knowledge. The model achieves 80.8% accuracy, although challenges include limited data and potential loss of local lesion information. The Uncertainty-driven Modality Selection (UMoS) framework (Zheng et al. 2024) predicts MCI conversion to AD, integrating T1 MRI, amyloid-PET, and demographic data while dynamically selecting necessary imaging modalities. The model achieves 81.7% accuracy, but UMoS assumes all patients must have access to a primary modality, which may limit flexibility. Another multimodal fusion model (Tu et al. 2022) integrates sMRI, clinical, and biological data for AD diagnosis, achieving 96.2% accuracy for AD vs. NC and 87.4% for sMCI vs. pMCI. It outperforms existing methods, but cannot handle missing multi-modal data, limiting its applicability in real-world clinical settings. The Adaptive Hyperparameter Tuning Random Forest Ensemble Classifier (HPT-RFE) (Kumari et al. 2022) leverages MRI, FDG-PET, PiB-PET, and cognitive assessments for AD diagnosis, achieving 100% accuracy for AD vs. NC, 91% for MCI vs. NC, and 95% for AD vs. MCI. Despite its success, the study is limited to binary classification, restricting its adaptability to more complex diagnostic scenarios. A two-stage deep learning framework (El-Sappagh et al. 2022b) employing longitudinal multimodal data for AD classification and prognosis achieves 82.8% accuracy for AD vs. sMCI vs. pMCI vs. NC and a mean absolute error of 0.1375. Although the model outperforms traditional methods, its lack of explainability poses a challenge for clinical adoption. A multi-task learning algorithm integrates MRI, PET, biomarkers, and cognitive scores from ADNI to enhance early AD detection, achieving 72% accuracy in multiclass classification. The study (Pang et al. 2021) proposes a scalable multimodal data platform architecture, but challenges such as missing data and class imbalances remain. The multimodal deep reinforcement learning model (Zhang et al. 2021b) integrates MRI and clinical features (age, gender, MMSE, ApoE4) for AD diagnosis, achieving 95.6% accuracy with 99.6% AUC. The model generates pixel-level disease probability maps but is limited to binary classification for AD vs. NL health conditions. Lastly, a deep learning model combining MRI, FDG-PET, and clinical data diagnoses AD, using CNN architecture to process multi-modal neuroimaging and neuropsychological assessments. The model achieves 98.61% accuracy for AD vs. NC, 88.15% for CN vs. MCI, and 88.01% for MCI vs. AD, demonstrating excellent performance in classification across datasets (Zhang et al. 2019).

Imaging and clinical-based approaches for Alzheimer’s disease (AD) early detection have demonstrated strong classification accuracy, ranging from 80.8% to 100%, with models like HPT-RFE (100%) and Zhang et al.’s deep learning model (98.61%) leading the way. The ILHHO-KELM model, integrating MRI, PET, and CSF biomarkers, achieved 99.2% accuracy. However, generalizability remains a challenge, as many models, such as MMTFN (94.61%) and MCAD (91.07%), are validated primarily on single datasets like ADNI. Computational complexity and lack of interpretability also hinder clinical applicability. Additionally, most models focus on cross-sectional diagnosis, lacking predictive capacity for disease progression. Future research should prioritize improving generalizability, interpretability, and predictive capabilities across diverse populations. 

***Imaging and Genetic based approaches***

Imaging and genetic approaches have gained prominence in AD research, aiming to bridge the gap between neuroimaging biomarkers and genetic risk factors. While neuroimaging techniques provide structural and functional insights, genetic data, such as single nucleotide polymorphisms (SNPs), offer a deeper understanding of disease susceptibility and progression shown in Table 14. However, very few studies have effectively integrated these modalities, highlighting a significant research gap. The challenges of data heterogeneity, missing information, and the need for explainable AI models further limit advancements in this area. Given the potential of multimodal fusion for improving AD classification and prognosis, there is a vast scope for further exploration and development.

**Table 14:** Detailed review of imaging and genetic based approaches

Study	Modalities	AI techniques	Datasets	Metric	Key strengths	Limitations
Bi et al. (2024)	Imaging (fMRI) + Genetic (SNP)	Hypergraph Structural Information Aggregation GANs (HSIA-GAN)	ADNI	AD vs. NC: ACC 88.17%; LMCI vs. AD: ACC 85.06%; EMCI vs. LMCI: ACC 83.75%	Complete data fusion; explainability	Not reported
Hao et al. (2023)	Imaging (VBM + FDG + AV45) + Genetic (SNPs)	Multimodal Self-paced Locality-preserving Learning (MSLPL) + MK-SVM	ADNI	AD vs. NC: ACC 95.14%; CN vs. LMCI: ACC 82.85%; EMCI vs. LMCI: ACC 76.91%	Preserves inherent structural relationships	Ignored consistent ROI feature selections; generalization/scalability need verification
Zhou et al. (2019)	Imaging (T1 MRI + PET) + Genetic (SNP)	Latent feature representation learning	ADNI 1	sMCI vs. pMCI: ACC 74.3%	Integrates latent feature learning with classifier training in a unified framework	Linear projection; incomplete multi-modality data
Sheng et al. (2022)	Imaging + Genetic	Integrated Fisher score + multi-modal multi-task feature selection	ADNI 2	AD vs. NC: ACC 98%; NC vs. EMCI: ACC 82%; CN vs. LMCI: ACC 86%; EMCI vs. LMCI: ACC 80%; EMCI vs. AD: ACC 88%; LMCI vs. AD: ACC 72%	Not Reported	Limited sample size

This study (Bi et al. 2024) presents HSIA-GAN (Hypergraph Structural Information Aggregation GANs), a deep learning model integrating fMRI and genetic (SNP) data for AD classification. The model effectively fuses low-order and high-order hypergraph relationships, enhancing disease-related feature extraction. It achieves 88.17% accuracy for AD vs. NC, 85.06% for LMCI vs. AD, and 83.75% for EMCI vs. LMCI on the ADNI dataset. While offering complete data fusion, the model faces challenges in explainability, but it provides valuable insights into AD-related biomarkers and progression patterns. Another study (Hao et al. 2023) introduces Multimodal Self-Paced Locality-Preserving Learning (MSLPL), a novel framework for AD classification that integrates neuroimaging (VBM, FDG, AV45 PET) and genetic (SNPs) data. By adaptively weighing sample contributions to reduce noise, MSLPL preserves inherent structural relationships in multimodal data. Combined with multi-kernel SVM (MK-SVM) for final classification, the model achieves 95.14% accuracy for AD vs. NC, 82.85% for CN vs. LMCI, and 76.91% for EMCI vs. LMCI on ADNI data. Despite its effectiveness, the method ignores consistent ROI feature selections and requires further validation for generalization and scalability. A third study (Zhou et al. 2019) presents a latent feature representation learning method for AD diagnosis, integrating T1 MRI, PET, and genetic (SNP) data. The approach unifies latent feature learning and classifier training to effectively handle missing multi-modality data, leveraging both common and modality-specific latent representations. Evaluated on 737 subjects from ADNI 1, the method achieves 74.3% accuracy for sMCI vs. pMCI classification. While the approach preserves inter-modality associations, it relies on linear projection and may be affected by incomplete multi-modal data. Another study (Sheng et al. 2022) introduces an integrated Fisher score and multi-modal, multi-task feature selection method for AD classification using brain imaging and genetic features. The approach enhances classification accuracy and identifies interrelated biomarkers, achieving 98% accuracy for AD vs. NC and strong performance across other disease stages. Despite the limited sample size, the model effectively captures key disease patterns, supporting its potential for biomarker discovery and early diagnosis. This limited body of research underscores the need for more studies in this area, as integrating genetic and neuroimaging data could significantly enhance the early detection and prognosis of AD.

Imaging and genetic-based approaches for Alzheimer’s disease (AD) early detection have shown promising results, with classification accuracies ranging from 74.3% to 98%, depending on the model and dataset. Notably, models like HSIA-GAN (88.17% accuracy) and MSLPL (95.14% accuracy) demonstrate effective integration of neuroimaging and genetic data, revealing potential for improved classification. However, challenges persist, including issues with data heterogeneity, missing data, and the need for more interpretable models. The generalizability of these approaches remains a concern, as most studies rely heavily on the ADNI dataset. Additionally, methods like Zhou et al.’s latent feature representation approach (74.3% accuracy) and Sheng et al.’s integrated Fisher score model (98%) face limitations in handling missing data and ensuring scalability. Despite these obstacles, the integration of genetic data with neuroimaging holds great potential for enhancing early diagnosis and prognosis, emphasizing the need for further research to address data integration, generalization, and model transparency.

***Imaging, Clinical and Genetic based approaches ***

Integrating imaging, clinical and genetic data is an emerging and powerful approach for advancing AD diagnosis and prognosis. Table 15 shows while neuroimaging provides critical structural and functional insights into the brain, genetic markers such as SNPs can help identify individuals at risk and track disease progression. Clinical data further enrich these models by offering cognitive, demographic, and biomarker information. Despite the significant potential, this area remains underexplored, with few studies successfully combining these modalities in an interpretable and robust manner. There is considerable scope for further research to enhance the accuracy, scalability, and clinical applicability of such integrated approaches.

**Table 15:** Detailed review of imaging, clinical and genetic based approaches

Study	Modalities	AI techniques	Datasets	Metric	Key strengths	Limitations
Lei et al. (2024a)	Imaging (MRI) + Genetic (SNP) + Biomarker (CSF)	Feature Induction Learning (FIL) + Dual Multilevel Graph Neural Network (DMGNN)	ADNI	ACC – 93.24%	Reduces “modality competition” in multi-modal fusion learning	Inability to process multi-modal data lacking complete information
Parvin et al. (2024)	Imaging (MRI) + Tabular Data + Genetic Data	Graph Neural Networks + CNN + XAI	OASIS	Not Reported	Explainability and interpretability	Not Reported
Wang and Xu (2022)	Imaging (MRI) + Clinical (Age, Gender) + Genetics (APOE) + Biomarker (P-tau181)	Feature Pyramid Network + self-attention fusion method	ADNI (1, 2/GO, 3)	AD vs. MCI: ACC – 90.5%, SEN – 88%, SPE – 95.7%, AUC – 91.5%	Light-weight and effective all-level FPN for MRI	Test dataset was not large
Qiang et al. (2023)	Imaging (sMRI) + Clinical (demographics, neuropsychology) + Genetic (APOE)	Dual Attention CNN + Multilayer Perceptron (MLP)	ADNI	AD vs. MCI: ACC – 93%; MCI vs. CN: ACC – 82.4%	Not Reported	Network is relatively large with many parameters; input patch is fixed
Venugopalan et al. (2021)	Imaging (cross-sectional MRI) + Genetic (SNP) + Clinical	Stacked Denoising Auto-encoders + 3D CNN	ADNI (1, 2 & GO)	AD vs. MCI vs. NC: ACC – 78%	Novel interpretation method	Not Reported
Brand et al. (2020a)	Imaging (MRI) + Genetic (SNP) + Clinical (Demographic)	Joint Multi-Modal Longitudinal Regression and Classification	ADNI	BACC – 0.584 ± 0.033	Includes three key regularization terms for temporal and structural relationships across data	Not Reported
Tong et al. (2017)	Imaging (1.5T MRI + PET) + Biomarker (CSF) + Genetic	Nonlinear Graph Fusion	ADNI	AD vs. NC: ACC – 86.2%; CN vs. MCI: ACC – 73.1%; CN vs. MCI vs. AD: ACC – 54.1%	Full use of local structures in different graphs	Sample size is limited; missing data not handled
Gray et al. (2013)	Imaging (T1 MRI + FDG-PET) + Biomarker (CSF) + Genetic	Pairwise similarity measures via Random Forest classifiers	ADNI	AD vs. NC: ACC – 89.0%; NC vs. MCI: ACC – 74.6%; pMCI vs. sMCI: ACC – 58.0%	Not Reported	Not Reported

This study (Lei et al. 2024a) proposes a Feature Induction Learning (FIL) and Dual Multilevel Graph Neural Network (DMGNN) framework for AD diagnosis, integrating MRI, genetic (SNP), and biomarker (CSF) data. The method effectively mitigates modality competition in multi-modal fusion learning by addressing feature heterogeneity and leveraging complementary information across genetic, imaging, protein, and clinical data. Evaluated on ADNI, the approach achieved 93.24% accuracy, outperforming state-of-the-art techniques. However, its limitation lies in handling missing multimodal data, which could affect its robustness in real-world applications. Another study (Parvin et al. 2024) introduces a multimodal framework for early AD detection, integrating MRI imaging, tabular data, and genetic information. Utilizing Graph Neural Networks (GNNs), Convolutional Neural Networks (CNNs), and Explainable AI (XAI) techniques, the method enhances both prediction accuracy and interpretability. The approach incorporates knowledge graphs and layer-wise relevance propagation, providing transparent decision-making to aid medical professionals in understanding diagnostic results. The model is evaluated on the OASIS dataset, emphasizing its capability for explainability and early detection.

A third study (Wang and Xu 2022) presents AANet, an attentive all-level fusion system for early AD diagnosis, integrating 3D MRI, demographics (age, gender), genetics (APOE), and blood biomarkers (P-tau181). The model employs a Feature Pyramid Network (FPN) and a self-attention fusion method, ensuring lightweight yet effective multi-level feature integration. Evaluated on ADNI (1, 2/GO, and 3) datasets, AANet achieves 90.5% accuracy in AD vs. MCI classification, with 88% sensitivity, 95.7% specificity, and 91.5% AUC, demonstrating robust performance despite a limited test dataset. This study (Qiang et al. 2023) introduces DANMLP, a dual attention convolutional neural network (CNN) combined with a multilayer perceptron (MLP) for AD and mild cognitive impairment (MCI) diagnosis. Utilizing sMRI, clinical (demographics, neuropsychology), and genetic (APOE) data, DANMLP achieves 93% accuracy in AD vs. MCI classification and 82.4% in MCI vs. CN classification. The model provides interpretable visualizations of affected brain regions, supporting clinical decision-making. Despite its large number of parameters and fixed input patch size, DANMLP demonstrates high accuracy and robustness in multimodal AD diagnosis. Another study (Venugopalan et al. 2021) leverages stacked denoising auto-encoders and a 3D CNN to integrate MRI, genetic (SNPs), and clinical data for AD, mild cognitive impairment (MCI), and normal control (NC) classification. Using the ADNI (1, 2 & GO) dataset, the model achieves 78% accuracy, outperforming single-modality approaches. Key biomarkers identified, such as the hippocampus, amygdala, and RAVLT scores, align with established AD pathology. Additionally, the study introduces a novel interpretation method, enhancing the transparency of deep learning-based AD diagnosis. This study (Brand et al. 2020a) proposes a novel machine learning algorithm using the Multi-Block Alternating Direction Method of Multipliers (ADMM) to predict cognitive scores and AD diagnoses based on multi-modal longitudinal clinical data. By integrating MRI, genetic (SNPs), clinical (demographic), and cognitive information, the model achieves state-of-the-art performance. It leverages structural information from clinical data, validating important brain and genetic biomarkers associated with AD, ultimately improving diagnosis and understanding of cognitive progression. Another study (Brand et al. 2020b) introduces the Joint Multi-Modal Longitudinal Regression and Classification (JMMLRC) method, which integrates genetic (SNP) and brain scan (MRI) data to predict cognitive status and identify Alzheimer’s-related biomarkers. The model incorporates three important regularization terms to capture the temporal and structural relationships of the input data from various perspectives. Evaluated on the ADNI dataset, the model achieves promising results, with a balanced accuracy (BACC) of $0.584 \pm 0.033$. It provides a flexible approach for AD prediction and offers open-source implementation for broader clinical use, supporting early diagnosis and prognosis. This study (Tong et al. 2017) introduces a multi-modality classification framework that employs nonlinear graph fusion to integrate complementary biomarkers from MRI, FDG-PET, CSF, and genetic data for AD diagnosis. The method fully leverages the local structures in different graphs, enhancing the integration of multimodal data. It achieves significant performance improvements over single-modality and linear approaches, with AUCs of 98.1% (AD vs. NC), 82.4% (MCI vs. NC), and 77.9% (three-way classification). However, the study acknowledges that the sample size is limited, and the method doesn’t handle missing data. The classification accuracy for AD vs. NC is 86.2%, for CN vs. MCI is 73.1%, and for the three-way classification (CN, MCI, AD) is 54.1%. Finally, this study (Gray et al. 2013) introduces a multi-modality classification framework that utilizes pairwise similarity measures derived from random forest classifiers to create joint embeddings for AD diagnosis. By combining MRI, FDG-PET, CSF biomarkers, and genetic data, the method improves classification accuracy significantly. It achieves 89% accuracy for the AD vs. NC classification, 74.6% for NC vs. MCI, and 58% for pMCI vs. sMCI, outperforming single-modality approaches. The study highlights the effectiveness of integrating multiple data sources to enhance diagnostic precision.

The combination of imaging, clinical, and genetic data for early Alzheimer’s disease (AD) detection has demonstrated significant potential, with classification accuracies ranging from 78% to 98% across various models and datasets. Notable approaches, such as the FIL-DMGNN framework (93.24% accuracy) and AANet (90.5% accuracy), demonstrate robust performance by effectively combining MRI, genetic (SNP), and clinical data. However, challenges persist in handling missing multimodal data and ensuring the scalability of these models in real-world clinical settings. While studies like Parvin et al. (XAI-enhanced framework) and Venugopalan et al. (78% accuracy with stacked denoising autoencoders) emphasize model interpretability and transparency, the generalizability of these methods across diverse populations remains a concern. Moreover, models such as DANMLP (93% accuracy) and the JMMLRC method (balanced accuracy of $0.584 \pm 0.033$) highlight the need for improvements in temporal modeling and the handling of longitudinal data. Despite these limitations, the integration of multimodal data holds significant potential for improving AD diagnosis, with future efforts focusing on overcoming data completeness, scalability, and model explainability to ensure clinical applicability.

***Other Multimodality approaches***

Beyond traditional neuroimaging and genetic-based methods, researchers have explored alternative multimodal approaches for AD and Mild Cognitive Impairment (MCI) detection. These methods leverage speech, language, vision, and advanced feature selection techniques to provide cost-effective, non-invasive, and interpretable diagnostic solutions shown in Table 16. While promising, research in this area remains limited, with challenges such as small datasets, model generalizability, and the need for further validation. However, integrating diverse modalities opens new avenues for early diagnosis and improved biomarker identification.

**Table 16:** Detailed review of other multimodal approaches

Study	Modalities	AI techniques	Datasets	Metric	Key strengths	Limitations
Poor et al. (2024)	Speech + Language + Vision data	Mid-level Cross-Transformer	I-CONECT	AUC – 85.3%	Eliminates MRI-related expenses and prerequisites	Not reported
Ortiz-Perez et al. (2023)	Text + Audio	CNN + Transformer (BERT)	DementiaBank Pitt Corpus	ACC – 90.36%	Model explainability	Small dataset
Ilias et al. (2023)	Speech + Transcripts	BERT + ViT + Crossmodal Attention	ADReSS Challenge	ACC – 87.92%	Use of ViT	Limited number of samples

This study (Poor et al. 2024) presents a mid-level cross-transformer model that integrates speech, language, and vision data for the early detection of Mild Cognitive Impairment (MCI). The proposed multimodal co-attention fusion architecture captures complementary interactions across modalities, achieving an AUC of 85.3% on the I-CONECT dataset. Unlike MRI-based approaches, this method offers a cost-effective and accessible alternative, improving diagnostic accuracy over unimodal and bimodal techniques. Another study (Ortiz-Perez et al. 2023) introduces a multimodal deep learning system that integrates text and audio analysis for early dementia detection. Utilizing a CNN and Transformer (BERT) model, the approach achieves 90.36% accuracy on the DementiaBank Pitt Corpus. The study also emphasizes model explainability, analyzing predictions and identifying linguistic and acoustic patterns associated with dementia. Despite the small dataset, the proposed method demonstrates promising results for non-invasive, speech-based screening. A third study (Ilias et al. 2023) presents a multimodal deep learning approach for AD classification, leveraging speech and transcript data. The model integrates BERT for text processing, Vision Transformers (ViT) for speech spectrograms/MFCCs, and Crossmodal Attention for feature fusion. Achieving 87.92% accuracy on the ADReSS Challenge dataset, the approach effectively captures complementary patterns between speech and text. Despite the limited sample size, the study highlights the potential of speech-based biomarkers for non-invasive Alzheimer’s detection.

These alternative multimodal approaches demonstrate the potential of integrating diverse data sources for AD detection. However, further research is needed to enhance dataset diversity, improve model generalization, and validate these methods in real-world clinical settings.

#### Layer 2: Disease Progression

***Imaging based approaches***

In the context of AD prognosis, imaging-based multimodal approaches remain relatively rare, as most studies tend to combine neuroimaging with clinical and genetic data for disease progression identification. However, neuroimaging alone offers valuable biomarkers that can aid in predicting disease conversion, particularly through advanced modalities such as MRI, PET, and rsfMRI shown in Table 17. While these imaging techniques demonstrate strong predictive capabilities, challenges persist in optimizing multimodal fusion and improving generalizability for broader clinical applications.

**Table 17:** Detailed review of imaging-based approaches in disease progression

Study	Modalities	AI techniques	Datasets	Metric	Key strengths	Limitations
Agostinho et al. (2024)	Imaging (MRI + Fluorodeoxyglucose PET + Florbetapir PET + DTI)	Weighted ensemble technique	ADNI (1,2/GO, 3)	ACC: 83.51% (Florbetapir PET), ACC: 78.43% (MRI + Fluorodeoxyglucose PET)	Highlights AV45-PET as a promising neuroimaging biomarker for measuring $\beta$-amyloid burden	Not all multi-modal combinations enhanced prediction accuracy; focused only on imaging without genetic or clinical factors
Gullett et al. (2021)	Imaging (T1 sMRI + rsfMRI)	Semi-supervised SVM	ADRC	ACC: 92.7% (T1), 83.5% (rsfMRI), 94.5% (T1 + rsfMRI)	MRI-driven SVM model	Not Reported

This study (Agostinho et al. 2024) employs a weighted ensemble technique to predict AD progression using multi-modal neuroimaging data from ADNI. Results highlight Florbetapir PET (AV45-PET) as the most effective biomarker for predicting MCI-to-AD conversion (83.51% accuracy), while combining MRI and FDG-PET improved accuracy to 78.43%. Key biomarkers, including the brainstem and corpus callosum, were identified for early detection. However, not all multi-modal combinations enhanced prediction accuracy, and the study did not integrate genetic or clinical data, limiting its scope. Another study (Gullett et al. 2021) employs a semi-supervised SVM model using baseline multimodal MRI (T1 sMRI + rsfMRI) to predict amnestic MCI (aMCI) to dementia conversion. The model achieves 94.5% accuracy when combining T1 and rsfMRI, outperforming single-modality approaches (T1: 92.7%, rsfMRI: 83.5%). Results highlight the medial temporal lobes in the limbic system as key regions for early detection and disease progression tracking.

These studies reinforce the significance of neuroimaging in disease prognosis and underscore the importance of selecting optimal imaging modalities for accurate prediction. Future work should explore integrating genetic, clinical, and biomarker data to improve early detection and progression forecasting.

***Imaging and Clinical based approaches***

Disease progression prediction in AD has traditionally relied on combining imaging data with clinical features, as these approaches provide rich information for early diagnosis and monitoring of the disease’s trajectory shown in Table 18. While various imaging modalities like MRI, PET, and CSF biomarkers have proven useful in identifying structural changes and abnormal biomarkers, integrating them with clinical data such as cognitive scores, demographics, and clinical test results can further enhance predictive performance. Below are several examples of multimodal approaches that combine imaging and clinical data to predict Alzheimer’s progression.

**Table 18:** Detailed review of imaging and clinical based approaches in disease progression

Study	Modalities	AI techniques	Datasets	Metrics	Key strengths	Limitations
Lee et al. (2024)	Imaging (T1 MRI + $\alpha$PET) + Clinical (demographic + clinical test score)	CNN + MLP + GBM	ADNI + 4 Tertiary hospitals	AUC: 0.875	Diverse data	Limited sample size (196 subjects)
Cheng et al. (2024)	Imaging (MRI) + Clinical (demographic)	LSTM-TSGAIN	ADNI	ACC: 0.872 ± 1.4%, Precision: 0.86 ± 0.02, Recall: 0.86 ± 0.02, F1: 0.85 ± 0.01, MCC: 0.78 ± 0.03, Kappa: 0.77 ± 0.04, CSI: 0.77 ± 0.04	(not reported)	Reliance on software-processed ROI features might omit crucial details
Shetty et al. (2023)	Imaging (MRI) + Biomarkers (ABETA, TAU, PTAU) + Cognitive scores (ECogPt Mem, ECogPt Total) + Demographic (Age, Gender, Education, APOE4)	GRU-based RNN + SVM + Ensemble classifier	ADNI	ACC: 96%	Leverages feature correlation to enhance detection accuracy	(not reported)
Cai et al. (2023)	Imaging (MRI) + Cognitive Scores (ADAS-cog-13, MMSE, FAQ, CDR-SB) + Demographics (Age, gender, education, baseline DX, APOE4)	Explainable Boosting Machines (EBM)	ADNI	With interactions: ACC: 0.75, SEN: 0.78, SPE: 0.73, AUC: 0.81; Without interactions: ACC: 0.72, SEN: 0.76, SPE: 0.68, AUC: 0.78	Focus on interpretability using EBM	Single dataset may limit generalizability
Zhao et al. (2023c)	Imaging (MRI + FDG-PET + AV45-PET) + Cognitive Tests (RAVLT, ADAS-Cog, FAQ, MMSE, CDRSB, MOCA) + CSF Measures (Beta-amyloid, tau, p-tau)	Multi-task evidential sequence learning + Evidential deep learning + DWA	TADPOLE	mAUC: 0.942 ± 0.0091, BCA: 0.879 ± 0.0098, adasMAE: 4.280 ± 0.40	Multi-task learning and uncertainty estimation	Limited features may not fully capture AD progression
Bapat et al. (2024)	Imaging (MRI) + Cognitive data	3D CNN	ADNI (ADNI1, GO, 2, 3)	ACC: 0.8347 ± 0.0261	Effective transfer learning and explainability	Dependency on transfer learning; fixed 4-year window
Akhtar et al. (2022)	Imaging (MRI + PET) + Biomarker (CSF)	Deep Neural Networks (DNNs)	ADNI	MAE: 0.13, ACC: 86.9%, AUC: 92%, SEN: 67.7%, SPE: 92.3%	Cost-effective approach	Limited to 17 neuropsychological measures
Tabarestani et al. (2022)	Imaging (MRI + PET) + Biomarker (CSF) + Clinical (cognitive tests, risk factors)	KTMnet (Kernelized & Tensorized Multitask Network)	ADNI	ACC: 66.85 ± 3.77	Effective modality fusion, kernelization & tensorization	Seesaw effect during hyperparameter tuning
El-Sappagh et al. (2022a)	Imaging (MRI) + Cognitive Scores + Neuropsychological tests	KNN, DT, SVM, XGB, RF, MLP (ensemble)	ADNI	RF, XGB, KNN: ACC = 86.17%, Precision = 88.10%, Recall = 86.17%, F1 = 86.67%, BA = 84.41%	(not reported)	(not reported)
Pang et al. (2021)	Imaging (MRI + PET) + Cognitive Scores (MMSE + ADAS-cog)	Multi-task learning (MTL), SVM + XGBoost	ADNI	(not reported)	Multi-task learning	Overfitting in SVM models
Young et al. (2014)	Imaging (MRI + PET) + Biomarker (CSF)	Probabilistic Generative Model + Event-based Model (EBM)	ADNI	Class. ACC: 99%, BA (MCI$\rightarrow$AD, 3y): 77%, BA (CN$\rightarrow$MCI, 5y): 76%	Identifies biomarker sequencing and staging	Uncertainty in biomarker ordering across subgroups
Zhang and Shen (2012)	Imaging (MRI + FDG-PET) + CSF biomarkers	Multi-modal multi-task (M3T)	ADNI	AD vs HC: ACC = 0.933 ± 0.022, MCI vs HC: ACC = 0.832 ± 0.015, MCI-C vs MCI-NC: ACC = 0.739 ± 0.038	(not reported)	Limited by missing multimodal and clinical data
Rahim et al. (2023)	Imaging (3T MRI) + Cognitive Scores (ADAS13, FAQ, MMSE, RAVLT) + Demographic data (Age, Gender, Education, APOE4)	3D CNN + BRNN + Multimodal Fusion	ADNI	ACC: 96%, Precision: 99%, Recall: 92%, AUC: 96%	Visual attention maps highlight brain regions confirmed medically	Generalizability concerns

One approach (Lee et al. 2024) integrates T1 MRI, $\alpha$PET, and clinical data (demographics and clinical test scores) into a multimodal machine learning model, achieving an AUC of 0.875 in predicting disease conversion from MCI to AD. The model shows high stability with Gradient Boosting Machine (GBM) and improved performance when excluding T2-FLAIR features, although its small sample size of 196 subjects may impact scalability and generalizability. The LSTM-TSGAIN framework uses adversarial learning and collaborative multi-task learning to improve AD progression prediction, achieving strong performance with an accuracy of 87.2%, precision and recall at 86%, and an F1-score of 85%. However, this model relies on software-processed ROI features, which may limit its ability to capture more detailed brain region information (Cheng et al. 2024). Another study (Shetty et al. 2023) combines GRU-based RNNs, SVM, and an ensemble classifier to predict the progression of mild cognitive impairment (MCI) to AD by integrating MRI, biomarkers like ABETA and TAU, cognitive scores (ECogPt Mem, ECogPt Total), and demographic data. The ensemble classifier achieves an impressive 96% accuracy, outperforming individual modalities and classifiers, showing the power of combining diverse data sources for accurate predictions. Explainable Boosting Machines (EBM) have also been used to predict MCI-to-AD conversion, integrating MRI, cognitive scores (ADAS-cog-13, MMSE, FAQ, CDR-SB), and demographic data. The model emphasizes interpretability, offering insights into key biomarkers and cognitive features over time. The model achieved an accuracy of 0.75, sensitivity of 0.78, specificity of 0.73, and an AUC of 0.81, demonstrating its ability to understand disease progression through interaction effects (Cai et al. 2023).

A multi-task evidential sequence learning model (Zhao et al. 2023c) has been developed to simultaneously predict clinical diagnosis and cognitive scores by integrating MRI, FDG-PET, AV45-PET, cognitive tests, and CSF biomarkers. This model uses dynamic weight averaging to estimate uncertainty, achieving a mean AUC of 0.942 and balanced classification accuracy (BCA) of 0.879. Despite its strong performance, its limited feature set may not fully capture the complexity of AD progression. In another study (Bapat et al. 2024), a 3D CNN model uses longitudinal MRI and cognitive data from ADNI to predict MCI to AD conversion within a four-year window, achieving an accuracy of 83.4%. Transfer learning reduces data requirements and training time, but the model’s reliance on fixed time windows may limit adaptability to data with varying lengths. A deep neural network (DNN) model (Akhtar et al. 2022) predicts MCI to AD conversion by leveraging MRI, PET, and CSF biomarkers, achieving 86.9% accuracy and an AUC of 92%. The model’s use of time-domain biomarkers enhances prediction reliability, making it cost-effective and suitable for low-resource settings. However, it relies on only 17 neuropsychological measures, which could limit its ability to capture the full spectrum of Alzheimer’s progression. KTMnet, a kernelized and tensorized multi task deep learning network, has also been used for AD classification and cognitive score prediction by combining MRI, PET, CSF biomarkers, and clinical data. The model achieves 66.85% accuracy but faces challenges during hyperparameter optimization, affecting model stability (Tabarestani et al. 2022).

An ensemble learning framework (El-Sappagh et al. 2022a) that combines base classifiers (KNN, DT, SVM, XGB, RF, MLP) has demonstrated the power of multimodal integration in AD progression prediction. The best-performing models: RF, XGB, and KNN, achieved 86.17% accuracy, 88.10% precision, and an F1-score of 86.67%, surpassing existing methods, even without neuroimaging. Additionally, a multi-modal machine learning framework using MRI, PET, and cognitive evaluation scores (MMSE, ADAS-cog) has been developed for predicting AD progression. Leveraging multi-task learning (MTL) with SVM and XGBoost, the approach enhances predictive accuracy, though overfitting in SVM models remains a challenge. This underscores the importance of integrating neuroimaging and cognitive assessments to improve diagnosis and disease monitoring (Pang et al. 2021). Another study (Young et al. 2014) presents a probabilistic generative model combined with an event-based model (EBM) for predicting AD progression using MRI, PET, and CSF biomarkers. With a classification accuracy of 99%, it effectively stages patients and predicts progression, offering insights into biomarker sequencing and disease staging. However, uncertainty in the ordering of biomarkers across subgroups remains a challenge. A multi-modal multi-task (M3T) learning framework integrates MRI, FDG-PET, and CSF biomarkers to predict AD progression. This model achieves high classification accuracy for AD vs. HC (93.3%), MCI vs. HC (83.2%), and MCI converters vs. non-converters (73.9%). However, its reliance on multimodal data and the exclusion of clinical scores due to missing data limit its applicability (Zhang and Shen 2012). Lastly, this study (Rahim et al. 2023) introduces a hybrid deep-learning framework combining 3D CNN and BRNN to predict AD progression using 3D MRI and biomarkers. The model, applied to ADNI data, achieves high accuracy (96%), precision (99%), recall (92%), and AUC-ROC (96%). A key strength of the study is its use of visual attention maps to highlight brain regions impacted by Alzheimer’s, with findings medically confirmed. Despite its strong performance, the model’s generalizability remains a concern. This approach offers potential for enhanced AD prediction and clinical interpretation, but challenges remain in broader application.

These studies highlight the potential of combining imaging and clinical data for more accurate AD progression predictions. By leveraging various machine learning techniques and multimodal data integration, these models are advancing the understanding of AD progression, though challenges such as overfitting, limited data, and feature set complexity still need to be addressed. Future research should focus on refining these models and validating them across diverse clinical populations. 

***Imaging, Clinical and Genetic based approaches***

Recent advancements in machine learning have enabled the integration of imaging, clinical, and genetic data to improve AD diagnosis and predict disease progression more accurately shown in Table 19. By leveraging multimodal data, these approaches enhance early detection, track disease heterogeneity, and refine personalized predictions. The following studies present diverse methodologies that combine MRI, PET, CSF biomarkers, cognitive assessments, and genetic information for more precise AD and Mild Cognitive Impairment (MCI) conversion prediction.

A transformer-based model (Yu et al. 2024), AD-Transformer, integrates MRI, clinical, and genetic data, achieving AUC scores of 0.993 for AD diagnosis and 0.845 for MCI conversion prediction. The model surpasses traditional approaches through a cohesive analytical framework that enhances multimodal data fusion. However, limitations in genetic data availability and racial diversity within the ADNI dataset impact its generalizability. A Personalized-Hidden Markov Model (personalized-HMM) leverages MRI, cognitive data, biomarkers (A$\beta$ and pTau), demographic data, and genetic information to track disease progression. Trained on ADNI and AIBL datasets, the model identifies 10 disease-related states across three disease stages and two progression patterns. With a C-Index of $0.923 \pm 0.007$, it demonstrates strong predictive accuracy for time-to-conversion to Alzheimer’s dementia. However, its reliance on MRI and biomarker data remains a limitation (Zhang et al. 2024).

**Table 19:** Detailed review of imaging, clinical and genetic based approaches in disease progression

Study	Modalities	AI techniques	Datasets	Metrics	Key strengths	Limitations
Yu et al. (2024)	Imaging (MRI) + Clinical + Genetic data	AD-Transformer	ADNI	AUC: 0.993 (AD diagnosis), 0.845 (MCI conversion)	Cohesive, interconnected analytical framework	Limited genetic data and racial/ethnic diversity in ADNI
Zhang et al. (2024)	Imaging (MRI) + Clinical (Cognitive, Biomarkers: A$\beta$, pTau, demographic) + Genetic	Personalized-HMM	ADNI + AIBL	C-Index: $0.923 \pm 0.007$	Identifies 10 disease-related states	Reliance on MRI and biomarker data only
Poonam et al. (2024)	Imaging (MRI, PET) + Cognitive + Genetic	SLA-ED LSTM	ADNI	Accuracy: 0.920, AUC-ROC: 0.982	Adaptive model improves with more visits, local and global interpretability	(not reported)
Wang et al. (2024)	Imaging (sMRI) + Clinical + Genetic (SNP biomarkers)	DNN (DISFC)	ADNI	ACC: 92.86%, AUC: 0.939	(not reported)	(not reported)
Franciotti et al. (2023)	Imaging (sMRI) + CSF Biomarkers + Genetic (APOE $\varepsilon$4)	RF + GB + XGB	ADNI	Accuracy: 0.90, PPV: 0.91, NPV: 0.87, Sensitivity: 0.67, Specificity: 0.97	Feature selection, voting procedure	Overfitting in GB and XGB
Al Olaimat et al. (2023)	Imaging (MRI) + Clinical (Demographic + Cognitive) + Genetic	RNN (PPAD and PPAD-Autoencoder)	Train: ADNI, Test: NACC	(not reported)	(not reported)	Limited generalizability across cohorts
Wang et al. (2022)	Imaging (T1 MRI) + Cognitive + Genetic (APOE) + Clinical (Demographic)	CNN + SVC	ADNI + J-ADNI	ACC: 0.88 (ADNI), 0.84 (J-ADNI), AUC: 0.95 (ADNI), 0.91 (J-ADNI)	High generalizability	Limited dataset size, baseline data only
Spasov et al. (2019)	Imaging (sMRI) + Clinical + Genetic (APOE4)	3D CNN + MTL	ADNI	AUC: 0.925, ACC: 86%, Sensitivity: 87.5%, Specificity: 85%	Parameter-efficient CNN, reduces overfitting	Limited to baseline data, no advanced biomarkers
Lee et al. (2019)	Imaging (MRI + PET) + CSF + Genetic (APOE) + Diagnostic	RNN + Multi-modal GRU	ADNI	Accuracy: 81%, AUC: 0.86	Multimodal deep learning approach	May limit generalizability
Nie et al. (2017)	Imaging (MRI + PET) + CSF + Demographic + Genetic (APOE $\varepsilon$4)	MMSE	ADNI	MMSE R: 0.8794, nMSE: 0.1233; ADAS-Cog R: 0.9094, nMSE: 0.0347	(not reported)	(not reported)
Young et al. (2013)	Imaging (MRI + FDG-PET) + CSF + Genetic (APOE)	Gaussian processes, SVM	ADNI	ACC: 74%, AUC: 0.795	Probabilistic multimodal integration	Small sample size
Mirabnahrazam et al. (2023)	Imaging (MRI) + Clinical (Cognitive + biomarkers + demographic) + Genetic	DeepSurv	ADNI	Ctd-index: GEN 0.589, MRI 0.727, CDC 0.822, combined up to 0.831	Nonlinear survival analysis improves on Cox model	Small sample, MRI features from FreeSurfer only
Cirincione et al. (2024)	Imaging (sMRI, PET) + Cognitive + Biomarkers + Demographic + Genetic	Ensemble Integration	TADPOLE (ADNI-based)	AUC: EI=0.81, XGBoost=0.68, DL=0.79; F-measure: EI=0.68	Effective multimodal integration	Lack of longitudinal data integration
Aqil et al. (2024)	Imaging (MRI, PET) + Cognitive + CSF + Demographic + Genetic (APOE4)	Temporal Graph Neural Network (TGN)	ADNI	AUC: 0.8090 (all features), 0.8807 (neuroimaging only)	Captures temporal dynamics of AD progression	High computational resource needs
Prakash et al. (2021)	Imaging (MRI, AV-45 PET, FDG-PET) + Cognitive + CSF + Demographic + Genetic (APOE $\varepsilon$4)	PLSR, SVR, RF	ADNI	MAE (12/24/36 mo): 4.1/4.5/5.0, $\rho$: 0.88/0.82/0.75; improved with baseline ADAS-cog	Multi-modal data improves prediction accuracy	Prediction accuracy declines over time
El-Sappagh et al. (2022b)	Imaging (T1 MRI) + Cognitive + CSF + Genetic + Neuropathology + Sociodemographic	ML & DL Models	ADNI	Classification: Acc 93.87%, Precision 94.07%, Recall 94.07%, F1 94.07%	Innovative two-stage framework	Lack of explainability, advanced tech reliance

The Sequence-Length Adaptive Encoder-Decoder LSTM (SLA-ED LSTM) model predicts AD progression using longitudinal multimodal data, including MRI, PET, cognitive assessments, and genetic factors. Achieving an accuracy of 0.920 and an AUC-ROC of 0.982, the model dynamically adapts to variations in sequence length and visit frequency, outperforming existing methods. However, the study suggests improving local and global interpretability to enhance clinical applicability (Poonam et al. 2024). DISFC, a deep neural network (DNN) model (Wang et al. 2024), predicts MCI to AD progression by incorporating structural MRI, clinical assessments, and genetic SNP biomarkers. Trained on ADNI, it achieves 92.86% accuracy and an AUC of 0.939. By capturing interaction effects among multimodal data, DISFC enhances early intervention strategies for AD. A study (Franciotti et al. 2023) evaluating Random Forest (RF), Gradient Boosting (GB), and eXtreme Gradient Boosting (XGB) for MCI-to-AD conversion prediction integrates structural MRI, CSF biomarkers (p-Tau, A$\beta$42, p-Tau/A$\beta$42), and genetic data (APOE $\varepsilon 4$). The model reaches 90% accuracy when incorporating neuropsychological and AD biomarkers, with PPV of 0.91 (GB & XGB), NPV of 0.87 (RF), sensitivity of 0.67 (sMRI), and specificity of 0.97 (GB & XGB with AD biomarkers). A feature selection and voting procedure enhanced prediction reliability, though GB and XGB models showed signs of overfitting. The PPAD and PPAD-Autoencoder models, based on recurrent neural networks (RNNs), predict MCI-to-AD conversion using MRI, demographic, cognitive, and genetic data. Trained on ADNI datasets (ADNI1, ADNI2, ADNI-GO) and tested on NACC, the models leverage age to handle irregular time intervals in longitudinal data. They outperform baseline models in F2 score and sensitivity, though generalizability across different cohorts remains a challenge (Al Olaimat et al. 2023).

A hybrid machine learning framework (Wang et al. 2022) integrates CNNs for image feature extraction with an SVC for classification, utilizing T1 MRI, cognitive, genetic (APOE), and clinical (demographic) data. The model achieves high accuracy (0.88 ADNI, 0.84 J-ADNI) and strong AUC scores (0.95 ADNI, 0.91 J-ADNI), demonstrating robust generalizability. However, its limited dataset size and reliance on baseline data constrain its potential. A parameter-efficient 3D CNN with multi-task learning (MTL) predicts AD progression using structural MRI, clinical (cognitive and demographic), and genetic (APOE $\varepsilon 4$) data. The model achieves high accuracy (86%), sensitivity (87.5%), specificity (85%), and an AUC of 0.925 for MCI-to-AD conversion. Additionally, it perfectly classifies AD vs. healthy controls (AUC: 1). Despite its effectiveness, the model is limited by its use of baseline data and lack of advanced biomarkers like PET and CSF (Spasov et al. 2019). A multi-modal deep learning model (RNN + Multi-modal GRU) predicts MCI to AD conversion by integrating MRI, PET, CSF biomarkers, and genetic data (APOE) with diagnostic information. The model achieves 81% accuracy and an AUC of 0.86, demonstrating potential for identifying at-risk individuals. However, its applicability may be restricted to the ADNI dataset, limiting transferability to other populations (Lee et al. 2019). A unified machine learning model (Nie et al. 2017) employs Multisource Multitask Learning (MML) to integrate MRI, PET, CSF biomarkers, demographic data (age, education, gender), and genetic data (APOE $\varepsilon 4$). The model achieves strong performance on MMSE (R-value: 0.8794, nMSE: 0.1233) and ADAS-Cog (R-value: 0.9094, nMSE: 0.0347). By incorporating matrix factorization to address missing data, the model is validated on real-world Alzheimer’s datasets, enhancing its predictive power.Lastly, a Gaussian process-based method (Young et al. 2013) predicts MCI-to-AD conversion using MRI, FDG-PET, CSF biomarkers, and genetic data (APOE). The method achieves 74% accuracy and an AUC of 0.795, offering probabilistic predictions that surpass unimodal approaches. However, its relatively small sample size (96 MCI-stable and 47 MCI-converters) limits generalizability, necessitating larger datasets for broader application.

One study (Mirabnahrazam et al. 2023) applies a DeepSurv survival model using multimodal baseline data from the ADNI dataset to predict the time-to-conversion to Dementia of Alzheimer’s Type (DAT). The model incorporates imaging (MRI), clinical assessments (such as MMSE cognitive tests and CSF biomarkers), demographic data, and genetic information (SNP, APOE). The findings reveal that clinical, cognitive, and demographic data (CDC) are the most predictive for MCI subjects, whereas genetic factors (SNP, APOE) hold greater predictive value for individuals with normal cognition. Moreover, combining MRI with genetic data enhances prediction accuracy. The model outperforms the traditional Cox model, achieving a Ctd-index of 0.831 for the combined MRI and CDC data and 0.800 for all modalities together. However, limitations include the small ADNI sample size, sensitivity of the Permutation Importance method, and reliance on FreeSurfer-derived MRI features instead of voxel-level deep learning.

Another study (Cirincione et al. 2024) introduces an Ensemble Integration (EI) machine learning framework designed to predict future dementia in MCI patients using multimodal data, including imaging (sMRI, PET), cognitive test scores (ADAS 13, CDR-SB, MMSE), CSF biomarkers, demographic details, and genetic data (APOE4). The framework outperforms traditional methods, achieving an AUC of 0.81, surpassing XGBoost (0.68) and deep learning models (0.79). It also demonstrates a higher F-measure of 0.68 compared to 0.57 for XGBoost and 0.61 for deep learning. Notably, the EI model identifies key MRI-derived brain volume metrics associated with dementia progression. However, a limitation of this study is the lack of longitudinal data integration. The dataset originates from the TADPOLE Challenge, based on ADNI 1, 2, and GO studies.

A different study (Aqil et al. 2024) proposes a temporal graph neural network (TGN) model for predicting AD progression, leveraging multimodal data such as imaging (MRI, PET), cognitive tests (CDRSB, ADAS11/ADAS13, MMSE, RAVLT, FAQ, MOCA, EcogPt/EcogSP), CSF biomarkers (ABETA, TAU, PTAU), demographic details, and genetic information (APOE4). The TGN model captures the temporal dynamics of disease progression by employing dynamic edge prediction. The approach achieves an AUC of 0.8090 when utilizing all patient features, with a remarkable AUC of 0.8807 when using only neuroimaging data. These results highlight the model’s effectiveness in capturing temporal dependencies and its high predictive accuracy, especially when relying on neuroimaging data. However, the study acknowledges that graph-based methods demand substantial computational resources, which may hinder real-time implementation. Nevertheless, the TGN model presents a promising avenue for personalized disease monitoring by integrating temporal information into AD progression prediction. Another study (Prakash et al. 2021) investigates multi-modal predictive learning models to forecast AD progression over 12, 24, and 36 months using ADAS-cog scores. The models combine imaging data (MRI, AV-45 PET, FDG-PET), cognitive tests (ADAS-cog 13, MMSE, RAVLT, FAQ), CSF biomarkers (A$\beta$42, t-tau, p-tau), demographic variables (age, education), and genetic information (APOE $\varepsilon 4$). The predictive frameworks, comprising Partial Least Squares Regression (PLSR), Support Vector Regression (SVR), and Random Forest (RF), consistently outperform single-modal models. They achieve Mean Absolute Errors (MAEs) of 4.1, 4.5, and 5.0 and correlation coefficients ($\rho$) of 0.88, 0.82, and 0.75 for 12, 24, and 36 months, respectively. When incorporating baseline ADAS-cog scores, the MAEs further improve to 3.5, 3.7, and 4.6, with correlation coefficients of 0.89, 0.87, and 0.80. However, the study notes a decline in prediction accuracy over time, as correlations decrease with disease progression, emphasizing the challenges of maintaining long-term prediction reliability. This approach underscores the significance of multimodal data for early identification of high-risk individuals, offering valuable support for personalized treatment planning and clinical trial design.

Finally, a study (El-Sappagh et al. 2022b) presents a two-stage deep learning framework designed to classify AD progression and predict the conversion time of MCI patients. This model, trained on multimodal ADNI data, including MRI, cognitive scores, CSF biomarkers, genetic information (APOE $\varepsilon$4), and sociodemographic details, demonstrates superior performance compared to traditional machine learning approaches. The results show high classification accuracy (93.87%), precision (94.07%), recall (94.07%), and F1-score (94.07%), along with a low Mean Absolute Error (MAE) of 0.1375. Despite its impressive predictive capability, the study highlights two major challenges: the lack of model explainability and reliance on advanced imaging technologies like MRI and PET, which may limit its practical application in resource-constrained settings. Nonetheless, this deep learning model offers significant promise for early AD detection and progression monitoring.

These studies illustrate the potential of integrating imaging, clinical, and genetic data to improve AD progression prediction. By leveraging advanced machine learning techniques and multimodal data fusion, these approaches enhance predictive accuracy, disease staging, and early intervention strategies. However, challenges such as overfitting, dataset limitations, and the need for improved interpretability remain key areas for future research.

#### Layer 3: Therapeutic Discovery

Therapeutic discovery for Alzheimer’s disease (AD) has gained significant traction with the advancement of machine learning, artificial intelligence, and deep learning approaches. These technologies offer promising solutions for drug repositioning, therapeutic target identification, and drug discovery. By leveraging existing drug data, protein interaction networks, and AI-powered generative models, these studies aim to accelerate the discovery of effective treatments for AD. Despite the progress, challenges such as data incompleteness, model validation, and drug efficacy need to be addressed. The following studies explore different AI-driven methodologies for therapeutic discovery in Alzheimer’s disease, highlighting the potential for novel treatments.

DOTA (Deep learning-based Drug Repositioning for Alzheimer’s) is a deep learning-based drug repositioning method (Chyr et al. 2022) that uses optimal transport and autoencoders to identify FDA-approved drugs with potential effectiveness in treating AD. By integrating heterogeneous drug data, DOTA predicts promising candidates, such as antipsychotic drugs like quetiapine, aripiprazole, and risperidone, which target key brain receptors involved in memory and cognition. While the study faces limitations, including incomplete drug networks and challenges in model evaluation, DOTA offers a powerful framework for identifying novel drug-disease associations and can be applied to other diseases as well, providing significant clinical impact. A network-based AI framework (Fang et al. 2022a) was developed to integrate multi-omics data and human protein–protein interactomes to identify AD risk genes (ARGs) and potential drug targets. The study led to the identification of 103 ARGs, revealing that pioglitazone (a drug for type 2 diabetes), febuxostat, and atenolol were associated with a decreased risk of AD. Notably, pioglitazone was found to significantly reduce AD risk, supported by in vitro evidence demonstrating its effect on key AD-related proteins in human microglia cells. This network-based approach offers a promising route for therapeutic discovery and advancing Alzheimer’s drug development. This study (Tsuji et al. 2021) presents a deep learning-based framework designed to prioritize novel therapeutic targets by leveraging the human protein-protein interaction network (PIN). The framework extracts low-dimensional representations of high-dimensional PIN data to identify potential drug target genes. Applied to AD, the model identified key genes (such as DLG4, EGFR, and RAC1) and suggested repositionable drugs (e.g., tamoxifen and bosutinib). This computational approach offers an efficient method for discovering new therapeutic targets, supporting the drug repositioning process in Alzheimer’s disease. Generative AI (GAI) technologies, such as ChatGPT, were explored for identifying drug repurposing candidates for AD. By asking ChatGPT to propose promising drugs, the researchers tested the top candidates in large clinical datasets and found that drugs like metformin, simvastatin, and losartan were associated with a lower risk of AD. While this approach shows promise, the study notes the challenges in establishing causal effects and the limitations arising from data completeness. Nevertheless, generative AI tools like ChatGPT present a valuable framework for generating hypotheses and streamlining drug discovery for AD and other conditions (Yan et al. 2024). The DRIAD (Drug Repurposing in AD) framework (Rodriguez et al. 2021) is a machine learning model designed to identify potential FDA-approved drugs for repurposing in Alzheimer’s disease. By analyzing gene expression data from human neural cells treated with 80 FDA-approved drugs, DRIAD ranks these drugs based on their association with AD pathology, as defined by Braak staging. The framework identifies drugs that target pathways involved in immunity, autophagy, and microtubule dynamics, offering new therapeutic avenues for AD. While promising, the method requires further validation and consideration of factors like blood-brain barrier penetration and the relevance of drug mechanisms.

The studies discussed illustrate the transformative potential of AI, deep learning, and generative models in therapeutic discovery for AD. By repurposing existing drugs and identifying novel drug targets, these approaches help expedite the development of effective treatments. However, challenges like data incompleteness, causal inference limitations, and the need for clinical validation persist. Despite these hurdles, these innovative frameworks open up new avenues for drug repositioning and provide a stepping stone for further research aimed at tackling Alzheimer’s disease.

***Bridging AI-Driven Discovery with the Clinical Pipeline***

While AI-driven frameworks are exceptionally powerful for generating therapeutic hypotheses and identifying repurposing candidates, their output is the starting point of a long and arduous validation process. The drugs identified in the aforementioned studies—ranging from antipsychotics to metabolic drugs—occupy various stages of the clinical development pipeline for Alzheimer’s disease (AD), with a mixed record of success. Evaluating their current status is crucial for a complete understanding of AI’s real-world impact.

A prominent example is pioglitazone, an anti-diabetic drug identified by a network-based AI framework. The strong mechanistic link between insulin resistance and AD pathology made it a promising candidate. This led to a large-scale Phase 3 clinical trial (TOMORROW) (Burns et al. 2021), which aimed to see if pioglitazone could prevent or delay the onset of mild cognitive impairment (MCI) in at-risk individuals. Unfortunately, the trial was halted for futility, highlighting that even a strong, AI-supported hypothesis does not guarantee clinical success. Similarly, the antihypertensive drug losartan (Kehoe et al. 2021) and the cholesterol-lowering drug simvastatin (Mejías-Trueba et al. 2018) have been tested in late-stage trials for AD with largely disappointing results in terms of modifying the disease course.

Other AI-identified candidates are in earlier stages of investigation. Bosutinib (Mahdavi et al. 2021), a leukemia drug, is a compelling example. It has been shown in preclinical models to promote the clearance of pathological proteins like tau. This has led to early-phase clinical trials to test its safety, brain penetration, and target engagement in patients with neurodegenerative diseases, representing a successful transition from a computational prediction to human studies. Drugs like metformin (Tahmi and Luchsinger 2023) are also under intense investigation, with ongoing trials like the MEMOIR study specifically testing its efficacy in AD patients, fueled by both AI-driven hypotheses and extensive epidemiological data.

Finally, some of the identified drugs, such as the antipsychotics (Maust et al. 2015) quetiapine and risperidone, are already used in clinical practice for AD patients. However, their use is not for disease modification but for managing behavioral symptoms like psychosis and agitation. Their repurposing in this context is for symptomatic relief and is often accompanied by significant safety concerns (e.g., increased mortality risk), which AI models focused solely on disease mechanisms may not initially consider.

This translational gap underscores a critical challenge: AI models excel at identifying statistical and network-based associations, but the biological complexity of AD, issues with blood-brain barrier penetration, and unforeseen off-target effects in patients require rigorous, multi-phase clinical validation. Therefore, the true value of these AI discovery platforms lies in their ability to enrich the pipeline with higher-quality candidates, potentially increasing the success rate of the costly and time-consuming journey from lab to clinic.

#### Layer 4: Real-world Integration (Patient Care)

Advancements in artificial intelligence, wearable technology, and conversational AI have led to innovative solutions for improving the quality of life for Alzheimer’s patients and their caregivers. These technologies assist with daily tasks, enhance safety, and provide emotional and medical support. However, despite their potential, very few studies have explored AI-driven solutions specifically for patient care in AD. The following studies present diverse approaches, including reinforcement learning-based reminder agents, AI-driven digital assistants, IoT-integrated monitoring systems, and computer vision models for pain detection, all aimed at optimizing patient care and easing caregiver burden.

An AI-powered system (Shalini et al. 2024) integrates IoT and GPS technologies to enhance Alzheimer’s patient care, offering features such as a GPS-enabled tracker for locating essentials, a smart IoT-based medicine dispenser with automated alerts, and AI-powered virtual assistants for reminders and communication support. The system also employs health monitoring, wearable technology, and machine learning for behavior analysis. A mobile application centralizes these functionalities, ensuring round-the-clock assistance for both patients and caregivers. The proposed solution aims to improve patient independence and streamline healthcare management. ADQueryAid, a conversational AI system, supports caregivers of individuals with Alzheimer’s disease and related dementias (ADRD) by leveraging a Large Language Model with Retrieval-Augmented Generation. By integrating patient information and medical knowledge, it provides personalized and contextually relevant support. Key features include an intuitive interface, educational resources, and goal-oriented assistance. To ensure reliability, responses are grounded in peer-reviewed literature and official guidelines. A user study found ADQueryAid superior to ChatGPT 3.5 in providing emotional and practical caregiving support, demonstrating the potential of tailored AI in enhancing caregiving experiences (Hasan et al. 2024). MemoryCompanion (Zheng et al. 2023), an AI-driven digital health solution, employs GPT-based language models, voice cloning, and talking-face technology to create personalized interactions for Alzheimer’s patients. It features a patient-centric language model for memory retrieval, a structured patient profile system, and custom evaluation metrics for smart caregiving. The system enhances engagement and combats social isolation but faces challenges related to ethical concerns, data privacy, and the need for more immersive interactions. Future research will focus on ethical safeguards, data protection, and advanced multimedia experiences such as augmented reality (AR) to improve patient care. A wearable AI-powered prototype (Chokri et al. 2022) supports Alzheimer’s patients by integrating facial recognition, location tracking, and psychological monitoring. Using a CNN-based model, it classifies detected faces as family or non-family members, with steganography protecting identities and providing contextual information. The IoT-enabled device tracks patient location, sending alerts if they leave a designated safe zone. Google Assistant integration facilitates voice interactions, reducing social isolation while assessing psychological well-being. Secure communication is ensured through S/MIME encryption. Future enhancements will explore thermal imaging, 3D facial reconstruction, and improved voice integration for enhanced recognition and usability. A fully automated computer vision model (Rezaei et al. 2021) addresses the challenge of detecting pain in non-verbal dementia patients. By analyzing facial expressions with deep learning, the system applies a pairwise comparative method to detect expression changes and uses contrastive training to improve generalization. The model significantly outperforms existing pain assessment tools, particularly for dementia patients. However, challenges remain due to limited and imbalanced datasets. Future research will explore unsupervised training methods to enhance performance and overcome data constraints in clinical settings. A reinforcement learning-based reminder agent (Chen and Soh 2018) assists Alzheimer’s patients with cooking tasks, modeling the cooking process as a Markov Decision Process (MDP) with continuous state and action spaces. The system optimally times reminders to help patients complete subtasks, using policy gradient methods and function approximation to handle the complex MDP. The study introduces a novel MDP formulation for cooking assistance and applies inverse reinforcement learning to improve task completion. Future work will extend this to a Partially Observable MDP (POMDP) to account for unobservable patient behaviors and real-world testing.

These studies highlight the transformative role of AI, IoT, and machine learning in Alzheimer’s patient care. From task reminders and caregiver assistance to safety monitoring and pain detection, these innovations aim to improve patient well-being, foster independence, and reduce caregiver burden. However, research in this area remains limited, with most studies focusing on disease diagnosis and progression rather than direct patient assistance. The challenges of data limitations, ethical concerns, and privacy issues further emphasize the need for more research and real-world validation to optimize these technologies for broader adoption.

### Trends and insights

#### Explainable AI in Alzheimer’s research

**Fig. 10:**
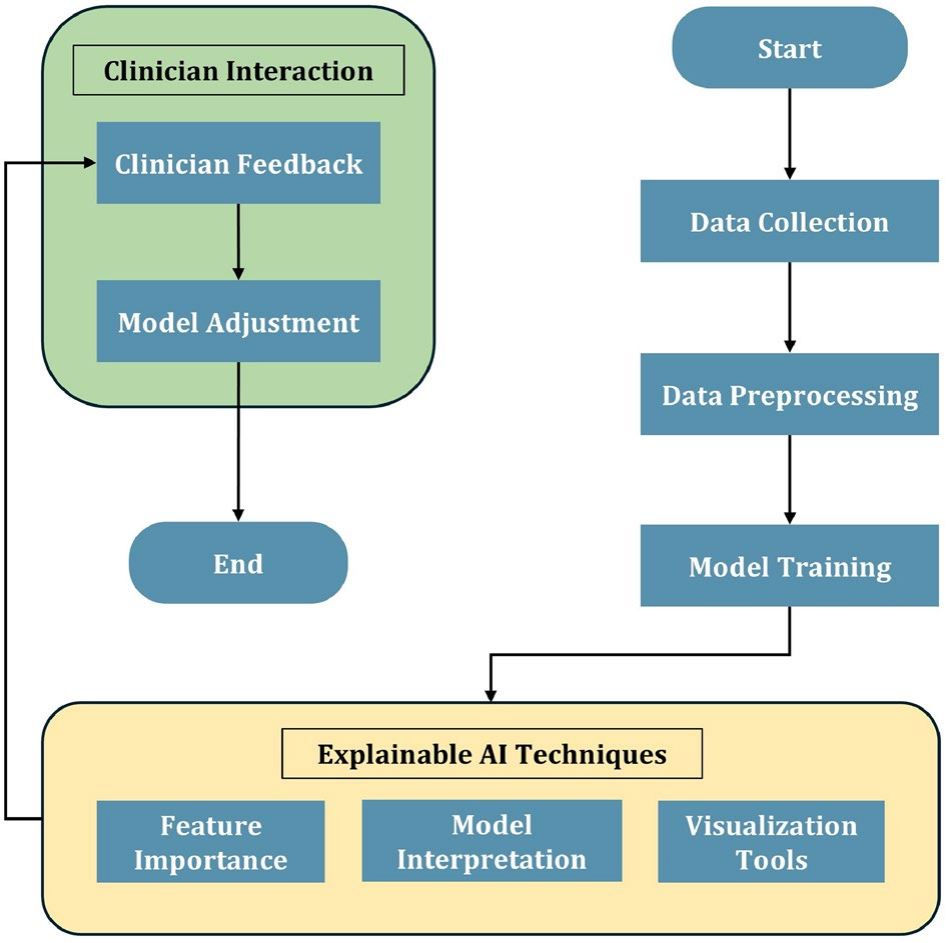
Role of explainable AI in Alzheimer’s research

In recent years, the integration of Explainable AI (XAI) techniques has become a significant trend in Alzheimer’s disease (AD) research, particularly in enhancing the interpretability and trustworthiness of AI-driven diagnostic models. As AI systems are increasingly used to support medical decision-making, their transparency is crucial in ensuring that clinicians can rely on them to make informed decisions. Several studies have explored different XAI methods to provide insights into the decision-making processes of AI models, making them more accessible and understandable to healthcare professionals. Figure 10 illustrates the integration of explainable AI (XAI) techniques into the Alzheimer’s research pipeline. After standard steps such as data collection, preprocessing, and model training, XAI methods, such as feature importance, model interpretation, and visualization tools, are applied to enhance transparency. These insights facilitate clinician interaction, allowing for feedback that guides model adjustment and refinement. This iterative loop ensures that AI models are not only accurate but also interpretable and clinically relevant, ultimately supporting informed decision-making in Alzheimer’s diagnosis and care.

One approach (Jain et al. 2021) uses GradCAM-based visualizations to interpret AI model decisions in the context of dementia diagnosis. The D-BAC model, which employs CNN architectures for classifying dementia severity from MRI scans, incorporates GradCAM to highlight key regions in the brain images that influence the model’s predictions. This method allows medical practitioners to visually assess which areas of the brain are contributing to the AI’s diagnosis, enhancing its transparency and making it easier for clinicians to trust AI-driven diagnostics. Another noteworthy development (Yu et al. 2022) in XAI for AD detection is the MAXNet model, which introduces High-resolution Activation Mapping (HAM) and a Prediction-basis Creation and Retrieval (PCR) module. HAM allows for the precise localization of disease-related brain areas, while PCR provides visual evidence by retrieving similar brain scans. This dual approach not only improves diagnostic accuracy but also facilitates clear, high-resolution insights into the AI model’s reasoning, making it easier for healthcare professionals to interpret the predictions.

Similarly, the SECNN-RF framework (AbdelAziz et al. 2024) uses Squeeze-and-Excitation (SE) blocks to enhance interpretability by dynamically prioritizing critical features in MRI scans. Unlike traditional CNNs, this model employs a Random Forest classifier to improve transparency, isolating feature extraction from decision-making. Saliency maps further support clinical interpretation by visually explaining the model’s classification decisions, ensuring that its predictions are both accurate and understandable. In another innovative study (Shojaei et al. 2023), a hybrid method combines Backpropagation-based explainability techniques with an Occlusion Map optimized using a genetic algorithm. This 3D-CNN model, trained on MRI scans, identifies the most informative brain regions linked to Alzheimer’s disease, achieving a remarkable accuracy of 93%. By refining the brain mask using a genetic algorithm, the model enhances interpretability while maintaining diagnostic performance, providing clinicians with clearer insights into the disease’s progression. The IADT model (Guan et al. 2023) integrates domain transfer learning with an attention mechanism to predict the progression from subjective cognitive decline (SCD) to mild cognitive impairment (MCI) and Alzheimer’s disease (AD). By automatically identifying disease-related brain regions, it provides clinicians with direct interpretability, which is crucial for making informed decisions. This approach not only improves feature learning but also maintains the model’s transparency, ensuring that the predictions are grounded in clinically meaningful biomarkers.

A multimodal framework (Parvin et al. 2024) for AD classification takes advantage of various XAI methods, including Layer-wise Relevance Propagation (LRP) for interpreting MRI scans, Submodular Pick Local Interpretable Model-Agnostic Explanations (SP-LIME) for understanding tabular data features, and Graphical Gene Tree (GGT) analysis to identify relevant genes. This combination of techniques promotes collaboration between researchers and clinicians, providing clear, interpretable predictions and ultimately improving patient outcomes by facilitating more personalized treatment plans. Explainable Boosting Machines (EBMs) are also gaining traction in AD research for predicting the conversion of mild cognitive impairment (MCI) to Alzheimer’s disease. By utilizing multimodal data, including MRI biomarkers and clinical features, EBMs offer both global and local explanations, enabling clinicians to track the progression of AD. The model’s feature importance ranking and interaction effect analysis provide transparent insights into the underlying factors contributing to the disease, thereby increasing the model’s reliability in clinical settings (Cai et al. 2023). In the realm of Graph Convolutional Networks (GCN), a novel decomposition-based explanation method (Tekkesinoglu and Pudas 2023) for AD diagnosis has been proposed. This method evaluates feature importance at individual, group, and neighborhood levels, providing both factual and counterfactual explanations. This approach improves the interpretability of the model, ensuring that clinicians can trust the AI’s predictions while addressing concerns regarding its reliance on demographic and neuroimaging data.

Another study (Aghaei et al. 2022) addresses the challenge of early Alzheimer’s detection by employing Local Interpretable Model-agnostic Explanations (LIME) to highlight the brain regions influencing the AI model’s decisions. Evaluated by expert radiologists, this method confirmed significant alignment with clinical insights, making the model more reliable and interpretable. The use of LIME enhances the decision-making process, fostering trust in AI applications within clinical environments. In longitudinal studies, heatmaps have been used to improve the interpretability of deep learning models predicting the conversion of MCI to AD. By focusing on critical brain regions, these heatmaps enhance the model’s transparency, making it easier for clinicians to understand and trust the predictions. Future work aims to refine feature extraction methods and incorporate additional modalities to improve both accuracy and interpretability (Bapat et al. 2024). The integration of 3D CNNs and bidirectional recurrent neural networks (BRNN) in AD diagnosis (Rahim et al. 2023) has led to the development of a novel approach for detecting AD progression using longitudinal MRI data. This model not only integrates spatial and temporal features but also incorporates 3D visual attention maps, which help medical experts track progressive patterns in the brain over time. This approach aligns with expert diagnoses, reinforcing the trustworthiness of AI-based predictions.

A new biomarker, the Deep Grading (DG) method (Nguyen et al. 2023), generates 3D grading maps to quantify disease severity, allowing clinicians to localize abnormal brain regions with precision. This quantitative approach offers an alternative to traditional qualitative explanation methods, combining interpretability with high classification performance. The DG biomarker enhances both diagnosis and prognosis, making it an effective feature for predicting AD progression. Another study (Chen et al. 2025) introduces a unique latent multimodal deep learning framework for predicting AD cognitive status, with a strong emphasis on explainability through attention mechanisms. By incorporating attention and cross-attention layers, the model not only improves prediction accuracy but also reveals modality importance scores, highlighting how different data types, clinical, imaging, and genetic, contribute to the outcome. This approach enhances model transparency and interpretability, allowing for clear insights into which features drive predictions, a key advancement in explainable AI for neurodegenerative disease diagnosis. Finally, the use of a Vision Transformer (ViT)-Gated Recurrent Unit (GRU) model (Mahim et al. 2024) in AD diagnosis combines multiple XAI techniques, including LIME, SHAP, and attention maps. By providing interpretable insights into the model’s predictions, this framework helps clinicians understand the affected brain regions, such as hippocampal atrophy and cortical degeneration. This transparency fosters greater trust in AI-driven diagnosis, paving the way for real-world clinical adoption.

These advancements in XAI represent a major shift in AI-driven Alzheimer’s research, moving beyond black-box models toward more interpretable, trustworthy systems. While significant progress has been made, challenges remain, particularly in standardizing XAI techniques across different data modalities and improving real-time usability in clinical settings. Future research should focus on integrating multiple XAI methods, validating models across diverse patient populations, and refining user-friendly interpretability tools that can be seamlessly adopted by medical practitioners. By addressing these challenges, explainable AI has the potential to transform precision neurology, enabling early detection and personalized intervention strategies for AD.

#### Federated learning in Alzheimer’s research

Federated learning has emerged as a transformative approach in AD research, addressing critical challenges related to data privacy, security, and scalability. Traditional machine learning models often require centralized datasets, which can raise concerns about patient confidentiality. Federated learning, on the other hand, enables decentralized training, allowing models to learn from distributed clinical data without exposing sensitive patient information. This approach not only enhances data security but also ensures that AI models are trained on diverse datasets from multiple sources, leading to improved generalizability and robustness in AD diagnosis. Several studies have explored federated learning for AD detection, integrating deep learning techniques to enhance classification accuracy while maintaining strict privacy measures.

**Fig. 11:**
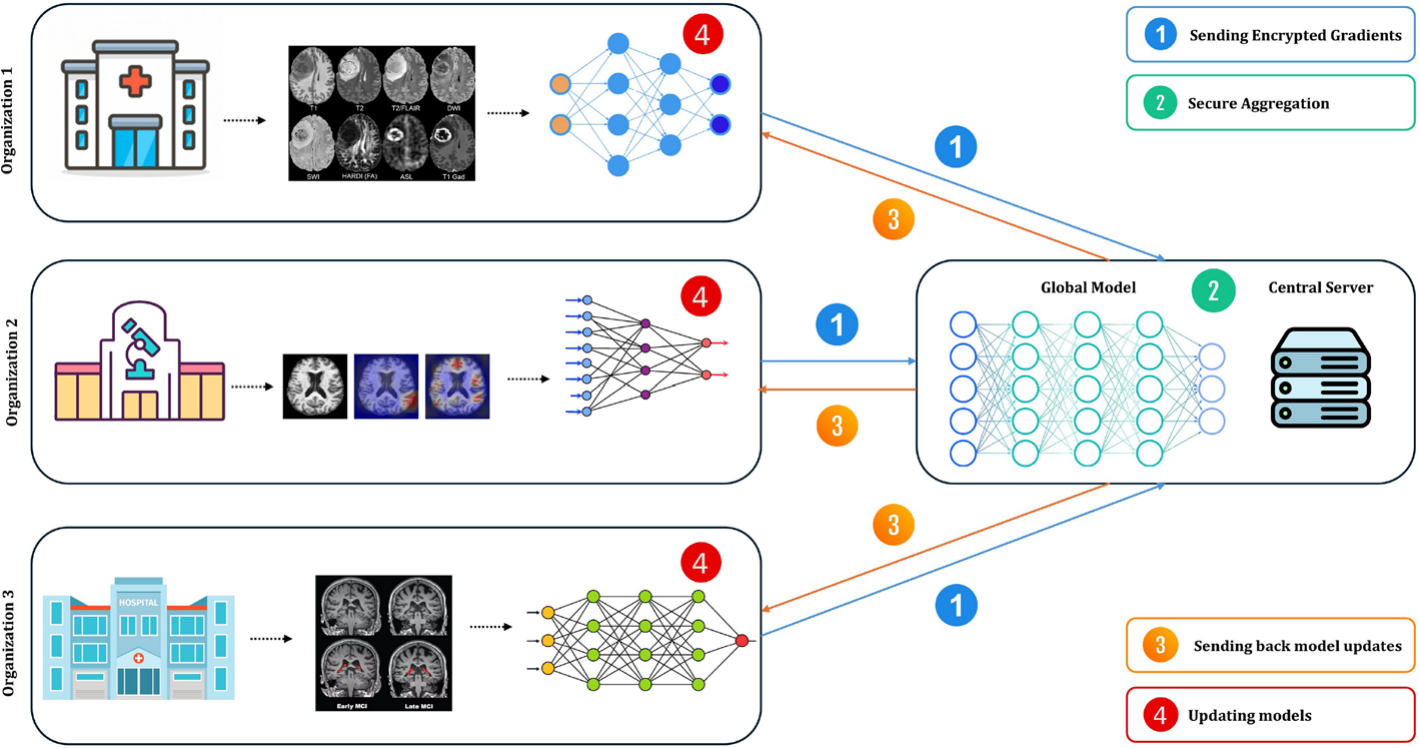
Federated learning framework

Figure 11 illustrates a federated learning framework in a healthcare setting, where multiple organizations (hospitals) collaboratively train a shared global machine learning model without sharing their private medical data. Each organization uses its local MRI brain scan data to train individual neural network models and then sends encrypted gradients to a central server (Step 1). The server performs secure aggregation of these encrypted updates to improve the global model (Step 2), which is then sent back to the organizations as updated model parameters (Step 3). Each organization uses these updates to refine its local model (Step 4). This approach ensures data privacy and security while enabling collaborative model development for applications such as disease diagnosis from medical imaging.

One study (Mandawkar and Diwan 2022) proposes a federated learning-based deep CNN classifier, the Tawny Flamingo model, designed specifically for AD detection. By enabling distributed learning across multiple clinical data sources, this model ensures that patient data remains private while still contributing to an effective AI training process. The Tawny Flamingo algorithm optimizes deep CNN parameters, improving model performance. Although federated learning strengthens data security, the study highlights the need for further research on detecting malicious attacks, which could compromise the integrity of decentralized learning environments. Another study (Lakhan et al. 2023) introduces the Evolutionary Deep Convolutional Neural Network Scheme (EDCNNS), which enhances the accuracy and efficiency of AD detection through federated learning. This method securely trains AI models on patient data from various laboratories without centralizing sensitive information. An aggregation scheme ensures efficient processing of distributed data while maintaining privacy. The model also leverages fog and cloud computing to optimize decision-making, reducing computational overhead while improving detection accuracy. While federated learning minimizes the computational burden and enhances security, future research could focus on refining resource allocation and developing strategies to mitigate adversarial attacks in federated environments. Building on these advancements (Lakhan et al. 2024), a Federated Deep Convolutional Neural Network Alzheimer Detection Scheme (FDCNN-AS) has been developed to securely train and test AD datasets across multiple healthcare clinics. This approach integrates diverse data sources, including MRI scans, CT scans, blood biomarkers, and questionnaires, to improve AD classification without exposing raw patient data. Local deep CNN models are trained at individual clinical nodes, and their results are aggregated to enhance detection accuracy. The model also incorporates encryption layers to protect dataset weights during transmission, ensuring secure collaboration between distributed healthcare institutions. Future developments aim to expand the federated learning network to a larger number of hospitals and incorporate blockchain technology for secure interoperability and drug recommendation systems. The proposed model in this study (Jahan et al. 2025) combines Random Forest (RF) classifiers with multimodal data (MRI scans, clinical, and psychological data) from the OASIS-3 dataset, achieving high prediction accuracy (98.93%). FL ensures that sensitive health data remains localized, while still benefiting from cross-institutional insights, making the model both privacy-preserving and scalable. The system (Basnin et al. 2025) uses a CNN to analyze MRI images and combines predictions with demographic data for local model training. A belief rule base (BRB) is integrated into the FL framework to effectively manage uncertainty in medical data. Among the evaluated aggregation methods (FedAvg, FedProx, Genetic Algorithm), FedAvg achieved the highest global accuracy (99.99%). Despite some challenges with communication efficiency and heterogeneous data, the approach shows strong potential for privacy-preserving, reliable, and scalable AD diagnosis. Further pushing the boundaries of federated learning, a Hybrid Federated Learning (HFL) framework (Lei et al. 2024b) has been introduced for AD detection, emphasizing both privacy preservation and the utilization of unlabeled structural MRI (sMRI) data. Unlike conventional federated learning methods, HFL enables collaborative training across multiple medical centers while ensuring strict data privacy. The framework incorporates a Brain-region Attention Network (BANet), which identifies key regions of interest in brain scans to improve classification accuracy. Additionally, a self-supervised loss function enhances the model’s ability to learn meaningful representations from unlabeled data, maximizing the utility of available datasets. Experimental results demonstrate that HFL-BANet outperforms existing models, making it a promising approach for multi-center AD research. Future directions include integrating multi-modal imaging data, such as fMRI and PET, and optimizing preprocessing techniques to further improve detection accuracy while maintaining the privacy-preserving benefits of federated learning.

These studies collectively highlight the growing role of federated learning in AD research, offering a secure, decentralized approach to AI model training. By ensuring patient confidentiality while leveraging large-scale, diverse datasets, federated learning is paving the way for more accurate and reliable AI-driven diagnostics in AD. Future research will likely focus on refining federated architectures, improving attack detection mechanisms, and integrating additional medical data modalities to enhance model performance and trustworthiness.

#### Foundation models in Alzheimer’s research

Foundation models have emerged as a powerful tool in Alzheimer’s Disease (AD) research, particularly with their ability to leverage pre-trained architectures to enhance diagnostic accuracy and efficiency. These models, often built on large-scale datasets, are fine-tuned for specific tasks, allowing researchers to build on the vast knowledge encoded in models like Vision Transformers (ViTs) and other multimodal frameworks. This ability to adapt to a wide range of data types, such as images, speech, and clinical information, makes foundation models particularly valuable in AD diagnosis. Several studies have explored their potential in this domain, demonstrating significant improvements in both performance and interpretability.

One study (Menon and Gunasundari 2024) explores a fusion model that combines DenseNet-121 and Vision Transformer (ViT) for AD classification from MRI images. By integrating DenseNet’s local feature extraction capabilities with ViT’s global attention mechanisms, the model achieves state-of-the-art performance with 99% accuracy. Although the study does not explicitly refer to foundation models, it utilizes pre-trained models, aligning with the foundation model paradigm. Future research could scale this approach with larger foundation models, potentially improving generalization across diverse datasets and enhancing explainability through methods like Class Activation Maps (CAMs). Another study (Ilias et al. 2023) examines the use of pre-trained Vision Transformers (ViT) and other computer vision models for classifying AD from speech data. This innovative approach converts speech into log-Mel spectrograms and MFCCs, allowing the use of models originally designed for image data. The integration of both speech and transcripts through a Gated Multimodal Unit and crossmodal attention significantly improves classification, achieving 88.83% accuracy, surpassing traditional methods. This study demonstrates the versatility of foundation models in handling multimodal data, paving the way for further optimization in speech-text fusion and representation learning. The ADQueryAid conversational AI system, leveraging a Large Language Model with Retrieval-Augmented Generation, also exemplifies the power of foundation models in AD research. Designed to support caregivers of individuals with AD and related dementias (ADRD), this AI system integrates patient information and medical knowledge to provide personalized, contextually relevant support.

A user study found ADQueryAid superior to ChatGPT 3.5, particularly in providing emotional and practical caregiving support. This system highlights the potential of tailored AI in enhancing caregiving experiences and provides a new avenue for leveraging foundation models in healthcare (Hasan et al. 2024). In another study (Shaffi et al. 2024), Vision Transformers (VTs) are explored for AD classification, with pre-trained transformer models like Swin, DeiT, BEiT, and ViT compared both individually and in ensembles. The ensemble model outperforms traditional CNNs and machine learning approaches, achieving an impressive 99.29% accuracy. The study emphasizes VTs’ ability to capture long-range dependencies, adapt to data scarcity, and handle class imbalance, key strengths of foundation models. The research suggests future directions, including enhancing interpretability and exploring multimodal data integration to further improve AD diagnosis. A foundation model-based multi-modal multi-scale Transformer network (MMTFN) is introduced in another study to diagnose AD, integrating MRI and PET scans using 3D Multi-scale Residual Blocks (3DMRBs) and a Transformer-based fusion network. This model leverages self-attention mechanisms to capture multi-modal correlations and achieves 94.61% accuracy on the ADNI dataset. The study’s future work aims to optimize the model for clinical deployment, improve computational efficiency, and expand it to include Mild Cognitive Impairment (MCI) detection.

This multi-modal approach illustrates the potential of foundation models to handle complex, multi-dimensional healthcare data (Miao et al. 2024). This study (Wu et al. 2025) presents a foundation model-inspired multimodal framework for Alzheimer’s disease diagnosis by combining transfer learning, Transformer encoders, and LSTM networks. Leveraging ResNet50, pretrained on large datasets, as a feature extractor exemplifies the use of foundation models in medical imaging. The model processes MRI data from three anatomical views (sagittal, coronal, axial) to capture diverse spatial features, which are refined using Transformer-based architectures that preserve spatial relationships. Sequential patterns are then modeled using LSTMs, enabling temporal and spatial understanding. The NeuroFormer model (Lalawat et al. 2025) classifies EEG signals into AD, FTD, and healthy controls using spectral processing, attention-based feature fusion, and hierarchical capsule routing, achieving 95.8% accuracy. This approach illustrates the promise of advanced, domain-specific models in improving non-invasive diagnostics while emphasizing the importance of interpretability and clinical applicability. Additionally, a Vision Transformer (ViT)-Gated Recurrent Unit (GRU) model (Mahim et al. 2024) is used for AD diagnosis, integrating multiple explainable AI (XAI) techniques, including LIME, SHAP, and attention maps. This approach provides interpretable insights into the model’s predictions, helping clinicians understand brain regions affected by AD, such as hippocampal atrophy and cortical degeneration. The study highlights the importance of transparency in AI-driven diagnosis, suggesting that such explainability can foster trust and pave the way for real-world clinical adoption.

Lastly, a study (Shin et al. 2023) explores the application of Vision Transformers (ViT) for AD classification using 18F-Florbetaben PET images. ViT’s ability to capture long-range dependencies in image data enables it to outperform the CNN-based VGG19 model in binary classification tasks. However, it faces challenges in ternary classification due to data limitations and overfitting. This study underscores the potential of foundation models like ViT in medical imaging while also highlighting the need for larger datasets, advanced fine-tuning, and multimodal integration to improve AD diagnosis accuracy.

Together, these studies highlight the growing role of foundation models in AD research, demonstrating their ability to handle diverse and complex data types while enhancing diagnostic accuracy. These models hold significant promise in advancing the field by providing robust, scalable solutions for AD classification and prediction. Future directions will likely focus on improving model efficiency, integrating multimodal data, and ensuring transparency in AI-driven healthcare applications.

## Discussion

This systematic review aimed to provide a comprehensive analysis of the application of Artificial Intelligence (AI) in Alzheimer’s Disease (AD) research, with a focus on multi-modality approaches. The review adhered to PRISMA guidelines, ensuring a rigorous and transparent selection process. A comprehensive search was conducted across four major databases: Scopus, PubMed, Web of Science, and IEEE Xplore. The search covered studies published between 2010 and 2024 to capture the most recent advancements in AI applications for AD. The inclusion criteria were designed to capture studies that used AI methods, such as machine learning, deep learning, and neural networks, in addressing key aspects of Alzheimer’s disease, including diagnosis, progression prediction, detection, monitoring, treatment, and drug discovery.

Emphasis was placed on studies employing multi-modality approaches that integrate neuroimaging, biomarkers, cognitive and genetic data to enhance the predictive capabilities of AI models. After screening a total of 2,005 records, and applying the exclusion criteria (e.g., non-human subjects, single-modality approaches), 156 studies were included in the final review. The methodology ensured that the selected studies were peer-reviewed, offering a high-quality dataset for analysis.

### Implications of neural networks in Alzheimer’s research

Neural networks have significantly advanced Alzheimer’s research by enabling accurate detection, classification, and prediction of disease progression. Deep learning models such as CNNs, RNNs, and transformers extract complex patterns from neuroimaging, cognitive, and genetic data, offering clinical value in early detection and risk stratification (Li et al. 2023; Xu et al. 2023). Multimodal fusion of MRI, PET, CSF, genetics, and digital biomarkers further improves diagnostic performance (Calhoun and Sui 2016). However, real-world adoption remains constrained by challenges of interpretability, generalizability, and regulatory concerns, underscoring the need for explainable AI (XAI), external validation, and bias mitigation.

### Trends in AI adoption and research growth in Alzheimer’s disease

AI-related Alzheimer’s publications rose sharply from 2010 to 2024, with a marked shift toward multimodality after 2018 (Fig. 4-5). By 2024, 239 studies employed multimodal approaches, reflecting their growing importance for robust and accurate models. Domain distribution (Fig. 7) shows detection and diagnosis dominate (71.9%), while disease progression (19.3%), therapeutic discovery (5.5%), and patient care (3.3%) remain underrepresented, highlighting critical opportunities for future exploration.

### Unique contribution: layered framework of AI in Alzheimer’s disease research

This review introduces a Layered Framework categorizing AI applications into four domains: Early Detection, Disease Progression, Therapeutic Discovery, and Real-World Integration. Unlike prior reviews that discuss isolated applications, this framework emphasizes the continuum of AI’s role in Alzheimer’s research and clarifies underexplored areas, especially Patient Care. By identifying these gaps, the framework provides both a conceptual lens and a call to action for more balanced AI research that extends beyond detection to practical, patient-centered integration.

### Challenges and limitations

#### Model generalizability and dataset bias

A central challenge in applying AI to Alzheimer’s research is the limited generalizability of models across diverse populations and clinical settings. While many models achieve strong performance on benchmark datasets such as ADNI, they often fail when tested on external cohorts like NACC or J-ADNI. This is largely due to dataset bias, as most training data are skewed toward well-curated, homogeneous populations, with overrepresentation of white individuals and underrepresentation of minority groups (Gurevich et al. 2023; Banda et al. 2023). Table 20 highlights the demographic imbalances across commonly used datasets, which not only constrain model performance but also risk exacerbating healthcare disparities.

**Table 20:** Dataset names and corresponding publication counts from 156 review papers

Dataset name	Publication count	Dataset name	Publication count
ADNI	110	Kaggle	2
Hospital data	5	Kaggle + TranBioInfoLab + Alzheimer’s Classification EEG	1
ADNI + Hospital data	4	TADPOLE	2
ADNI + AIBL	2	TADPOLE + Cora + Citeseer + PubMed	1
ADNI + Kaggle	2	ADReSS Challenge	2
ADNI + NACC	1	Synthetic Data	2
ADNI + OASIS	1	ADRC	1
ADNI + AddNeuroMed	1	I-CONECT	1
ADNI + J-ADNI	1	DementiaBank Pitt Corpus	1
ADNI + CLAS	1	User Study	3
ADNI + AIBL + NACC	1	Hi-C + FANTOM5	1
ADNI + AIBL + MCAD	1	DrugBank + PIN data	1
ADNI + AIBL + OASIS + MIRIAD	1	DrugBank + TTD + PharmGKB + repoDB + ChEMBL + BindingDB	1
ADNI + AIBL + OASIS + MIRIAD + NACC	1	AMP-AD + ROSMAP + MAYO + MSBB	1
OASIS	2	UofR dataset + UNBC-McMaster dataset	1
OASIS + Kaggle + NCBI	1	–	–

Technical factors also play a role: variability in imaging protocols, biomarker thresholds, and cognitive assessments across cohorts introduces inconsistencies that models trained on narrow datasets cannot adapt to. This overfitting undermines clinical translation and raises ethical concerns, as models may underdiagnose underrepresented groups or misinterpret signals outside of the training distribution.

Addressing this intertwined challenge requires both methodological and structural solutions. On the methodological side, domain adaptation techniques such as adversarial training, transfer learning, and fairness-aware learning algorithms can reduce bias and improve robustness. On the structural side, federated learning and international data consortia offer pathways to expand datasets while preserving privacy and improving representativeness. Public–private partnerships could further facilitate access to diverse cohorts, enabling the development of models that are not only accurate but also equitable.

To ensure trustworthy deployment, regulatory frameworks and ethical guidelines should govern dataset representativeness, validation across subgroups, and transparent communication of model limitations. By addressing dataset bias as the root cause of poor generalizability, Alzheimer’s AI research can move closer to building models that are both technically robust and ethically sound.

#### Explainability vs. performance trade-off

Despite the superior predictive accuracy of deep learning models compared to traditional machine learning methods, their “black-box” nature limits clinical trust and widespread adoption (Hassija et al. 2024). Clinicians require interpretable AI systems that justify predictions based on established biomarkers and cognitive features, ensuring transparency in decision-making. While techniques such as SHAP (Shapley Additive Explanations), attention mechanisms, and Explainable Boosting Machines (EBMs) offer promising avenues for AI interpretability, seamlessly integrating them into high-performance deep learning architectures remains a challenge.

Several approaches have demonstrated potential clinical utility. For instance, saliency map visualizations (Guo et al. 2024) highlighting hippocampal and cortical regions have been prospectively evaluated with neurologists, improving diagnostic confidence in early AD detection. Similarly, prototype-based explanations and counterfactual reasoning have shown value in pilot studies involving clinician feedback, though their scalability to large cohorts is still limited (Abbas et al. 2025). These examples indicate that XAI tools can support clinical decision-making when rigorously validated in real-world settings.

Future research should prioritize hybrid AI models that combine the predictive strength of deep learning with the interpretability of frameworks such as XGBoost-enhanced CNNs. Moreover, clinician-in-the-loop AI systems, where medical experts refine and validate AI-generated diagnoses, hold promise for bridging the gap between algorithmic transparency and clinical usability. Ultimately, the development and prospective testing of explainability methods with healthcare professionals will be critical for ensuring both accuracy and trustworthiness in real-world integration.

### Gaps in knowledge and recommendations

#### Key gaps in current AI-based Alzheimer’s research

While the majority of AI applications in Alzheimer’s research have centered on early detection and diagnosis, domains such as Patient Care and Therapeutic Discovery remain comparatively underrepresented. This imbalance reflects both the relative availability of diagnostic imaging datasets and the structural complexity of designing interventions, yet it also highlights broader methodological and translational challenges that must be addressed for the field to mature.

Despite significant advancements in AI applications for Alzheimer’s research, several critical gaps remain. A major limitation is the reliance on single-time-point data rather than longitudinal modeling, which restricts the ability to track disease evolution over time. Additionally, while AI models perform exceptionally well on research-grade datasets such as ADNI and OASIS, they rarely undergo rigorous validation in real-world clinical environments, reducing their translational impact. Another challenge lies in multi-modal data fusion, as the effective integration of neuroimaging, genetics, and cognitive assessments remains a technical hurdle. Furthermore, current AI models largely overlook non-neuronal aging factors, such as vascular dysfunction, inflammation, and metabolic changes, despite their established role in Alzheimer’s pathology. The predictive power of AI for preclinical Alzheimer’s also remains limited, as existing models excel in classifying mild cognitive impairment (MCI) conversion to AD but struggle to detect asymptomatic individuals at risk of developing the disease.

In addition to these technical gaps, the translation of AI models into clinical practice faces substantial obstacles. Integration with electronic health record (EHR) systems remains limited, as most AI pipelines are developed in isolation from hospital information infrastructures. Regulatory approval pathways (e.g., FDA and EMA requirements) are lengthy and complex, while reimbursement models for AI-assisted diagnostics are not yet standardized, creating uncertainty for healthcare providers. Pilot implementations of AI-based cognitive screening and dementia risk stratification tools are underway (Al-Hindawi et al. 2025), and a small number of clinical trials are testing AI-enhanced decision support for dementia care, but evidence from prospective, multi-site deployments remains scarce.

Moreover, hardware and computational constraints can limit deployment of deep learning models in clinical settings, particularly in resource-limited hospitals. Clinician acceptance is another barrier, as end-users often require interpretable models, integration into existing workflows, and clear evidence of clinical benefit before adoption. Ethical and legal considerations, including data privacy, informed consent, and liability for AI-assisted decisions, further complicate real-world translation.

Taken together, these gaps explain why AI research in Alzheimer’s disease remains concentrated on early detection, while Patient Care and Therapeutic Discovery lag behind. Addressing these limitations will require not only methodological innovations but also structural and regulatory solutions; an agenda that is explored in the following section on recommendations for future research.

#### Recommendations for future research

To address current gaps, future research should prioritize longitudinal AI models that integrate multi-time-point imaging, biomarker, and clinical data to better capture disease trajectories. Expanding globally representative datasets, particularly including underrepresented ethnic and socioeconomic groups, is essential for improving fairness and external validity. The adoption of multi-omics frameworks, combining genetic, metabolic, and epigenetic information can further refine risk prediction and disease modeling.

Improving model explainability remains critical for clinical translation, with methods such as SHAP values, saliency maps, and clinician-in-the-loop systems offering practical approaches. Deployment challenges should also be addressed by designing computationally efficient models suitable for routine clinical hardware, developing clinician-friendly interfaces, and incorporating human factors research to enhance adoption. Early collaboration with regulatory bodies can streamline approval processes and ensure compliance with privacy and safety standards.

Importantly, future work should expand beyond detection toward Patient Care (e.g., remote monitoring, caregiver support, digital health tools) and Therapeutic Discovery (e.g., drug repurposing, target identification), which remain underexplored but clinically critical. Pilot studies and prospective trials in real-world settings will be essential to validate these applications and guide implementation strategies. Finally, embedding AI into scalable digital health technologies, including smartphone-based cognitive assessments, voice biomarkers, and wearable sensors offers a practical path for real-time screening, personalised monitoring, and early intervention across diverse populations.

#### Practical recommendations for new researchers

For researchers newly entering the field of AI in Alzheimer’s research, we provide the following practical recommendations as a concise starting point:

- Datasets (widely used, openly available):
  - ADNI (Alzheimer’s Disease Neuroimaging Initiative) – benchmark multimodal dataset (MRI, PET, clinical, genetics).
  - OASIS – structural MRI data for aging and dementia studies.
  - AIBL – longitudinal neuroimaging and biomarker data.
  - NACC – large clinical registry with cognitive and neuropathological data.
  - OpenNeuro – EEG/MEG and other modalities increasingly used for dementia research.
- Clinical endpoints (commonly modeled):
  - Diagnosis/classification: Alzheimer’s disease vs. mild cognitive impairment (MCI) vs. cognitively normal.
  - Prognosis: MCI-to-AD conversion prediction, time-to-conversion modeling.
  - Cognitive function decline: MMSE, CDR, ADAS-Cog scores.
  - Biomarker-based endpoints: amyloid/tau PET positivity, CSF biomarker thresholds.
- Open-source tools and frameworks (2024):
  - MONAI – medical imaging deep learning framework.
  - Nilearn – neuroimaging machine learning in Python.
  - MNE-Python – EEG/MEG analysis.
  - PyTorch/TensorFlow – deep learning foundations.
  - scikit-learn – machine learning baselines and reproducibility.
  - SHAP, Captum – model interpretability and explainability.

These resources provide a pragmatic foundation for early projects, ensuring alignment with community standards, access to high-quality data, and reproducible, clinically meaningful AI research.

#### Future of longitudinal modeling

As outlined in Section 4.5.2, one of the most critical research priorities is the development of longitudinal AI models that integrate multi-time-point imaging, biomarker, and clinical data. Alzheimer’s disease is inherently progressive, yet much of the current AI research relies on cross-sectional data that captures only a single snapshot in time. While cross-sectional approaches are valuable for early-stage biomarker identification and feasibility testing, they fail to fully represent the temporal complexity of disease evolution. Longitudinal modeling offers a more realistic framework by tracking changes in imaging, biomarkers, cognition, and function across multiple time points, enabling the study of within-subject trajectories and inter-individual variability.

Emerging AI methods—including recurrent neural networks (RNNs), temporal convolutional networks, transformers, and survival analysis models—are well-suited to learn from sequential data and predict individualized disease progression. These methods can support more accurate prognosis, patient stratification for clinical trials, and evaluation of therapeutic responses over time. However, challenges remain, including missing follow-up data, cohort attrition, and heterogeneity in measurement intervals across studies. Addressing these barriers will require harmonization of longitudinal datasets, development of robust imputation techniques, and adoption of federated learning strategies to facilitate multi-cohort modeling without compromising data privacy.

In the future, embedding longitudinal modeling into clinical workflows could transform patient care by enabling dynamic risk assessments, real-time monitoring of treatment effectiveness, and early detection of deviations from expected disease trajectories. As multi-time-point datasets grow and computational methods mature, longitudinal AI models will become essential for advancing precision medicine in Alzheimer’s disease.

### Opportunities for future research

Beyond these immediate priorities, AI presents transformative opportunities for re-shaping Alzheimer’s research. One promising direction is the development of AI-guided personalized interventions, where models not only predict disease risk but also recommend individualized lifestyle, pharmacological, and cognitive strategies. The use of unsupervised deep learning approaches, such as clustering, could enable the discovery of distinct Alzheimer’s subtypes, advancing precision medicine. Federated learning frameworks, which allow for secure decentralized training across multiple institutions, offer a powerful mechanism to preserve privacy while enhancing generalizability. In parallel, hybrid AI–mechanistic models that integrate deep learning with biologically informed representations, such as tau propagation networks or vascular dysfunction pathways, may provide novel insights into Alzheimer’s pathology. Finally, AI-augmented clinical trials, leveraging data-driven patient recruitment, response prediction, and monitoring through digital biomarkers, hold the potential to accelerate therapeutic discovery and validation.

### Extrapolating current research trajectories

To complement the qualitative review of the literature, this section provides a quantitative illustration of the momentum in different AI research domains. It is crucial to note that this analysis is an illustrative extrapolation, not a formal prediction of the future. The primary purpose is to visualize the potential continuation of trends observed in the historical data. The models used are based solely on past publication counts and are inherently unable to account for critical external factors that shape the research landscape, such as funding priorities, unexpected scientific breakthroughs, or global policy shifts.

#### Forecasting methodology and validation

Annual publication counts for four key research categories were collected from the Web of Science database for the period of 2015 to 2024. To establish a robust and defensible forecasting approach, a validation framework was established to select the most suitable model. The dataset was split into a training set (2015-2023) and a hold-out test set (2024). A one-year hold-out period was chosen as a pragmatic compromise to ensure that the recent, rapid emergence of the ’Emerging Techniques’ trend was included in the training data, allowing for a meaningful model evaluation.

Three distinct time-series models were benchmarked to evaluate their predictive performance on a hold-out validation set-

1. A Long Short-Term Memory (LSTM) network, configured with a single layer of 50 ReLU-activated units, a 20% dropout rate, and trained for up to 500 epochs with the Adam optimizer using an early stopping criterion.
2. An Autoregressive Integrated Moving Average (ARIMA) model, a standard statistical baseline, configured with an order of (1,1,1).
3. The Prophet forecasting tool, with its default trend-fitting capabilities and seasonality disabled for this annual data. Model performance was evaluated against the 2024 hold-out set using Root Mean Squared Error (RMSE).

**Table 21:** Model performance on hold-out year (2024)

Category	Category name	Actual (2024)	LSTM prediction (RMSE)	ARIMA prediction (RMSE)	Prophet prediction (RMSE)
1	Traditional Machine Learning	315	391.04 (76.04)	294.00 (21.00)	346.96 (31.96)
2	Deep Learning: Convolutional Neural Networks	322	223.03 (98.97)	304.46 (17.54)	285.54 (36.46)
3	Deep Learning: Sequential and Generative	74	59.44 (14.56)	73.78 (0.22)	76.07 (2.07)
4	Emerging Techniques	125	7.29 (117.71)	139.92 (14.92)	56.40 (68.60)

#### Model comparison and final projections

The validation benchmark revealed a clear performance difference among the models. As summarized in Table 21, the simpler ARIMA model consistently and significantly outperformed both the LSTM and Prophet models across all four research categories, demonstrating the highest accuracy.

#### Forward extrapolations (2025–2030)

**Fig. 12:**
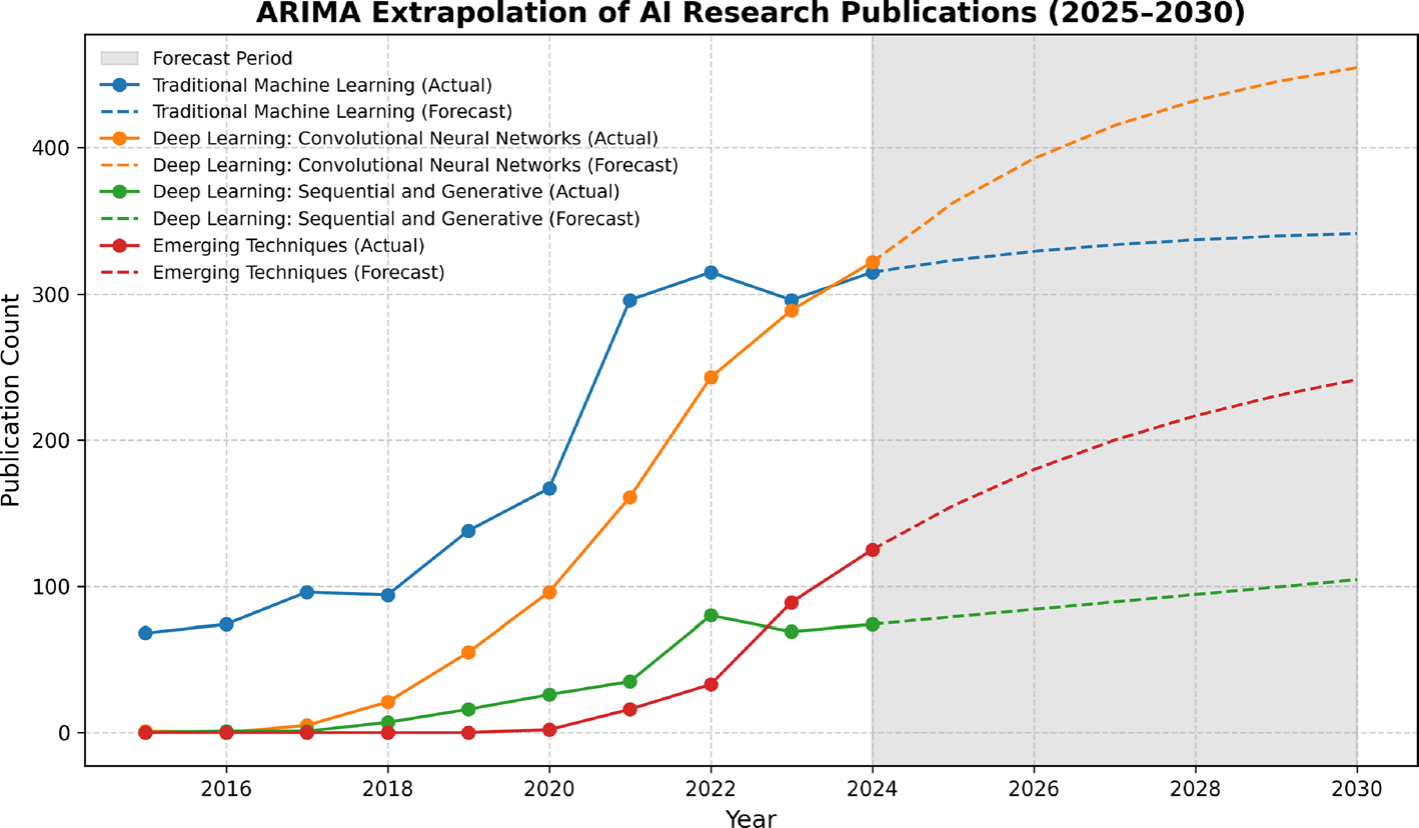
Extrapolated publication trends in AI-based Alzheimer’s research from 2015 to 2030, based on Web of Science data. Historical values (2015–2024) are shown as solid lines, while ARIMA-based projections (2025–2030) are indicated as a shaded forecast region

Based on superior validation performance, ARIMA was selected as the primary model for forward extrapolation. Projections for 2025–2030 are presented in Table 22 and Fig. 12 presents both historical and extrapolated publication trends (2015–2030), offering insights into the evolving landscape of AI techniques in Alzheimer’s research. These indicate a steady plateau for Traditional Machine Learning, continued strong growth in Deep Learning: Convolutional Neural Networks, moderate expansion of Deep Learning: sequential and Generative, and a marked upward trend in Emerging Techniques.

**Table 22:** ARIMA forecasts for 2025–2030

Year	Traditional machine learning	Deep learning: CNNs	Deep learning: sequential and generative	Emerging techniques
2025	323.19	362.24	79.07	155.09
2026	329.28	392.48	84.14	179.78
2027	333.83	415.21	89.20	200.03
2028	337.21	432.30	94.27	216.65
2029	339.73	445.14	99.33	230.28
2030	341.61	454.80	104.40	241.47

#### Interpretation of alternative scenarios

Although ARIMA provides a conservative and stable baseline, comparison with alternative models illustrates the range of plausible futures. Prophet generally forecasts slightly higher values than ARIMA, projecting, for example, more than 530 publications in Deep Learning: Convolutional Neural Networks by 2030 and around 150 in Emerging Techniques. LSTM, in contrast, amplifies exponential patterns in the data, yielding markedly higher estimates—particularly for Emerging Techniques, where it suggests growth beyond 780 publications by 2030.

Taken together, these findings suggest that *Category 1* is approaching saturation, *Category 2* will likely continue as the leading research stream, *Category 3* will grow steadily but modestly, and *Category 4* represent the most uncertain and potentially transformative area. The divergence between models underscores the importance of interpreting quantitative extrapolations with caution, particularly for rapidly evolving research fronts where historical baselines remain limited.

## Conclusion

This systematic review presents a comprehensive analysis of the role of AI in AD research, with a particular focus on multimodality approaches. By adhering to PRISMA guidelines, we ensured a rigorous methodology for identifying and analyzing studies published between 2010 and 2024. The findings confirm AI’s transformative impact on AD research, particularly in early detection, disease progression modeling, and therapeutic discovery. However, a notable underexplored area is the integration of AI into patient care and real-world settings, which presents a significant opportunity for future research.

A key contribution of this review is the introduction of a Layered Framework for analyzing AI applications in AD. This framework categorizes the use of AI into four progressive domains: Early Detection, Disease Progression, Therapeutic Discovery, and Real-World Integration (Patient Care). This structure provides a novel perspective, offering a more holistic understanding of AI’s evolving role in addressing Alzheimer’s. Unlike existing reviews, which often discuss AI in isolated domains, our layered approach emphasizes the interconnectedness of these domains and highlights areas where AI can make the greatest impact. Additionally, this review introduces a forecasting analysis using ARIMA-based time series models to project publication trends through 2030. This predictive lens reveals that emerging AI methods, such as generative models and transformer-based architectures, are expected to grow significantly, pointing to future directions where innovation is likely to concentrate. This forward-looking perspective helps identify not only current gaps but also upcoming opportunities for impactful research.

While the literature demonstrates substantial progress in early diagnosis and disease modeling, there remains a critical gap in the real-world application of AI, particularly in patient care. Future research should focus on developing AI systems that integrate clinical, cognitive, behavioral, and environmental data to support personalized care and treatment management. To guide the field, we propose a concrete research agenda: (1) establish multi-center federated repositories with standardized imaging and clinical protocols, (2) conduct prospective trials of AI-assisted cognitive assessments in primary care, (3) develop open-source XAI toolkits validated by clinician end-users, and (4) explore AI-driven adaptive clinical trial designs for AD therapeutics. By advancing our understanding of AI’s capabilities across these layers, and by forecasting where the field is headed, we aim to catalyze research that bridges the gap between innovation and implementation. The integration of AI into these domains has the potential to revolutionize AD management by facilitating earlier interventions, accelerating therapeutic discovery, and improving outcomes for individuals and caregivers.

In conclusion, this review not only synthesizes the current state of AI in Alzheimer’s research but also offers a strategic and forward-thinking framework, enriched by forecasting insights, to guide future developments. Embracing this multi-layered, data-driven approach will empower researchers and clinicians to address the multifaceted challenges of Alzheimer’s and shape a new era of precision-driven, AI-enabled care.

## Data Availability

All data used in this systematic review were obtained from publicly available sources and peer-reviewed journal articles. The selection process is outlined in the PRISMA flow diagram included in the manuscript. A complete list of included studies is available in the Zotero library used for this review. This library can be shared upon reasonable request to the corresponding author. Any new data generated during this study can be obtained upon request.
